# Three privileged scaffolds, one pipeline: chalcones, pyrazolines and pyrazoles in drug discovery and fluorescent sensing

**DOI:** 10.1039/d6md00424e

**Published:** 2026-08-20

**Authors:** Alexander Ciupa

**Affiliations:** a Materials Innovation Factory, University of Liverpool 51 Oxford Street Liverpool L73NY UK ciupa@liverpool.ac.uk

## Abstract

Chalcones, pyrazolines, and pyrazoles constitute a uniquely powerful discovery pipeline in which each is a privileged scaffold with distinct applications, while also serving as the synthetic precursor to the next. Despite a theoretical chalcone library exceeding 680 000 unique structures accessible from commercial starting materials alone, fewer than 5140 examples have been reported in the literature—representing just 0.8% of this chemical space. Combining this precursor library with the downstream pyrazoline and pyrazole chemical spaces yields a combined landscape conservatively estimated to exceed 7.5 million unique structures, which remains largely unexplored. This review examines leading examples from 1990 to 2025 across all three scaffold classes, with particular emphasis on the most promising applications of this pipeline: anti-cancer, anti-inflammatory, anti-viral, and fluorescent sensing applications. We explore how pharmacophore hybridisation is further expanding this pipeline. Whereas previous reviews have focused on individual scaffold classes in isolation, this review takes an integrated, systematic approach that unifies all three into a single coherent discovery pipeline. We conclude with a roadmap on how the convergence of design of experiments, automated synthesis, high-throughput screening, and machine learning will unlock the potential of this vastly unexplored chemical space.

## Abstract 2

Chalcones, pyrazolines, and pyrazoles constitute a uniquely powerful discovery pipeline in which each is a privileged scaffold with distinct applications, while also serving as the synthetic precursor to the next.

## Graphical abstract

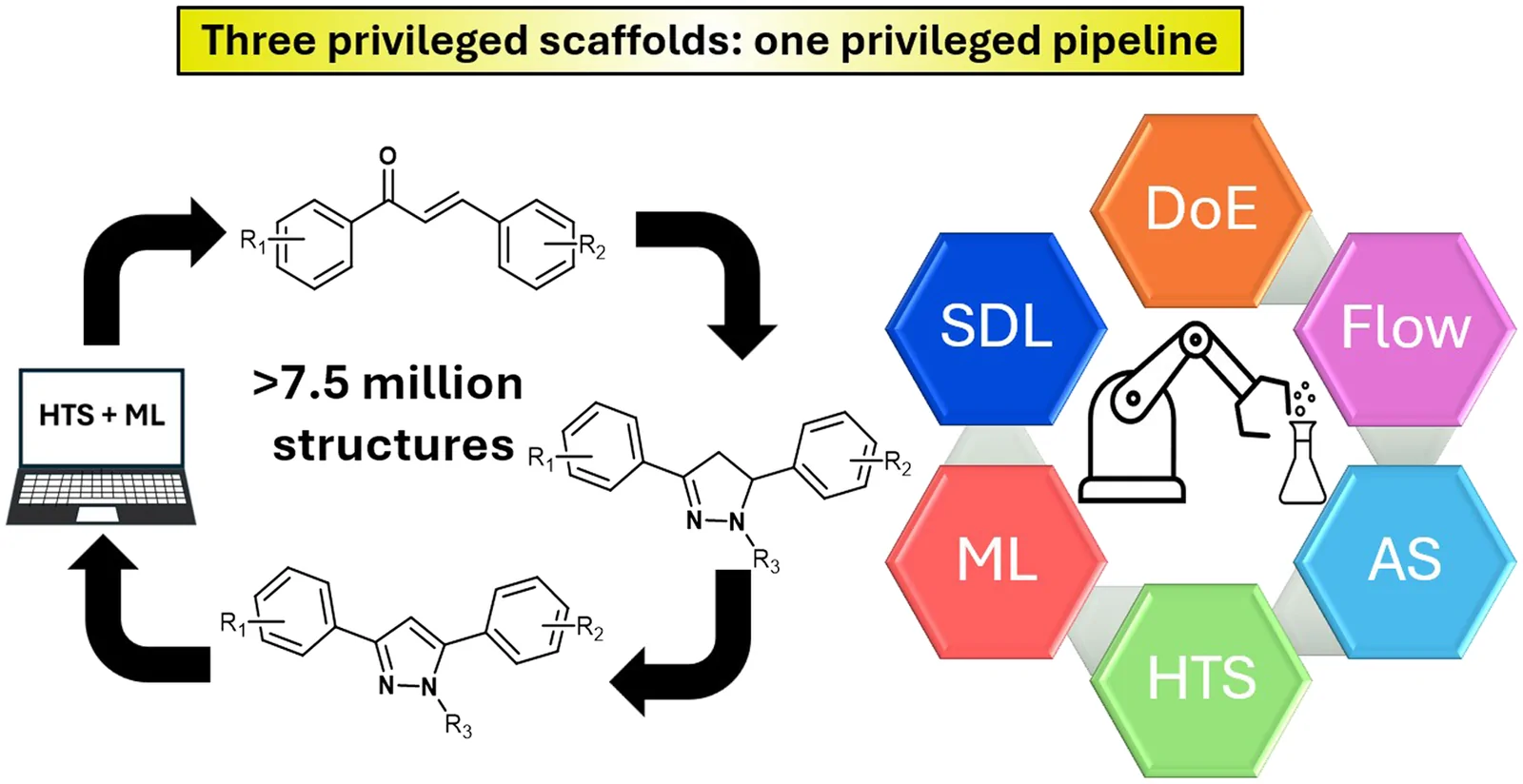

## 1 Introduction

**Fig. 1:**
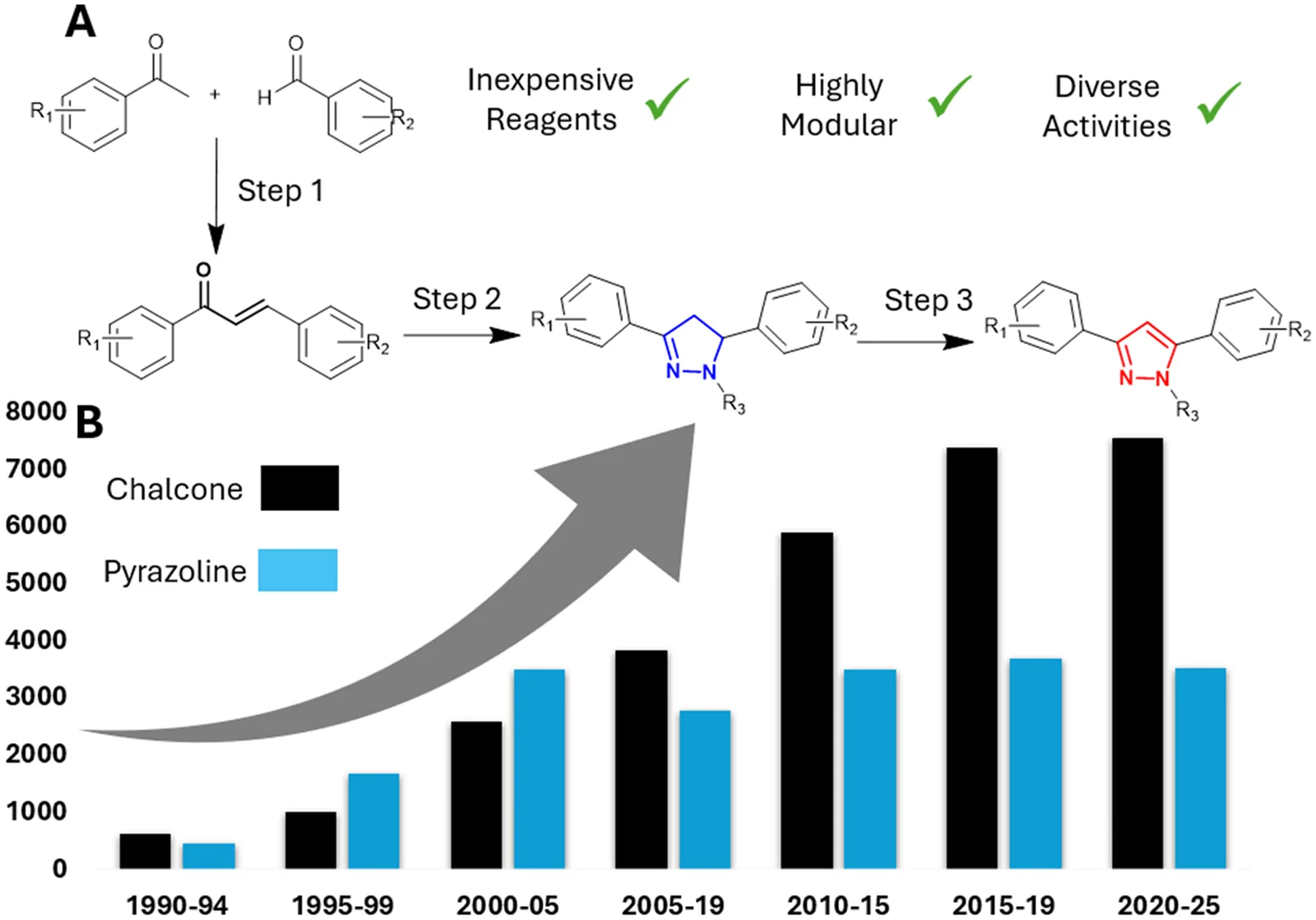
The chalcone, pyrazoline and pyrazole pipeline from inexpensive and highly modular commercially available reagents (panel A) and the growth of chalcones (black bars) and pyrazolines (blue bars) in the literature (panel B).

The identification of new bioactive compounds has historically been a labour-intensive process, driven by the synthesis and screening of collections of structurally related molecules produced largely one at a time through conventional bench chemistry.^1,2^ This trial-and-error approach, while responsible for many of the drugs and functional materials that underpin modern medicine, is inherently expensive and inefficient.^3^ Large areas of chemical space remain either unexplored or effectively inaccessible because of the immense scale of the task involved.^4^ Reymond *et al.* estimated there are 166.4 billion possible molecules composed of up to 17 atoms of C, N, O, S, and halogens.^5^ In stark contrast, approximately 60 million unique molecules have been reported in the last century.^5,6^ The use of novel data-driven techniques is required to explore the potential of this chemical space.^7^ The term privileged scaffold, first coined by Evans *et al.*, describes a molecular framework that can be structurally tuned to produce compounds active against a wide range of biological targets.^8^ Established examples include the benzodiazepine core in the sedative diazepam,^8,9^ the 1,4-dihydropyridine scaffold in nifedipine,^10^ a calcium channel blocker, and the indole ring in the anti-inflammatory drug indomethacin.^11^ This concept of tuneable molecular structures extends to fluorescent sensors for disease diagnosis,^12^ for example, fluorescein^13^ and rhodamine^14^ underpin a range of selective sensors for biological analytes. Chalcones,^15^ pyrazolines^16^ and pyrazoles^17^ (black, blue and red units respectively in Fig. 1A) collectively exemplify this concept with an activity profile spanning medicinal chemistry and fluorescent sensors. All are united by a linear synthetic route: chalcone to pyrazoline,^18^ followed by pyrazoline oxidation to pyrazole.^19^ This transforms three separate scaffolds into a single, directional, and uniquely powerful privileged pipeline for therapeutic discovery (Fig. 1A).

A conservative survey of major reagent suppliers identifies approx. 936 methyl aryl ketones and 731 aromatic aldehydes from commercially available sources (see ESI2), yielding a theoretical chalcone library exceeding 680 000 unique molecules. The synthesis of bespoke reagents expands this potential library further still. Approximately 5140 unique chalcones have been reported representing just 0.8% of theoretical accessible chemical space (ESI2). Conversion of this library to pyrazolines and subsequent pyrazoles provides a chemical space exceeding 7.5 million unique structures. The use of inexpensive and widely available modular reagents offers distinct advantages when designing large chemical space libraries. The growth in publications across all three classes over the past 35 years reflects this potential (see Fig. 1B for chalcones and pyrazolines, ESI1 for pyrazoles). Despite exciting discoveries, the sheer vastness of this chemical space remains largely unexplored. This review will discuss the leading discoveries across all three scaffolds over the past three decades and summarise structural activity relationships (SARs) for each class. Combining these SARs with the latest advances in design of experiments (DoE), automated synthesis (AS), high-throughput screening (HTS), and machine learning (ML) will unlock the unexplored potential of this large chemical space.

## 2 Chalcone synthesis

**Fig. 2:**
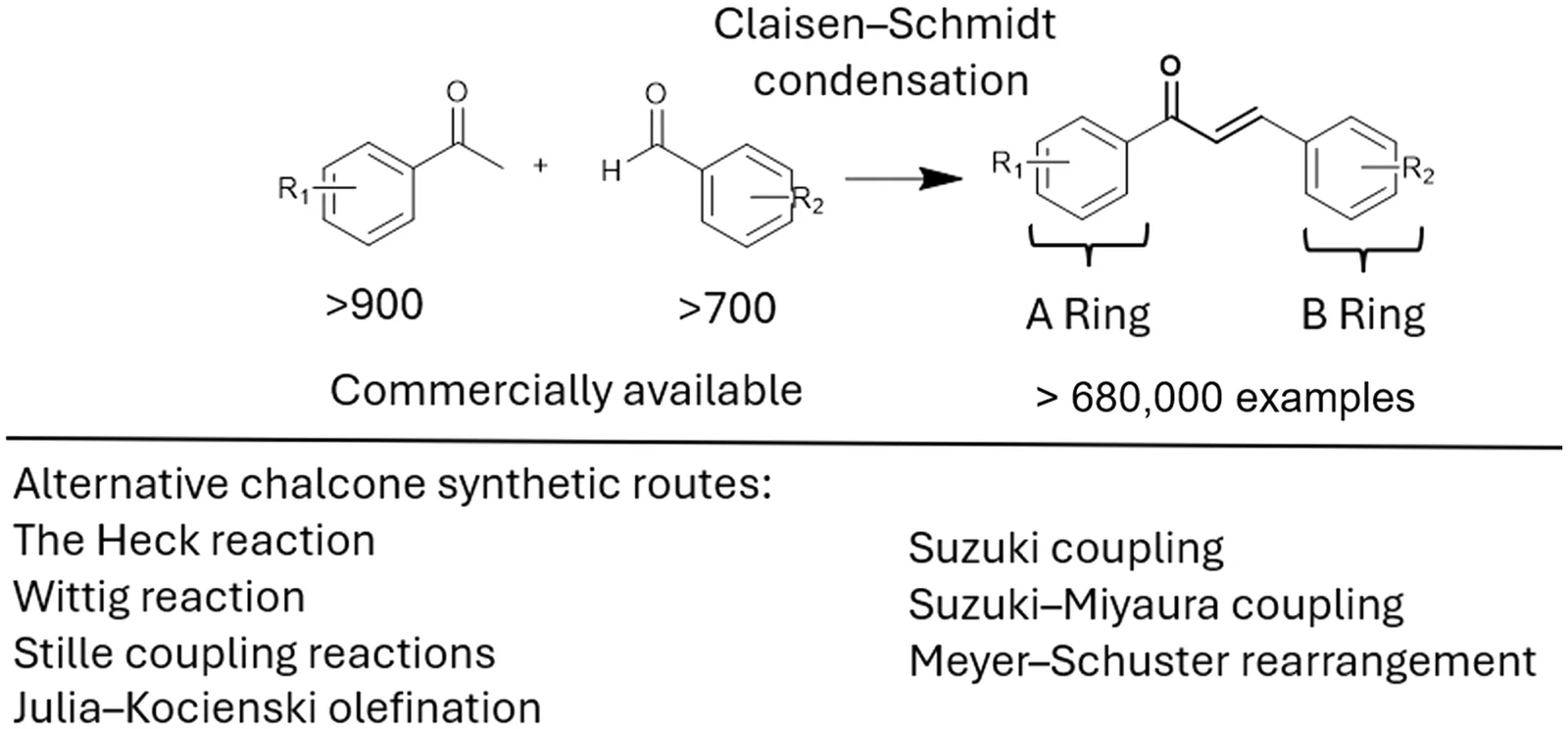
Chalcone formation.

The foundational building block of this pipeline is the 1,3-diphenylprop-2-en-1-one, more commonly known as a chalcone. The term chalcone is derived from the Greek word chalkos, meaning copper or bronze, due to the characteristic colour of these compounds.^20^ The chalcone scaffold exists as two isomeric forms – the thermodynamically more stable *trans* (*E*) isomer and the sterically disfavoured *cis* (*Z*) isomer. Chalcones are accessible through a wide range of carbon–carbon bond-forming strategies, the most extensively employed is the Claisen–Schmidt condensation,^21^ involving an aromatic aldehyde and a methyl aryl ketone derivative (Fig. 2). This reaction can be performed under acid or base catalysis in polar solvent to afford the product in good to excellent yield.^22^ This reaction proceeds *via* enolate formation, nucleophilic attack of the aromatic aldehyde, then E1cB dehydration to generate the conjugated enone product (chalcone).^23^

This reaction is favoured for its low cost, scalability, and compatibility with a range of commercially available electron-rich and electron-deficient starting materials. Beyond Claisen–Schmidt condensation, a broad range of alternative routes including the Heck reaction,^24^ Suzuki and Suzuki–Miyaura cross-coupling,^25,26^ Stille coupling,^27^ Julia–Kocienski olefination,^28^ Wittig reaction,^29^ sequential Hiyama coupling/Narasaka acylation,^30^ Meyer–Schuster rearrangement,^31^ decarboxylative cross-coupling,^32^ and Friedel–Crafts acylation^33^ are available. Beyond medicinal and fluorescent sensing, chalcones are employed in agriculture as herbicidal, fungicidal, and insecticidal agents, in advanced materials science as nonlinear optical chromophores, components of dye-sensitised solar cells, and in the food and cosmetic industries.^34^ One of the most intensively studied applications of chalcones is in the search for novel anti-cancer agents.^35^

## 3 Chalcones as anti-cancer agents

**Fig. 3:**
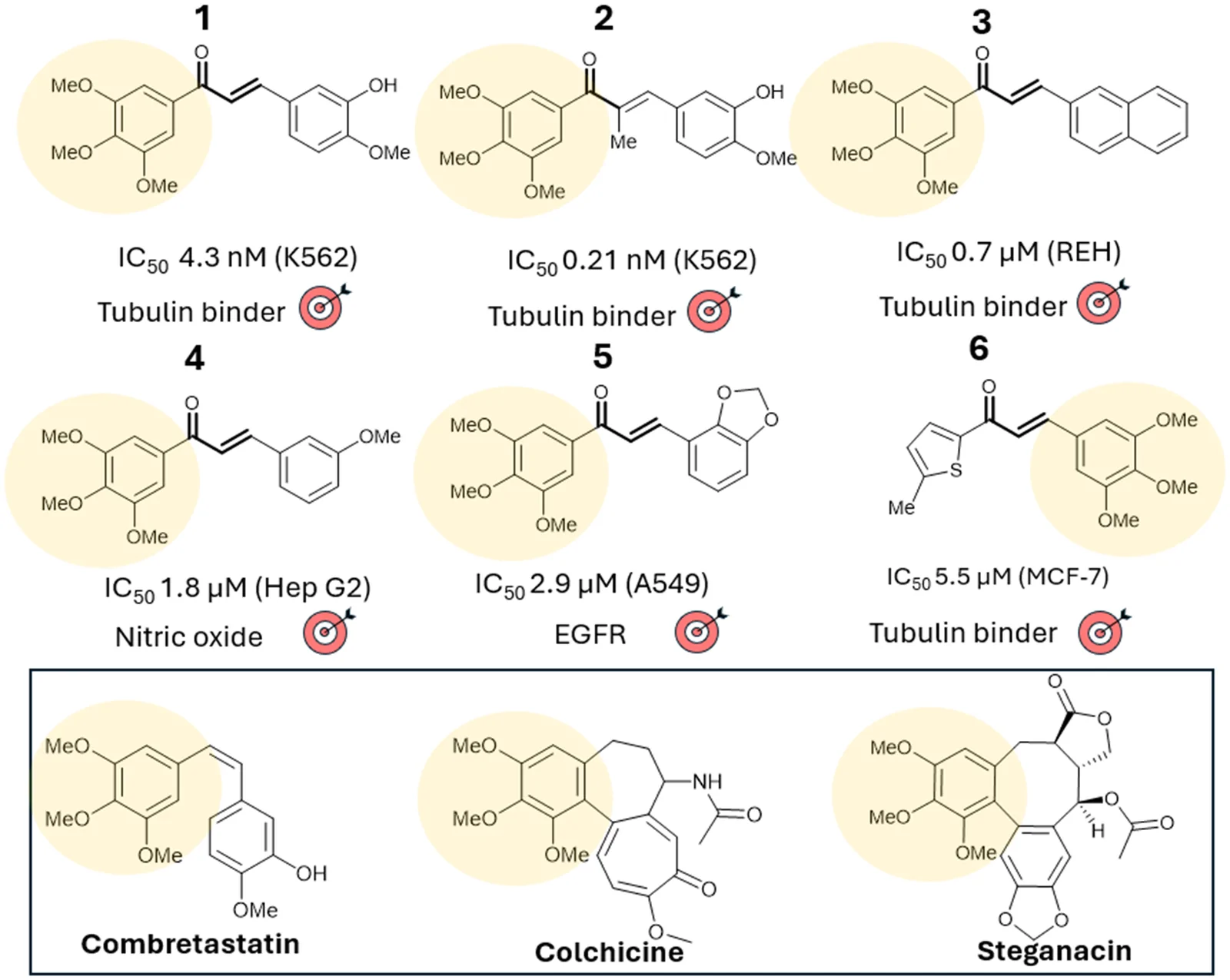
The enone (in bold) in chalcone 1–6 and natural products combretastatin, colchicine and steganacin (inset) containing the 3,4,5-trimethoxyphenyl unit.

Cancer is a broad term encompassing a collection of diseases characterised by the uncontrolled proliferation of abnormal cells arising from gene mutations that disrupt the normal regulation of cell growth, division, and death.^36^ In 2022, approximately 20 million new cancer cases were diagnosed, and 9.7 million deaths worldwide.^37^ Multidrug resistance, in which cancer cells acquire resistance to structurally and mechanistically similar drugs, is estimated to account for approximately 90% of cancer treatment failures.^38^ The search for anti-cancer chalcones emerged as a response to this challenge. One of the most successful chalcones, 1, was reported by Ducki *et al.*, displaying a concentration that inhibited 50% of cell growth (IC_50_) of 4.3 nM in K562 cells, a leukaemia cell line (Fig. 3).^39^

**Fig. 4:**
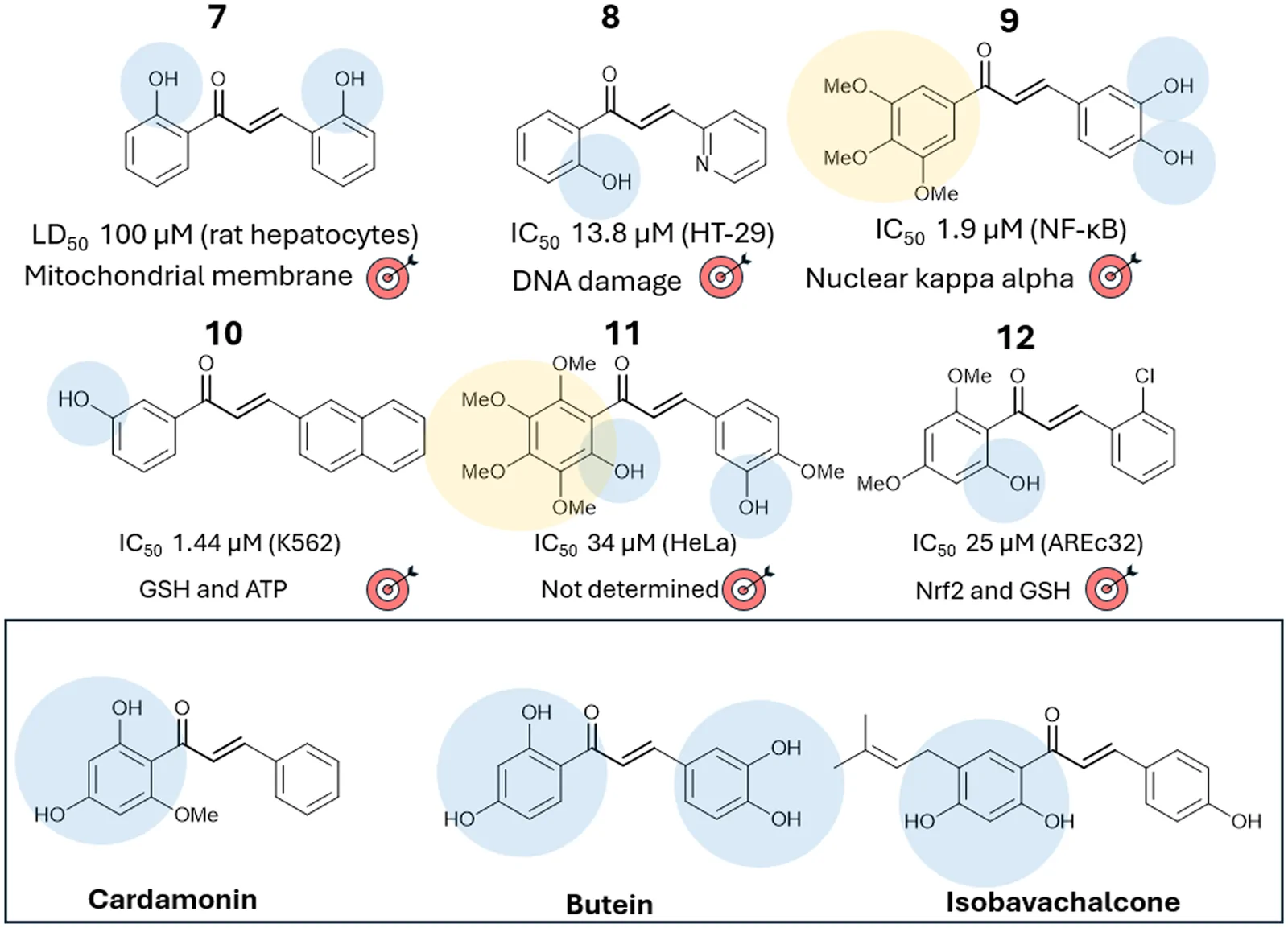
The enone (in bold) in chalcones 7–12 and natural products cardamonin, butein and isobavachalcone (inset) with hydroxyl units.

Analogue 2 with a methyl substituent on the alpha position was approximately 20-fold more active with an IC_50_ value of 0.21 nM, also in K562 cells.^39^ The authors propose that the 3,4,5-trimethoxyphenyl unit, also present in the natural products colchicine, combretastatin, and steganacin (inset in Fig. 3), was critical to the anti-cancer activity. A follow-up study confirmed both 1 and 2 target tubulin, specifically the colchicine binding site on tubulin.^40^ These early results inspired a focused research effort on a range of 3,4,5-trimethoxyphenyl substituted chalcones, for example, Vogt *et al.* synthesised and screened twenty-three chalcones, revealing chalcone 3 with an IC_50_ value of 0.7 μM in REH, a leukaemia cell line (Fig. 3).^41^ Molecular docking and biological studies confirmed 3 was a potent tubulin binder at the colchicine site. Interestingly, the 3,4,5-trimethoxyphenyl unit also extends anti-cancer activity beyond tubulin, for example chalcone 4 inhibits nitric oxide (NO)^42^ and chalcone 5 inhibits epidermal growth factor receptor tyrosine kinase (EGFR-TK)^43^ (Fig. 3). Anti-cancer activity is retained if the 3,4,5-trimethoxyphenyl unit is transferred to the B ring and the introduction of a range of heterocycles, for example thiophene in chalcone 6 (Fig. 3).^44^ Extensive structural activity relationships (SARs) have validated the 3,4,5-trimethoxyphenyl as a potent component of anti-cancer chalcones.^45^ Another widely explored motif is the use of multiple hydroxyl units, also found in chalcone natural products cardamonin, butein, and isobavachalcone (inset in Fig. 4). O'Brien *et al.* performed an SAR on ten hydroxyl chalcones with chalcone 7 the leading contender (Fig. 4).^46^ Hydroxyl units on A and B ring alongside the double bond were essential for anti-cancer response with collapse in mitochondrial membrane potential the primary mode of activity. González *et al.* reported an extensive SAR study on forty-one hydroxyl chalcones, with chalcone 8 the most active in HT-29, a human colon cell line, due to DNA damage (Fig. 4).^47^ A further SAR study by Xing *et al.* on thirty-four chalcones revealed chalcone 9 with two hydroxyl groups on the B and a 3,4,5-trimethylphenyl A ring was the lead compound (Fig. 4).^48^

**Fig. 5:**
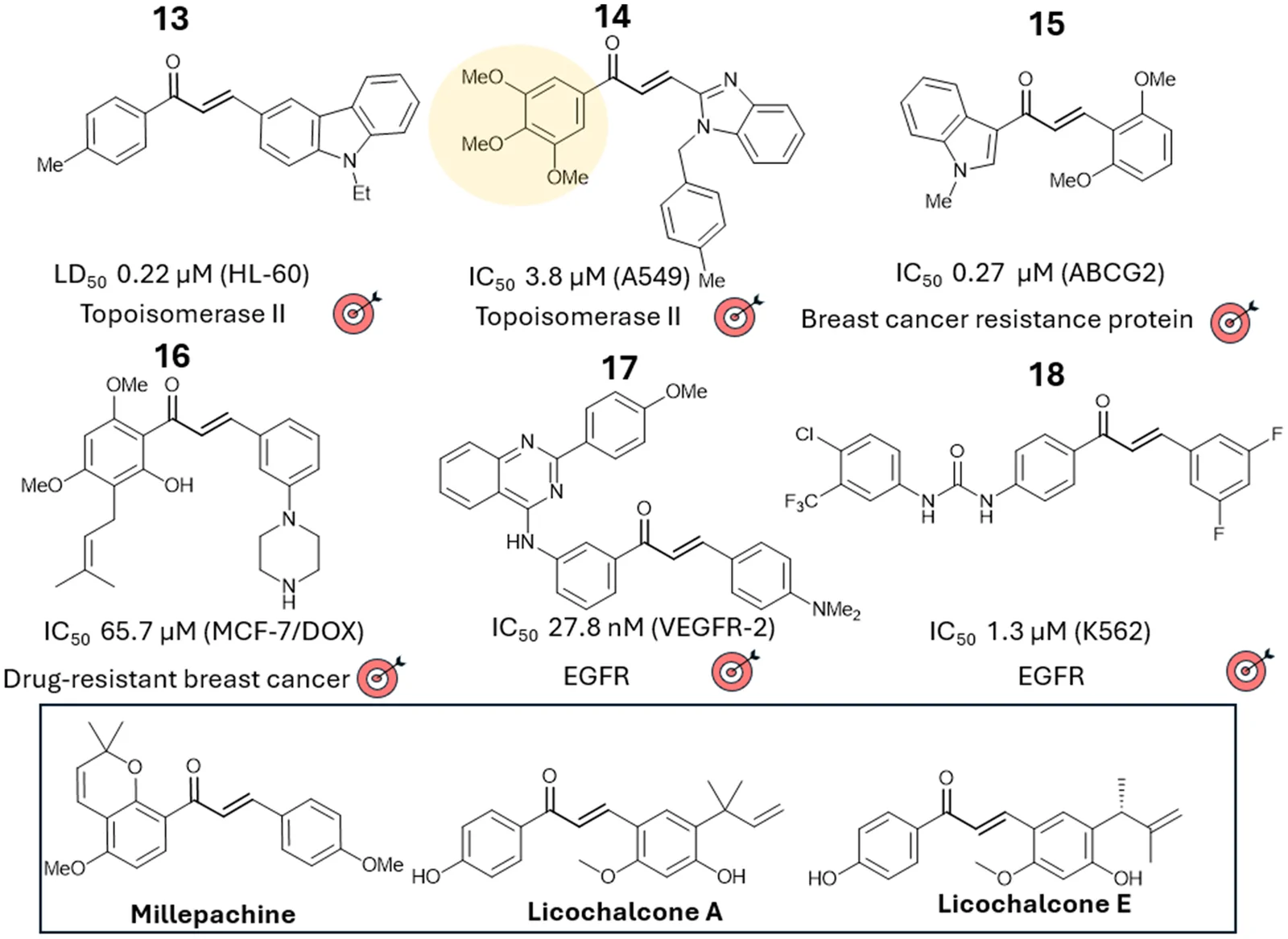
Emerging targets for anti-cancer cancers alongside natural products chalcones, millepachine, licochalcone A and licochalcone E (inset).

The inhibition of nuclear factor kappa was determined as the mode of action.^48^ Chalcone 10, containing a hydroxyl unit on the A ring and naphthyl B ring, displayed anti-cancer activity of IC_50_ 12 μM in B16-F10 cells *via* (glutathione) GSH and adenosine triphosphate (ATP) depletion (Fig. 4).^49^ Two further hydroxylated chalcones are 11 and 12, displaying promising activities in cancer cell lines. The hydroxyl motif is present multiple times in well-established therapeutic chalcones (Fig. 4).^50,51^ The search for new chalcone targets is ongoing, for example, topoisomerase inhibitors. Topoisomerase is an enzyme that cleaves and reconnects DNA strands during DNA replication and is a promising drug target.^52^ Chalcones 13 and 14 were confirmed to display promising IC_50_ values of 0.22 μM and 3.8 μM in cancer cell lines, with the inhibition of topoisomerase as the mode of action (Fig. 5).^53,54^ Two further examples were chalcones 15 and 16 which specifically target drug-resistant cell lines with IC_50_ values of 0.27 μM and 65 μM, respectively (Fig. 5).^55,56^ A further emerging use of chalcones is as epidermal growth factor agents, such as chalcone 17 and 18 (Fig. 5).^57,58^ These highlight the potential of the chalcone privileged scaffold for a broad range of anti-cancer targets. While interesting in themselves, chalcones can serve as precursors to further anti-cancer agents such as flavones.^59^ The use of chalcones as precursors to pyrazolines and pyrazoles will be discussed further.

## 4 Chalcones as anti-inflammatory agents

Inflammation is a natural response to injury or infection, driven in part by prostaglandins produced through the cyclooxygenase enzymes COX-1 and COX-2. Targeting these enzymes offers therapeutic benefit across a range of common conditions, including arthritis, pain, and fever.

**Fig. 6:**
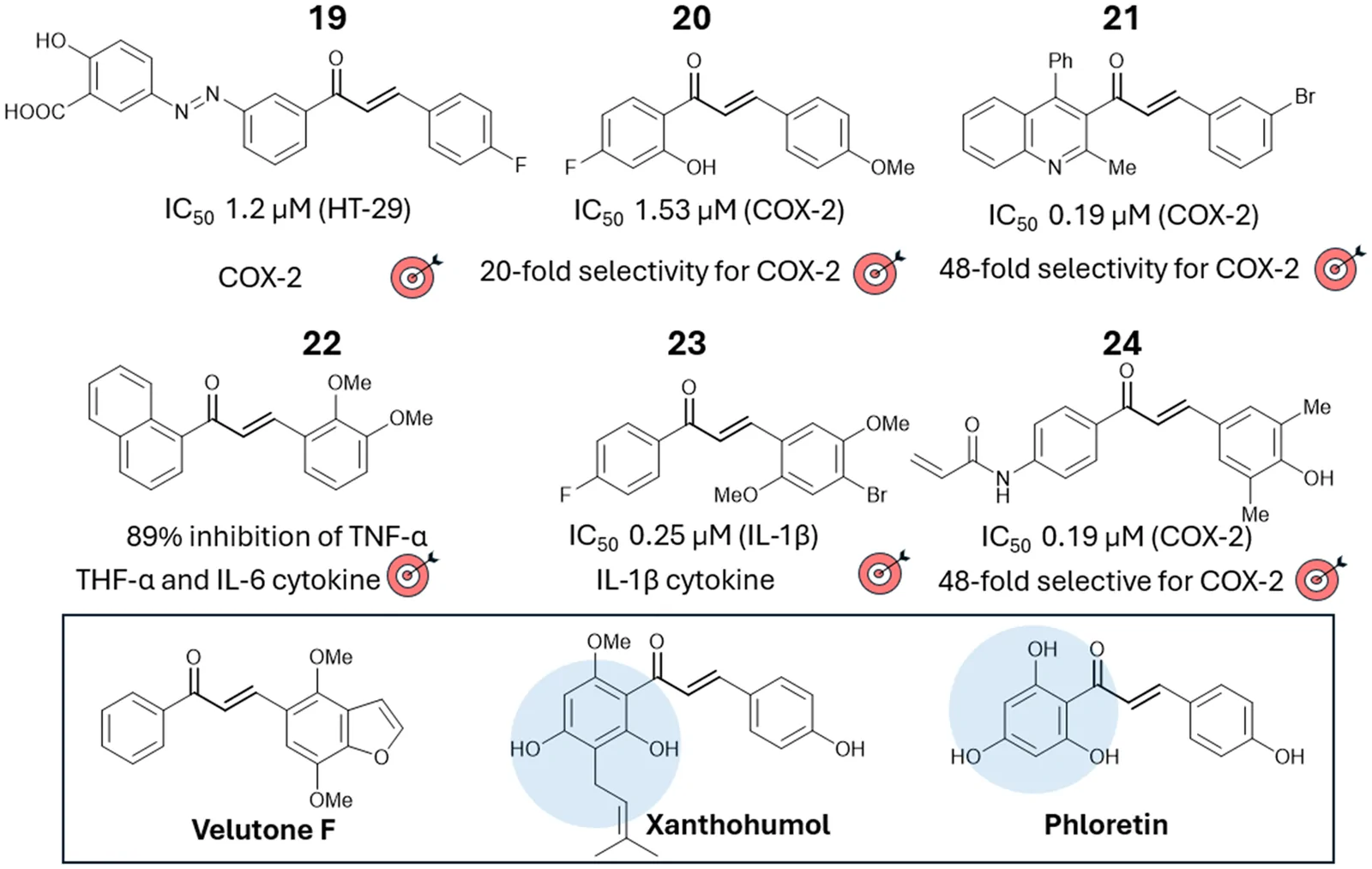
The enone (in bold) in chalcones 19–24 and natural product chalcones velutone F, xanthohumol and phloretin butein and isobavachalcone (inset).

**Fig. 7:**
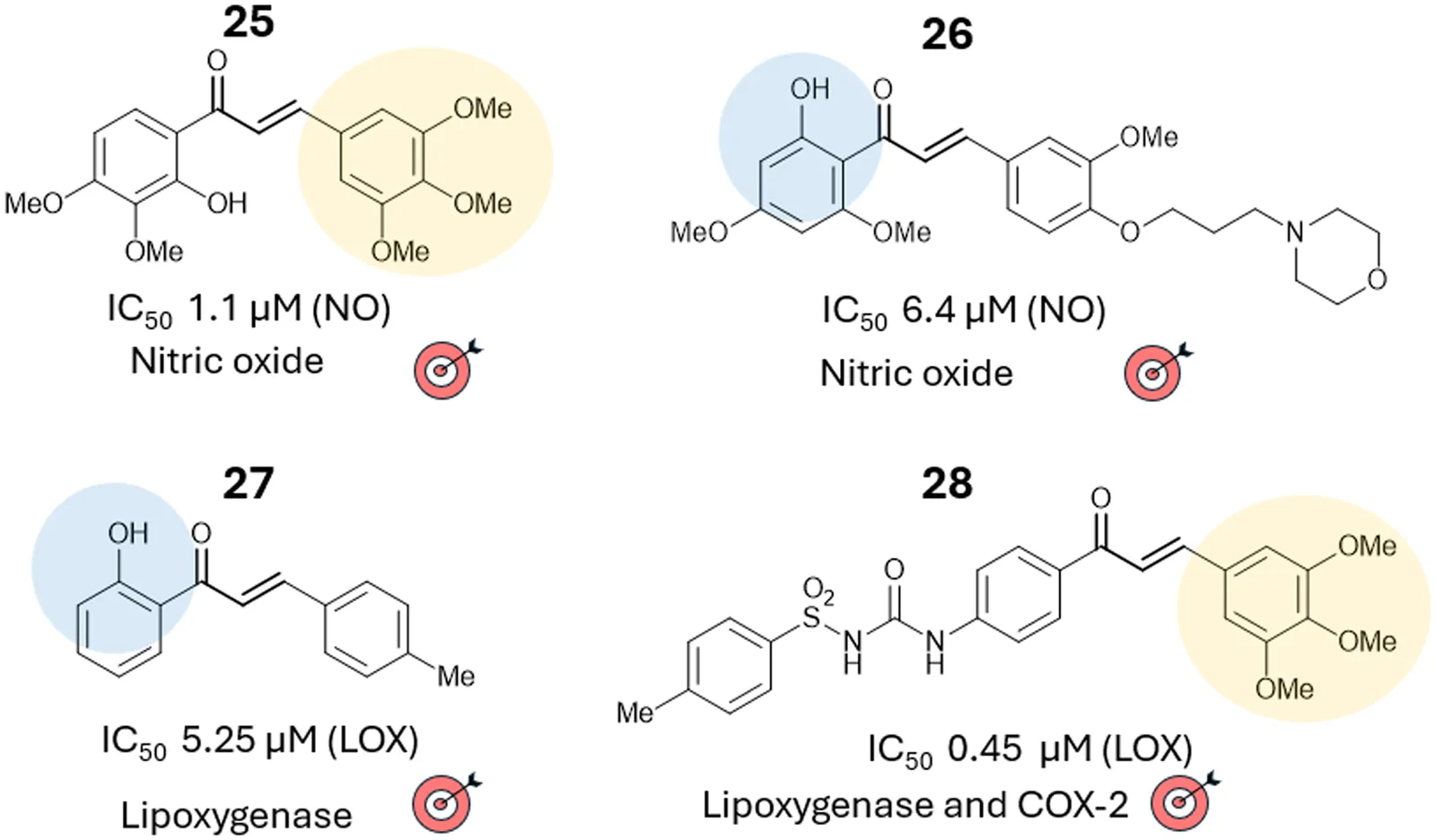
The enone (in bold) within chalcones 25–28 with a range of anti-inflammatory activities reported.

The use of chalcones in the search for novel anti-inflammatory agents, for example the inhibition of cyclooxygenase (COX) enzymes COX-1 and COX-2 is a common pathway.^60^ The chalcone natural products velutone F, xanthohumol, and phloretin are known to display a broad range of anti-inflammatory properties (inset in Fig. 6). A SAR study by Abdelgawad *et al.* on eighteen chalcones resulted in the lead chalcone, 19, with a COX-2 IC_50_ value of 1.27 μM (Fig. 6).^61^ Further studies revealed this compound displayed anti-proliferative activity in HT-29 colon cancer cells. Omar *et al.* screened eight chalcones against COX-1 and COX-2 and discovered chalcone 20 had a twenty-fold selectivity for COX-2 over COX-1 (Fig. 6).^62^ Molecular docking studies confirmed 20 occupied the COX-2 binding site like the licensed COX-2 compound celecoxib. Tan *et al.* synthesised twenty-four chalcones, with 21 displaying potent COX-2 inhibition with an IC_50_ value of 0.19 μM and a 40-fold selectivity for COX-2 over COX-1 (Fig. 6).^63^ Molecular docking studies revealed 21 could bind to both COX-1 and COX-2 binding sites; however, additional hydrogen bonding at the COX-2 binding site was responsible for the selectivity. Another mechanism to reduce inflammation is to target cytokines, for example, tumour necrosis factor alpha (TNF-α). Sylte *et al.* screened a range of chalcones and discovered 22, which inhibited TNF-α and interleukin 6 (Fig. 6).^64^ Inspired by the natural product chalcone velutone F (inset Fig. 6), Ye *et al.* screened over thirty-seven analogues with the discovery of chalcone 23 with very potent activity against Interleukin-1 beta, a pro-inflammatory cytokine (Fig. 6).^65^ Sharma *et al.* devised an extensive SAR on velutone F and discovered chalcone 24 with promising potential against IL-1β with an IC_50_ value of 1.3 μM (Fig. 6).^66^ Further studies revealed that 24 could suppress reactive oxygen species (ROS) and form the basis for further studies for chalcone-based analogues. The reduction of nitric oxide (NO) is a widely explored target; for example, Redda *et al.* discovered chalcone 25 with potent broad-spectrum anti-inflammatory activity (Fig. 7).^67^ Chalcone 25 reduced NO production and a range of other cytokines significantly, with the OH and OMe units vital to activity.

Chen *et al.* developed thirty-nine chalcones with 26 the most potent NO inhibitor with an IC_50_ value of 6.4 μM due to down regulation of nitric oxide synthase (iNOS) protein.^68^ A further anti-inflammatory target is the reduction of Lipoxygenase (LOX) with the study by Kefalas *et al.* a prominent example (Fig. 7).^69^ An extensive SAR study determined simple chalcone 27 as a LOX inhibitor with IC_50_ value of 5.25 μM. A dual LOX and COX-2 inhibitor, chalcone 28, was reported by Ferrándiz *et al.*^70^ The coronavirus 2019 (COVID-19) pandemic highlighted the urgent need for the development of novel anti-viral therapeutics with chalcones attracting considerable attention.

## 5 Chalcones as anti-viral agents

**Fig. 8:**
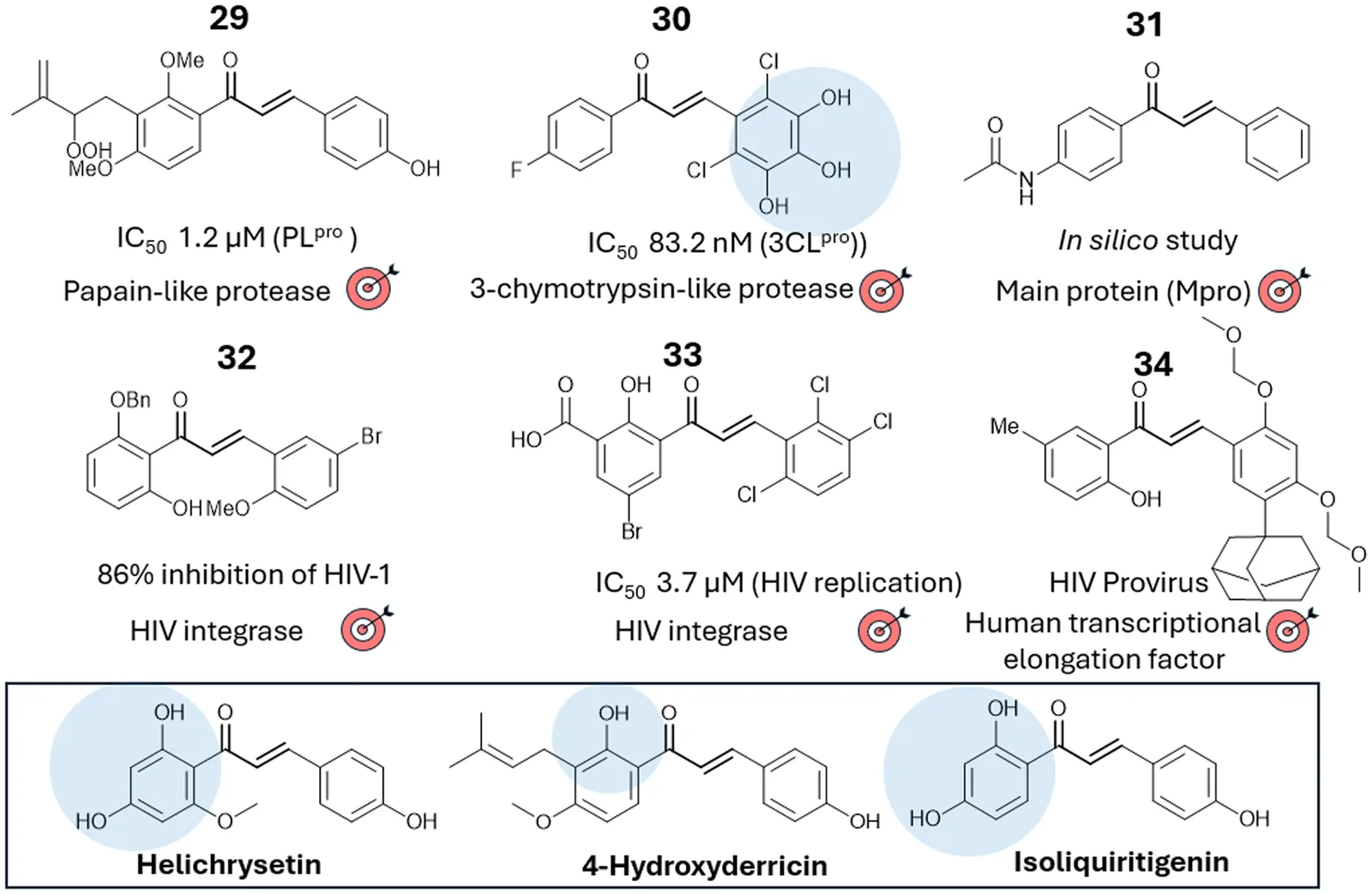
The enone (in bold) within chalcones 29–34 and natural product chalcones helichrysetin, 4-hydroxyderricin and isoliquiritigenin with anti-viral activities (inset).

The history of humanity and viruses is a biological arms race where viral mutations constantly challenge the sophisticated defences of the human immune system. Because viruses often hijack the host's own cellular machinery to reproduce, they represent one of the most difficult drug targets in modern medicine.^71^ One of the first examples of a chalcone developed for the treatment of a coronavirus was reported by Ryu *et al.* in 2015.^72^ Chalcone 29, containing a perhydroxyl group, exhibited potent activity against papain-like protease (PLpro), a viral protease and drug target, with an IC_50_ value 1.2 μM (Fig. 8).

**Fig. 9:**
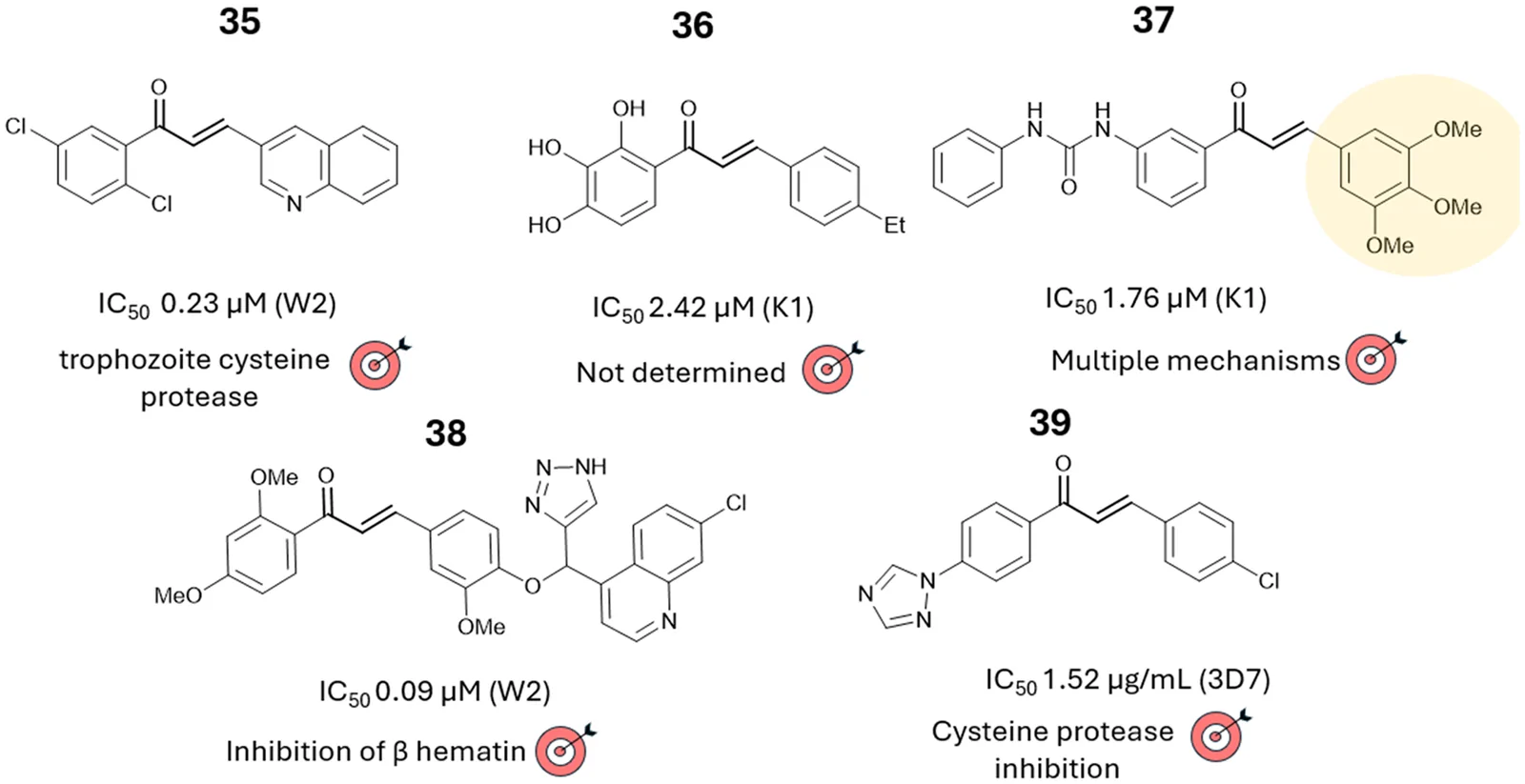
The enone (in bold) within chalcones 35–39.

A study by Kim *et al.* confirmed that the natural product chalcone Helichrysetin (inset Fig. 8) displayed activity against the Middle East respiratory syndrome coronavirus (MERS-CoV).^73^ Two further natural product chalcones are 4-hydroxyderricin, which has activity against Zika virus and isoliquiritigenin with broad spectrum against influenza A, hepatitis C, herpes virus (inset Fig. 8). Silva dos Santos *et al.* performed *in silico* study on six 4-acetamidechalcones with the conclusion that chalcones similar to 31 could bind to the main protein (Mpro) on SARS-CoV-2 with applications to disrupt SPIKE protein interaction and viral replication (Fig. 8).^74^ The development of chalcone-amide as SARS-CoV-2 has recently been discussed as a further avenue to explore.^75,76^ Chalcones targeting HIV have also been explored, for example, 32 displayed 86% inhibition against HIV-1 *via* targeting HIV integrase enzyme (Fig. 8).^77^ Buolamwini *et al.* produced a range of salicylic acid derived chalcones with 33 the most potent inhibitor of HIV with IC_50_ value of 3.7 μM also *via* targeting HIV integrase (Fig. 8).^78^ Xue *et al.* recently reported a chalcone based HIV provirus, 34, which offers an alternative mode of action against HIV (Fig. 8).^79^ This chalcone was confirmed to target transcriptional elongation factor P-TEFb ensuring no viral protein released and preventing HIV infection. The global burden of malaria remains a critical public health emergency, with an estimated 282 million cases and 610 000 deaths reported in 2024.^80^*Plasmodium falciparum* accounts for the majority of severe and fatal infections, with resistance to frontline therapies a growing concern. Chalcones have a pivotal role to meeting this challenge with chalcone 35 discovered from a library of 29 chalcones by Mckerrow *et al.* (Fig. 9)*.*^81^ Chalcone 35 displayed 0.23 μM activity in W2, a chloroquine resistant cell line with molecular modelling suggesting binding to the trophozoite cysteine protease the most likely target. A study of over 100 chalcones by Go *et al.* revealed chalcone 36 with anti-malarial activity of IC_50_ 2.42 μM in K1 a chloroquine resistant cell line (Fig. 9).^82^ The extensive SAR study identified chalcones with hydrophilic character, particularly the 4-hydroxyl-chalcones, were the most active in the series. Rosenthal *et al.* screened 47 chalcones with 37 identified as the most active with an IC_50_ value of 1.76 μM in K1 cells.^83^

Interestingly, the authors suggest this chalcone has multiple mechanism of action. A study of over 46 chalcones by Chibale *et al.* revealed chalcone 38 with potent activity in W2 cells with an IC_50_ of 0.09 μM and inhibition of β-hematin a suggested mode of action.^84^ Chalcone 39 developed by Bhasin *et al.* was one of the first azole based chalcones to be reported with anti-malarial activity (Fig. 9).^85^ In summary, chalcones are easily accessible from a diverse range of commercially available reagents and display a broad range of biological activities across multiple important diseases. Alongside a wealth of therapeutic potential, chalcones also display useful fluorescent properties for the development of biomedical imaging to monitor and diagnose disease progression.

## 6 Chalcones as fluorescent sensors

**Fig. 10:**
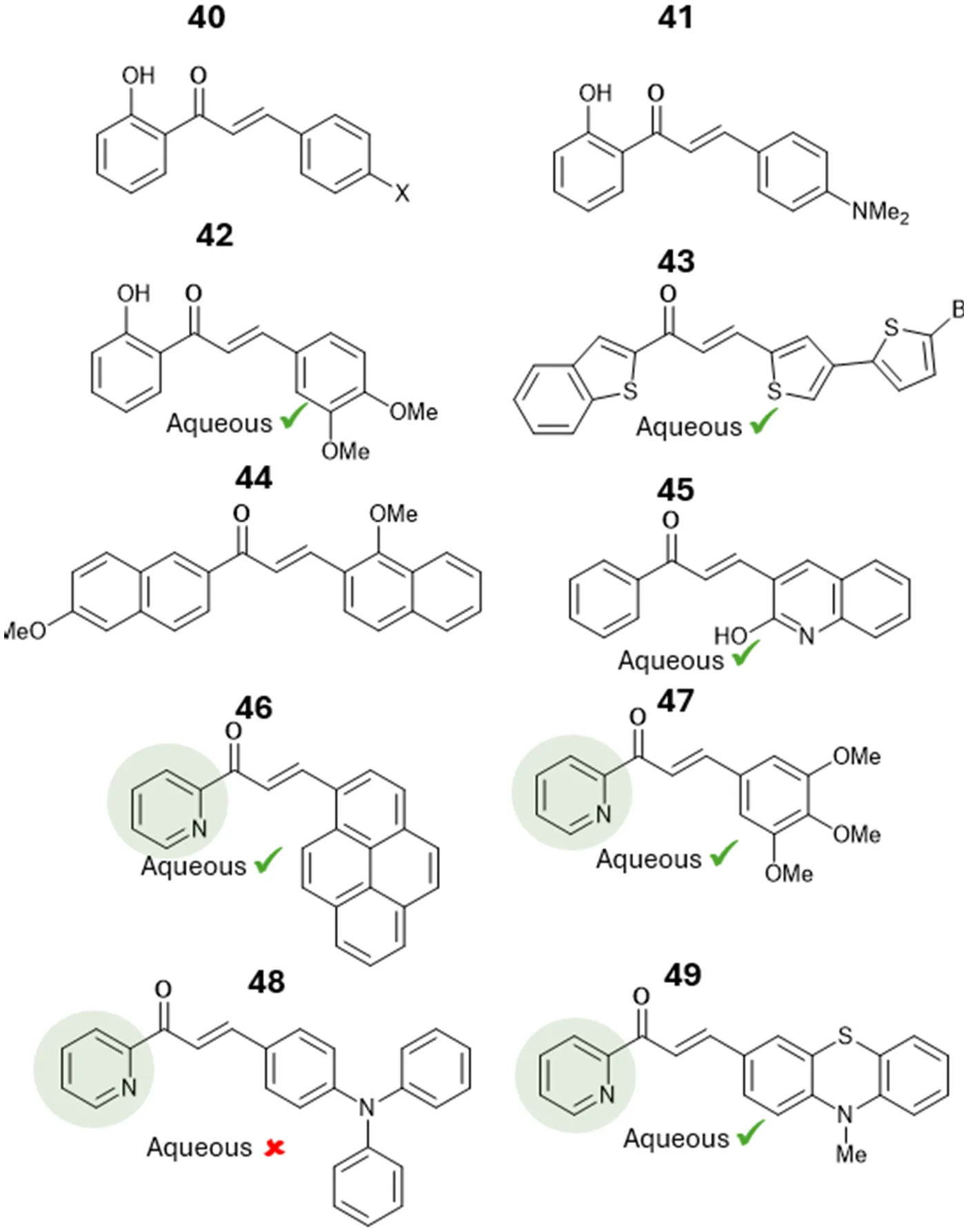
Chalcones 40–49 as fluorescent sensors.

One of the most intensively studied fluorescent chalcones are 2′-hydroxychalcones, for example, simple chalcone 40 (Fig. 10). Chalcone fluorescence arises from extended π-conjugated push–pull architecture involving electron-donor and acceptor substituents around the α,β-unsaturated carbonyl enables intramolecular charge transfer (ICT). In 2′-hydroxy derivatives the phenolic OH engages the carbonyl in an intramolecular hydrogen bond which can also undergo additional fluorescence *via* excited-state intramolecular proton transfer (ESIPT). Seminal photophysical studies by Pang *et al.* on 4-(dimethylamino)-2′-hydroxychalcone 41 established that ICT and ESIPT can coexist within a single scaffold, producing exceptionally large Stokes shifts (>200 nm) in solution and deep red to near-infrared emission in the solid state (Fig. 10).^86^ This is highly attractive for the design of bioimaging sensors. These unusual properties have led to 2′-hydroxychalcones being used as precursors for other fluorescent sensors.^87^ A further 2′-hydroxychalcone, 42, was reported by Abdelaziz *et al.* as a “turn off” sensor for Fe^3+^ in aqueous environments (Fig. 10).^88^

**Fig. 11:**
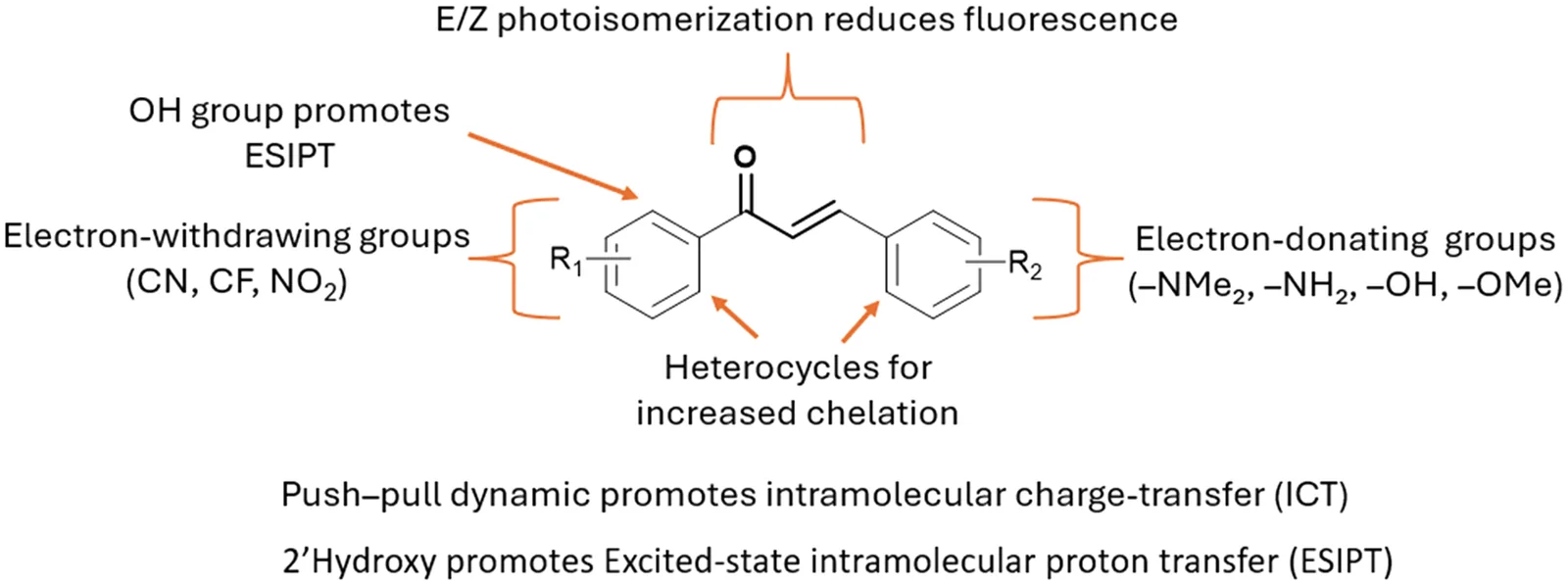
Chalcone fluorescent activity profile.

The introduction of heterocycles has been widely explored for example thiophene^89^ in chalcone 43, naphthalene^90^ in 44, hydroxyquinoline^91^ in 45 and pyrene in 46 (Fig. 10).^92^ The incorporation of a well-established metal chelators, for example pyridine, into the chalcone scaffold is highly advantageous for sensing a variety of analytes with chalcones 47–49 leading examples for Ni^2+^, Fe^3+^, Pd^2+^ and Cu^2+^ respectively (Fig. 10).^93–95^ Singh and Choudhury have further explored chalcone-based derivatives for sensing applications^96^ alongside their incorporation of these fluorescent properties within novel laser applications.^97^ A chalcone fluorescent structural activity relationship highlights key requirements for chalcone fluorescent sensor design (Fig. 11). In summary, chalcones represent a structurally diverse and largely underexplored sensing platform, with approx. 5140 reported structures constituting a fraction of an estimated 680 000 theoretical compounds from commercial reagents. Most of the chemical space remains unexplored providing opportunities for further discovery. The cyclisation of chalcones to pyrazolines, and further oxidation to pyrazoles expands this chemical space from thousands to millions of potential molecules with a multitude of applications.

## 7 Pyrazolines synthesis

**Fig. 12:**
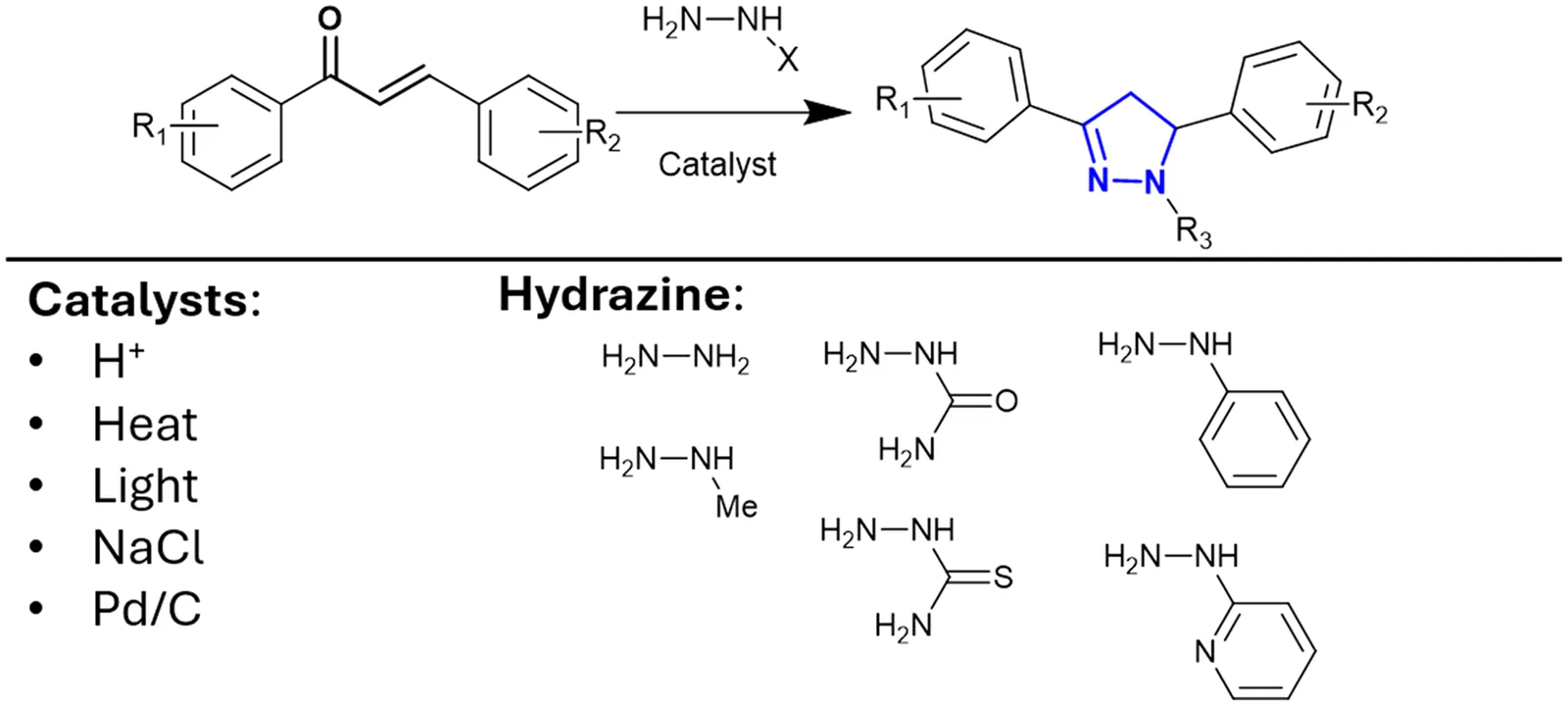
Conversion of chalcones to pyrazolines *via* hydrazines.

While chalcones are highly significant, they are equally valuable as versatile precursors to pyrazolines. The cyclisation from chalcone to pyrazoline is highly adaptable with a range of commercially available and inexpensive hydrazine derivatives (Fig. 12).

A range of conditions promote this transformation, including heat, light, transition metals, and simple salts such as NaCl.^98,99^ The resulting pyrazolines enable precise orientation of substituents around the ring, making them ideally suited to the design of compounds targeting specific biological activities for example, novel anti-cancer agents.

## 8 Pyrazolines as anti-cancer agents

**Fig. 13:**
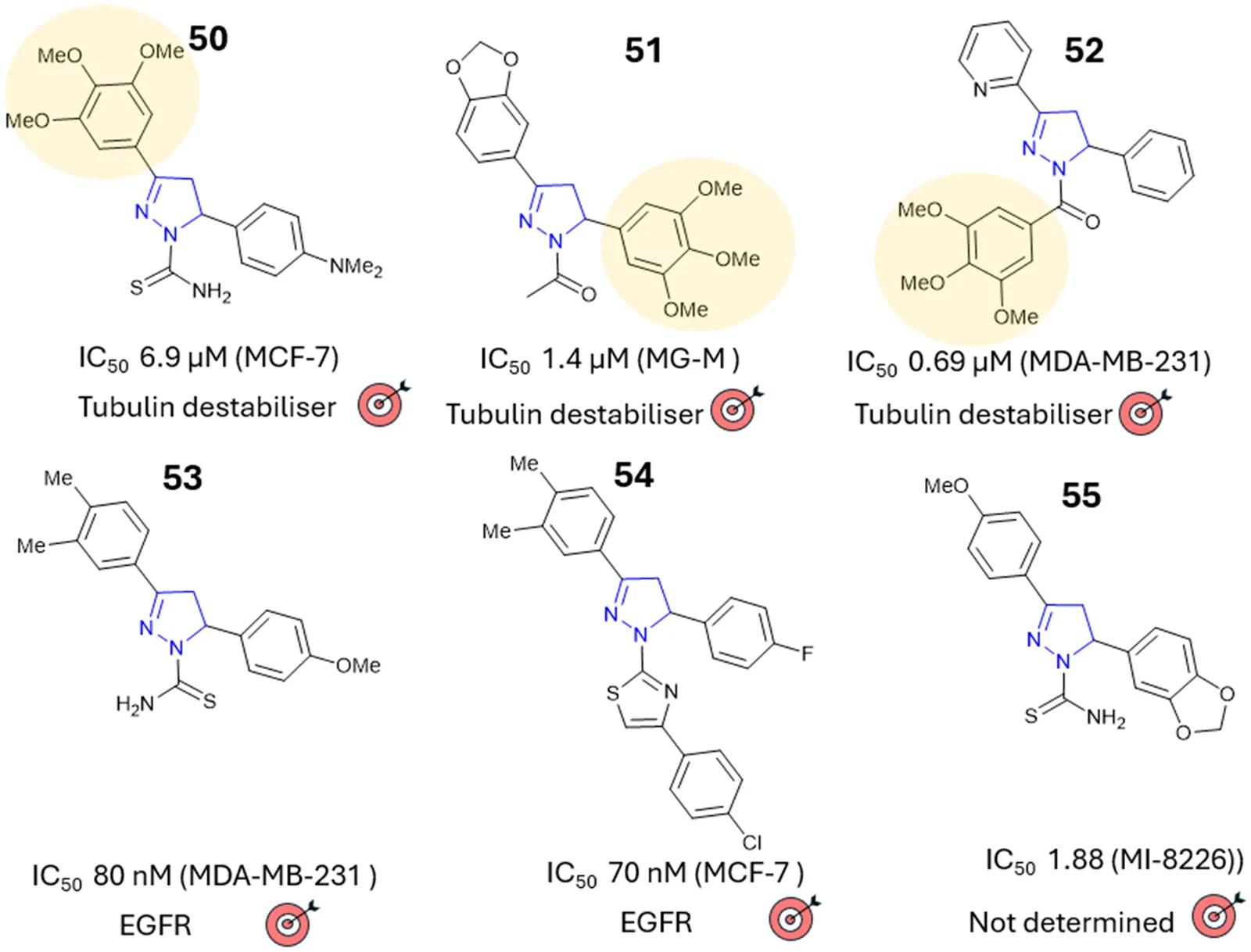
The pyrazoline ring (in blue) within pyrazolines 50–55.

The 3,4,5-trimethoxyl unit was widely explored in the search for anti-cancer chalcones (Fig. 3), conversion to 3,4,5-trimethoxyl pyrazolines retains potent anti-cancer activity exemplified by pyrazolines 50–52 which target tubulin (Fig. 13).^100,101^ The targeting of EGFR using pyrazoline 53–54 (Fig. 13) gave rise to IC_50_ values of 80 nM and 70 nM respectively demonstrating the potential of pyrazolines, synthesised *via* two-step chemistry from commercial reagents, as promising novel anti-cancer treatments.^102,103^

**Fig. 14:**
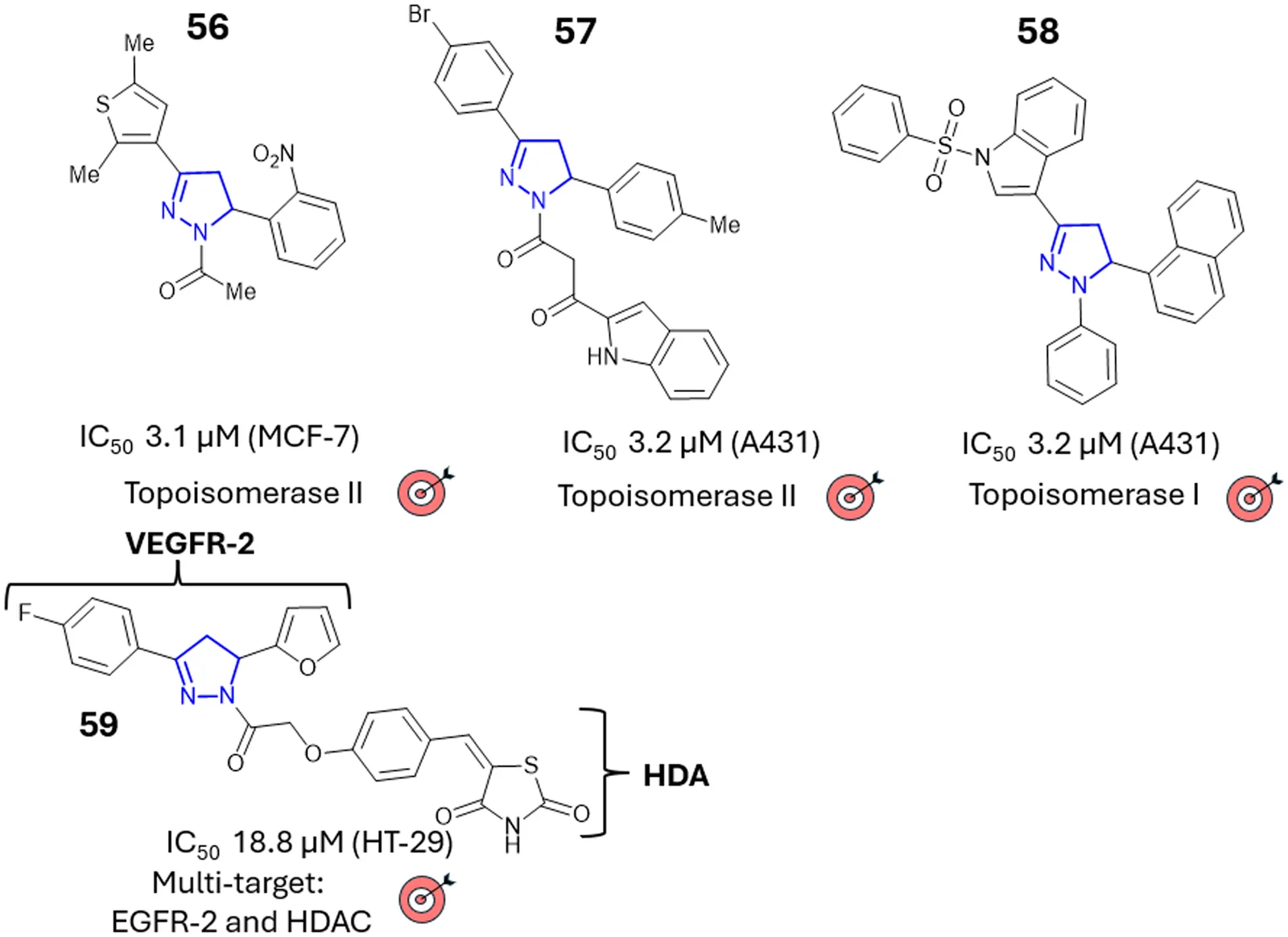
The pyrazoline ring (in blue) within pyrazolines 56–59.

Topoisomerase, the enzyme used during DNA replication is responsible for the anti-cancer activity observed in pyrazolines 56–58 (Fig. 14).^104–106^ A recent development in pyrazolines for cancer was pyrazoline 59 which incorporated the pharmacophore of two drugs, one targeting VEGFR-2 and the other HDA (Fig. 14).^107^ The ability to orientate the three substituents around the pyrazoline ring enable fine-tuning of scaffold design for applications. This is particularly useful in the design of novel pyrazoline based anti-inflammatory treatments.

## 9 Pyrazolines as anti-inflammatory agents

**Fig. 15:**
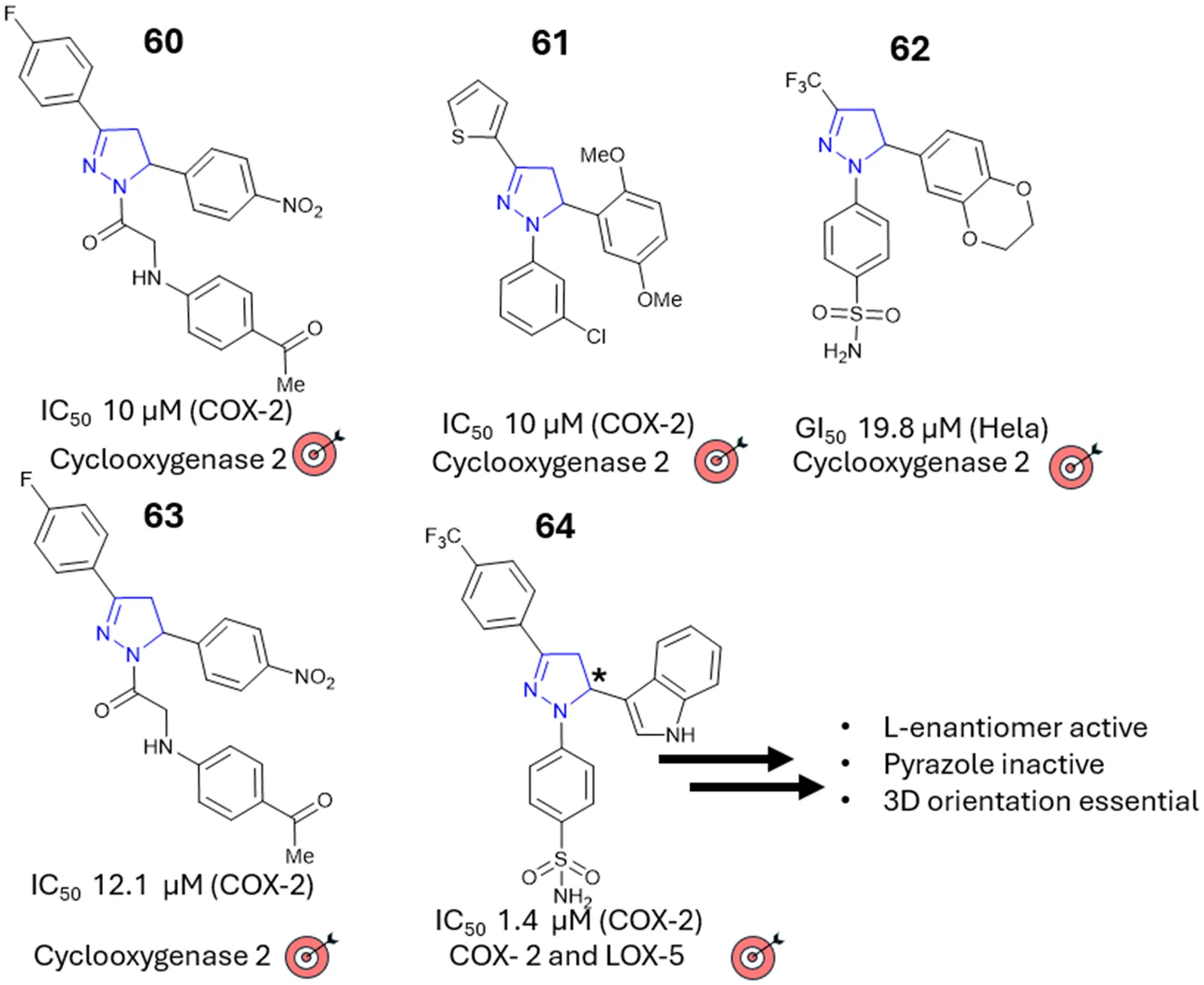
The pyrazoline ring (in blue) within pyrazolines 60–64.

In addition to their anti-cancer properties, pyrazolines have emerged as highly privileged scaffolds for mitigating chronic and acute inflammatory disorders. From a medicinal chemistry perspective, the rigid architecture of the five-membered nitrogen heterocycle, combined with the modular nature of the starting reagents, serves as an ideal hub to orientate three separate R-groups across a defined space. This enables the precise three-dimensional orientation to target specific enzymes and proteins. These derivatives typically exert their therapeutic effects by suppressing the production of pro-inflammatory mediators, interleukins, and specialized enzymes that drive the inflammatory response, for example cyclooxygenase. Pyrazolines 60–63, all derived from chalcone precursor, all display low micromolar activity against COX-2 (Fig. 15).^108–110^ Reddy *et al.* screened twenty indole-based pyrazolines for anti-inflammatory activity against both COX and LOX with the discovery of pyrazoline 64 with IC_50_ value of 1.4 μM against COX-2 and 72-fold selectivity index for COX-2 over COX-1.^110^ This pyrazoline was also active against LOX-5 with 41% inhibition at 10 μM demonstrating dual activity.

Due to the presence of a chiral centre on position five of the pyrazoline ring (* in Fig. 15) chiral HPLC was used to analyse each individual enantiomer. The enantiomer with a positive optical rotation (l-enantiomer) was found to be responsible for the activity observed whereas the d-enantiomer displayed negligible activity. Aromatisation to the pyrazole abolished all biological activity demonstrating that while pyrazolines are inherently racemic often one enantiomer is significantly more active than the other. This has been observed previously highlighting the three-dimensional (3D) orientation of the substituents around the pyrazoline ring is critical to activity.^106,111^ A rigid, well-defined scaffold that orientates functional groups is often a key feature of a privileged scaffold with pyrazolines an outstanding example.

## 10 Pyrazolines as anti-viral agents

**Fig. 16:**
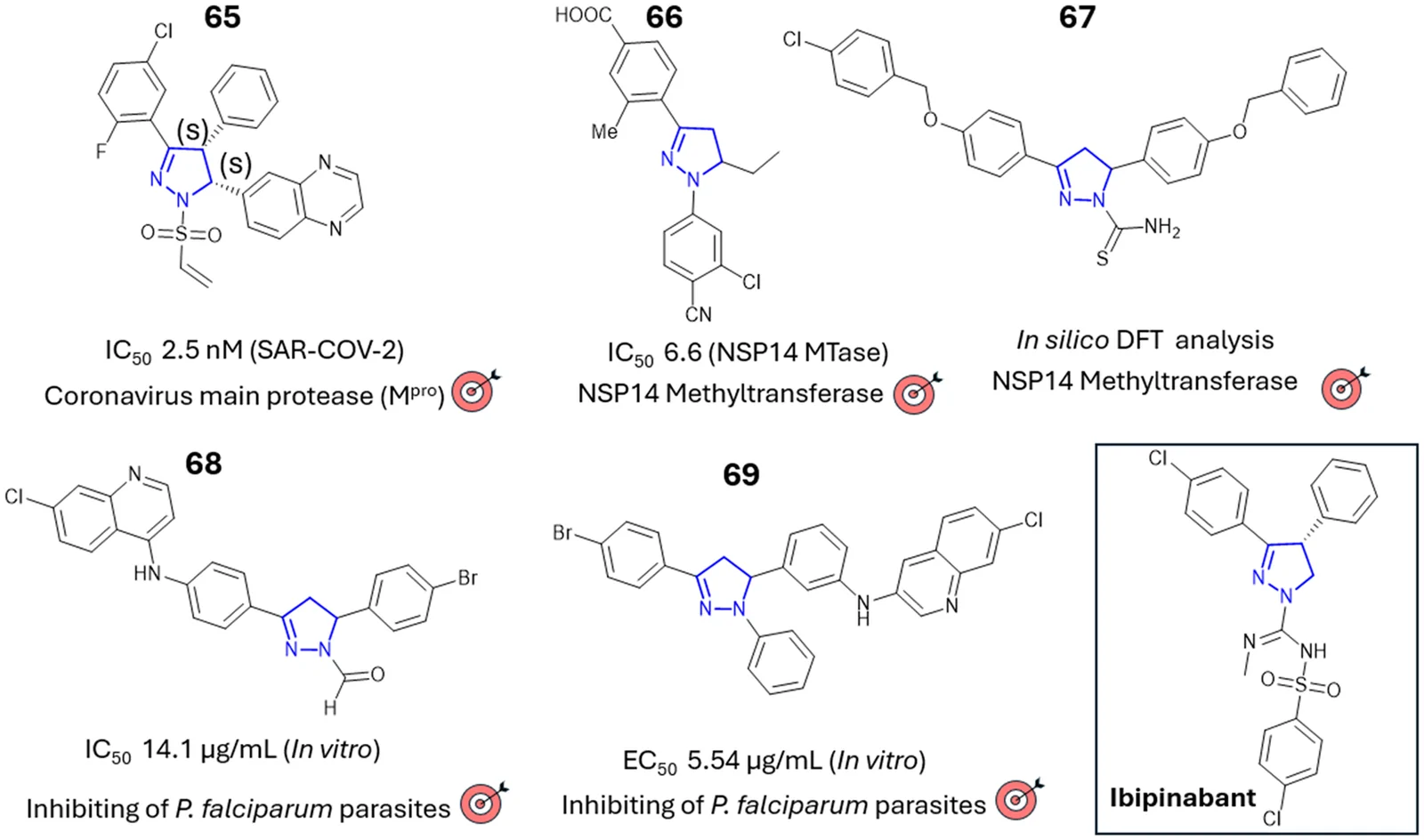
The pyrazoline ring (in blue) within pyrazolines 65–69 and synthetic cannabinoid CB1 receptor antagonist ibipinabant.

Nomura *et al.* screened 21 pyrazolines using a three-step synthetic route *via* commercially available reagents as novel anti-viral agents for COVID-19 and other coronavirus.^112^ A range of different heterocycles were investigated with the lead pyrazoline, 65, containing a quinoline side unit (Fig. 16). The single *S* enantiomer of this pyrazoline was essential for nanomolar activity displaying a broad range of IC_50_ values including a value of 2.5 nM in severe acute respiratory syndrome coronavirus (SARS-CoV-2). Further studies revealed pyrazoline 65 suffers from poor solubility and cell permeability.^112^ This study demonstrates the outstanding potential of pyrazolines to be rapidly synthesized and screened from commercial reagents in the search for novel anti-viral agents. A further exploration of pyrazolines for the use in SARS-CoV-2 was reported by Large *et al.* in which 35 novel pyrazolines were screened and pyrazoline 66 was the first small molecule inhibitor of non-structural protein methyltransferase (NSP14 MTase) with anti-viral activity (Fig. 16).^113^ The pyrazoline scaffold is particularly useful as a framework for *in silico* modelling for novel agents for SARS-CoV-2, for example pyrazoline 67 (Fig. 16).^114^

**Fig. 17:**
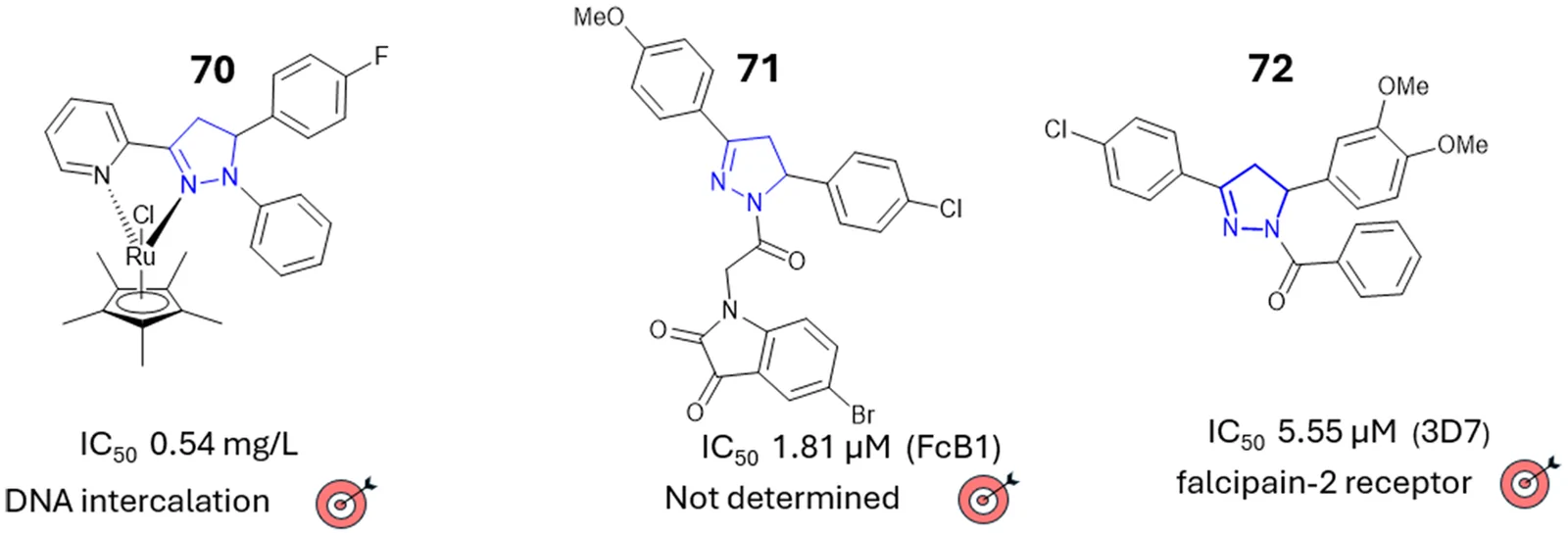
The pyrazoline ring (in blue) within pyrazolines 70–72.

The *in silico* identification of lead compounds, synthesis, testing and confirmation of these leads is emerging as a rapid method to increase drug development efficiency.^115–117^ The use of quinoline-based chalcones is well-established and the use of such precursors transfers biological activity to the associated pyrazolines. The search for novel anti-malarial treatments is continuing with pyrazolines containing quinoline a common approach. Cobo *et al.* reported eighteen quinoline pyrazolines with 68 displaying 58% inhibition of *Plasmodium falciparum* (Fig. 16).^118^ Relocating the quinoline to the position five of the pyrazoline ring retains anti-malarial activity, for example pyrazoline 69 (Fig. 16) reported by Insuasty *et al.*^119^ Patel *et al.* reported a series of pyrazoline based half-sandwich Ru^3+^ complexes with anti-malarial activity due to DNA intercalation (pyrazoline 70 in Fig. 17).^120^ A further study by Patel included pyrazolines with a thiophene with similar anti-malarial activity.^121^ Lesyk *et al.* reported a series of thiazolidinone based pyrazolines with anti-malarial activity with pyrazoline 71 the lead compound (Fig. 17).^122^ Wahyuningsih *et al.* reported pyrazoline 72 with anti-malarial activity due to binding to the facipain-2 receptor (Fig. 17).^123^ In summary pyrazolines are on the front line in the development of new anti-viral treatments for serious health concerns such as coronavirus and malaria.

## 11 Pyrazolines as fluorescent sensors

**Fig. 18:**
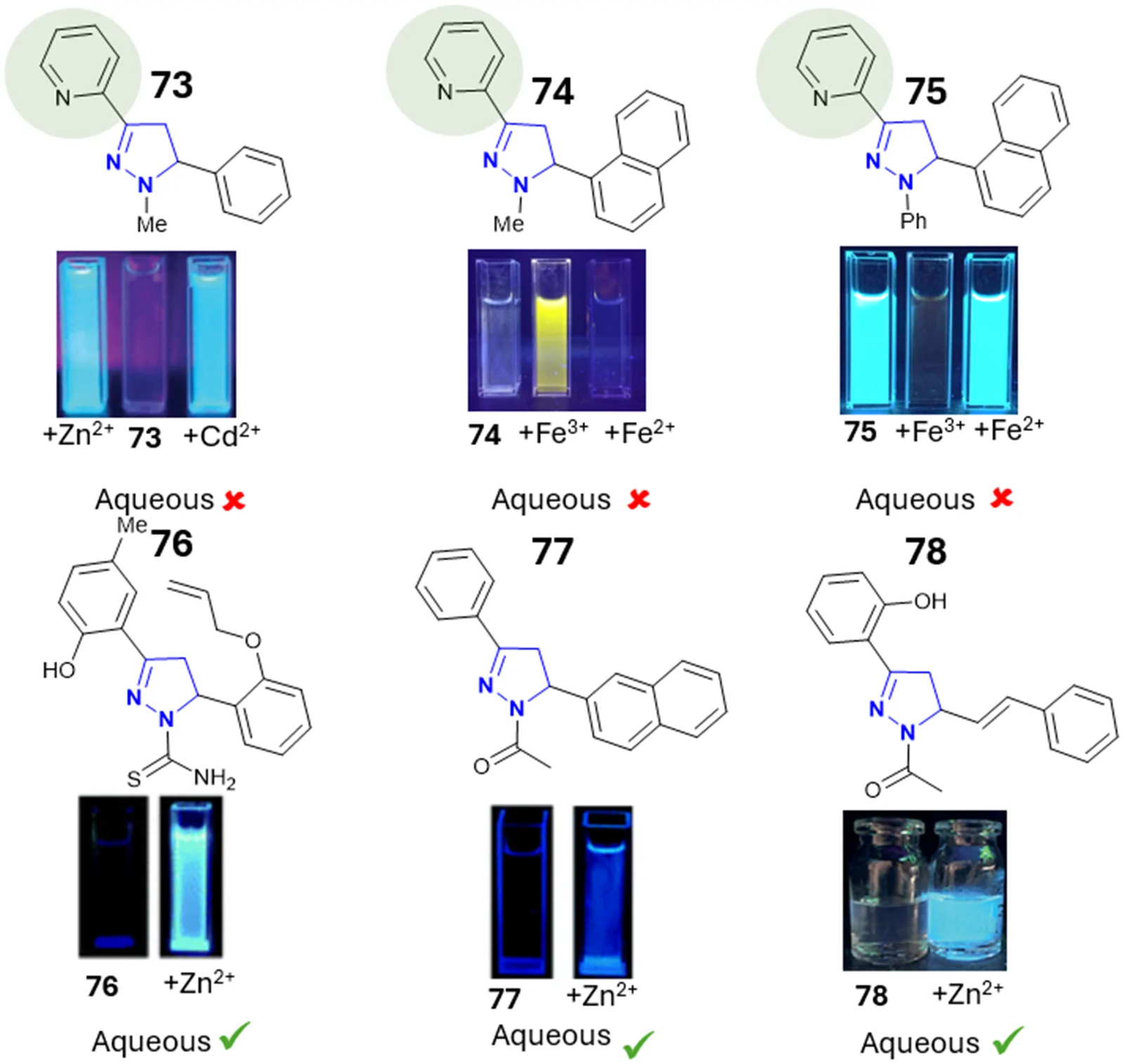
The pyrazoline scaffold (in blue) in sensors 73–78, reproduced from ref. 124–128 with permission from RSC^124–128^ copyright 2024, 2012, 2014 and 2018 respectively.

The use of the pyrazolines in the understanding of disease diagnosis and progression is a parallel theme of active research. The pyrazoline scaffold occupies a unique position among fluorescent heterocycles as a five-membered, non-aromatic ring system bearing two adjacent nitrogen atoms, gives rise to strong, tuneable fluorescence *via* photoinduced electron transfer (PET). The careful selection of chalcone and hydrazine precursors enables rapid synthesis and modular design, making pyrazolines highly amenable to systematic screening for fluorescent sensing properties. The selective detection of metal ions directly implicated in human disease for example Zn^2+^ dysregulation in neurodegeneration and diabetes, positions pyrazoline-based sensors as practical tools for monitoring disease onset and progression in biological environments. This is demonstrated by one of the simplest pyrazoline structures, pyrazoline 73 (Fig. 18), bearing a methyl group at position one and a single pyridine unit at position three of the pyrazoline ring (in blue, Fig. 18).^124^

This sensor was a highly selective “turn on” sensor for the detection of Zn^2+^ and Cd^2+^ in MeCN. The related sensor, pyrazoline 74, with a naphthyl unit was revealed to be a selective “turn on” sensor for Fe^3+^/Fe^2+^ also in MeCN (Fig. 18).^125^ Substitution of the pyrazoline methyl for a phenyl in pyrazoline 75 reversed the response from “turn on” to “turn off” for Fe^3+^ highlighting how minor modifications around the pyrazoline heterocycle, can profoundly influence photophysical properties.^125^ Pyrazoline 73–75 only operate in pure organic solvents limiting real world applications, however pyrazolines 76–78 (Fig. 18) were active in aqueous environments with selective detection of Zn^2+^*in vitro*.^126–128^ This demonstrates that simple pyrazolines can be a valuable resource for the monitoring of Zn^2+^ in disease progression.

**Fig. 19:**
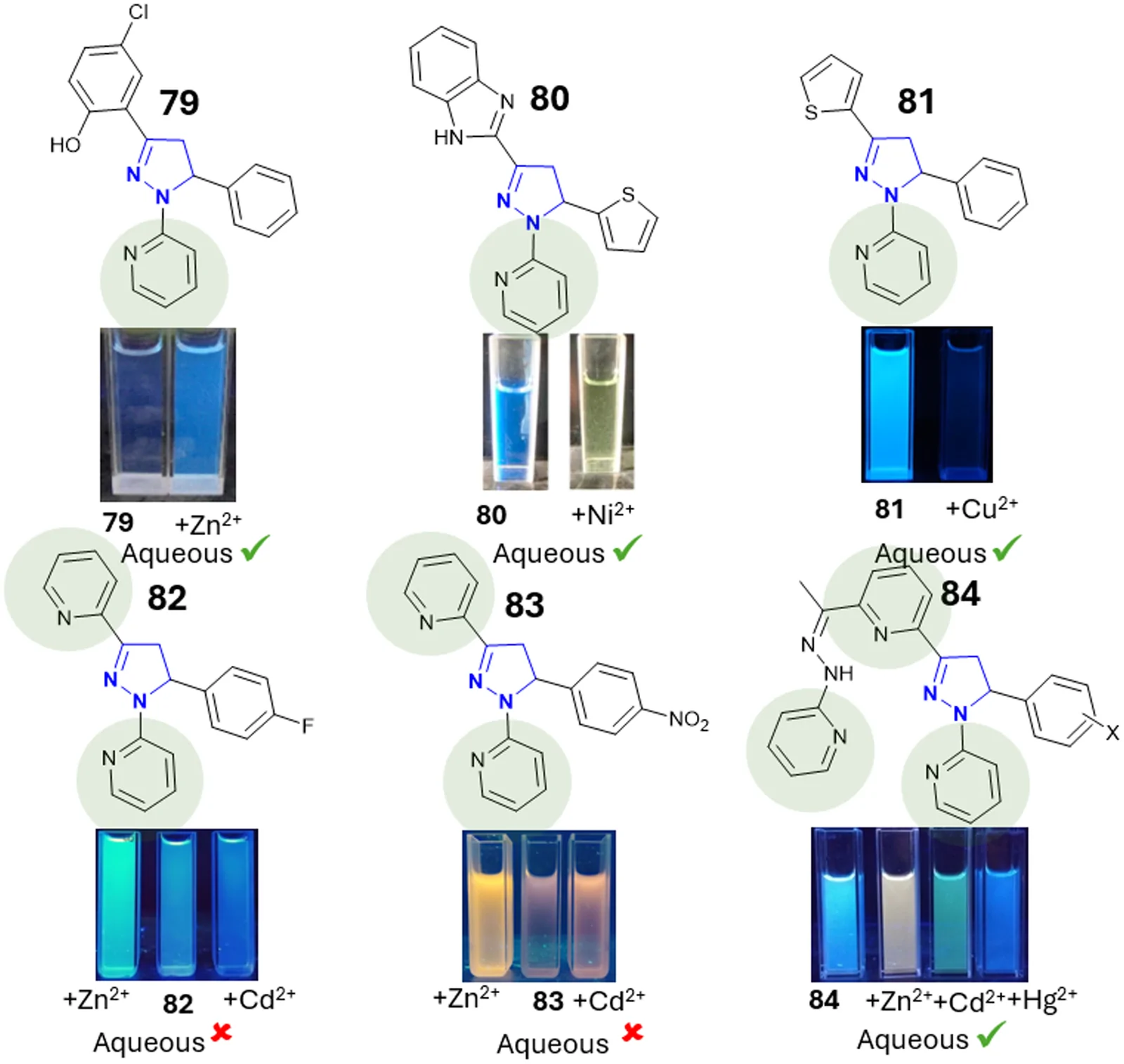
The pyrazoline scaffold (in blue) in sensors 79–84, reproduced from ref. 128 and 132 with permission from RSC^128,132^ copyright 2018 and 2026 respectively, Elsevier^129,130^ copyright 2011, 2017 respectively and Springer Nature^131^ copyright 2013.

Interest in pyrazolines continued with the incorporation of pyridine units at position one of the pyrazoline, yielding sensors 79–81 (Fig. 19) which selectively detect Zn^2+^, Ni^2+^, and Cu^2+^*in vitro* respectively.^128–130^ A high-throughput screening (HTS) study revealed pyrazoline 82 with a slight preference for Zn^2+^/Cd^2+^ however, substitution of the 4-F on the phenyl for a 4-NO_2_ profoundly increased Stokes shift in pyrazoline 83.^132^ This demonstrates fine-tuning of photophysical response *via* minor structural modifications. Hydrazone–pyrazoline sensors, 84 which detects Zn^2+^, Cd^2+^, and Hg^2+^ at separate wavelengths in aqueous environments were reported (Fig. 19).^133^ Further studies revealed a series of analogues (X: 4-OMe, 3,4-OMe and 4-F) were well-tolerated with the ability to detect these three group 12 in rural areas using a portable device without the need for expensive instrumentation or mains electricity (Fig. 19). The sensing versatility of pyrazolines extends well beyond simple ion detection: the scaffold has proven uniquely well-suited to molecular logic gates.^134^ This was originally pioneered by de Silva *et al.* and continued by Magri *et al.* extending to “lab-on-a molecule” devices.^135^ In summary, pyrazolines are a highly versatile privileged structure with a diverse range of medicinal, sensing, and molecular logic properties. Orientation of the three substituents, combined with the high modularity of the commercially available units offers significant advantages. The examples highlighted demonstrate that structural complexity is not a prerequisite for complex functionality; simple molecules can display highly useful behaviours. Advances in digital discovery tools and automated synthesis platforms are poised to accelerate this process of pyrazoline development further still.

## 12 Pyrazoles synthesis

**Fig. 20:**
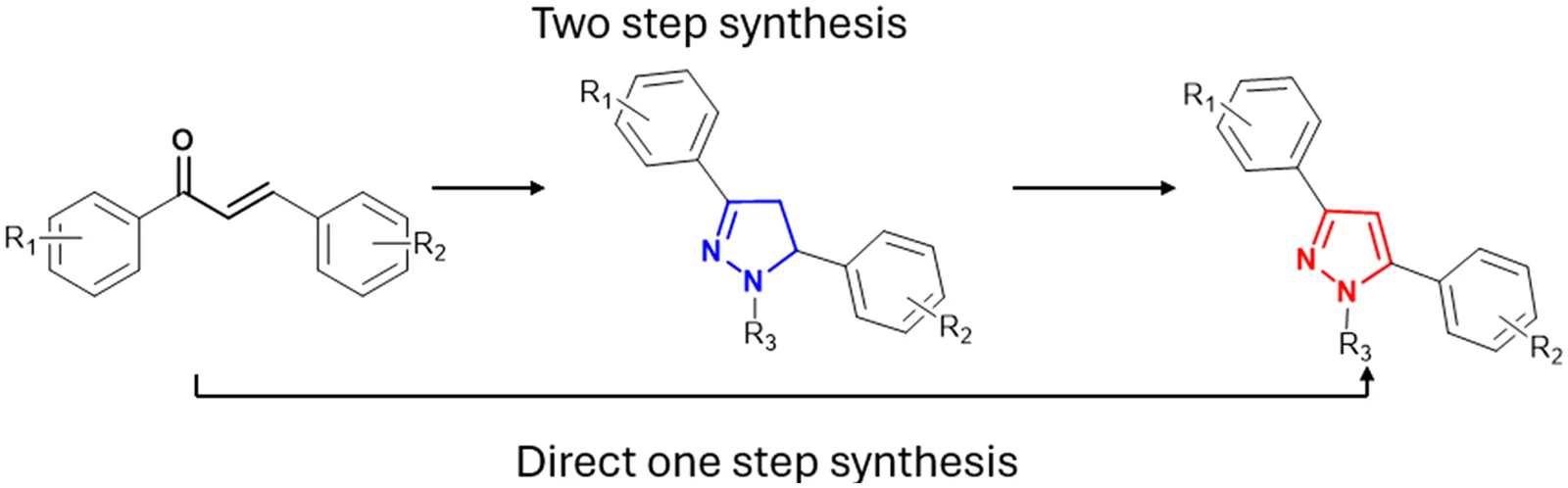
Pyrazole synthesis.

Pyrazole, an aromatic five-membered heterocycle, is a prominent privileged scaffold present in a diverse range of biologically active compounds^136^ and approved drugs.^137^ Pyrazole can easily be synthesised from pyrazolines *via* oxidation^138^ or directly from chalcones in a single synthetic transformation (Fig. 20).^139^

## 13 Pyrazoles as anti-cancer agents

**Fig. 21:**
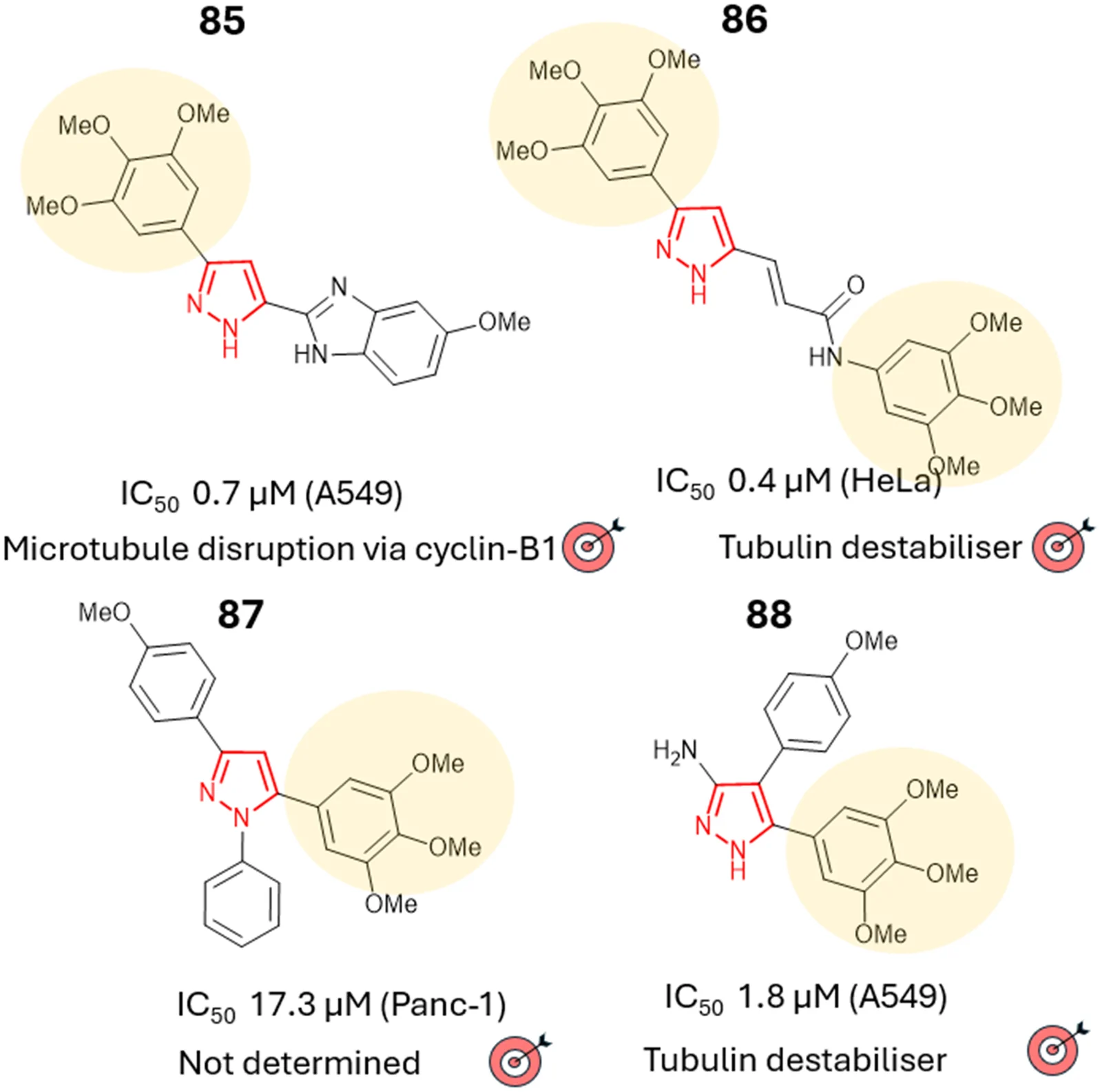
Pyrazole 85–88 with anti-cancer activities.

**Fig. 22:**
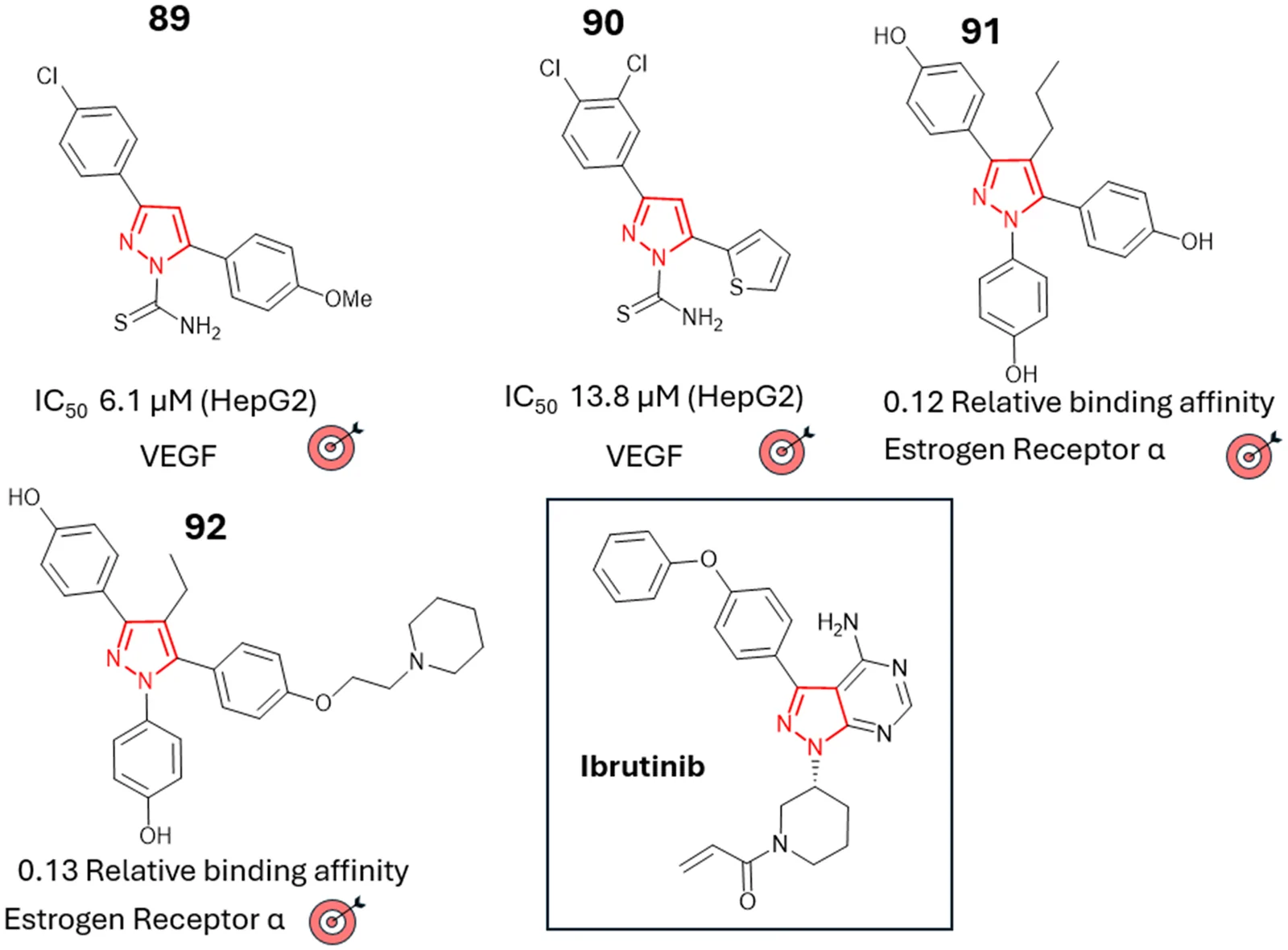
Pyrazole 89–92 and Ibrutinib with anti-cancer activities.

The prevalence of the 3,4,5-trimethoxy motif in chalcone and pyrazolines is also present in pyrazole with this scaffold responsible for the biological activity observed in pyrazole 85 (Fig. 21).^140^ This pyrazole has a IC_50_ value of 0.7 μM due to the disruption of tubulin. A pyrazole bearing two such units, pyrazole 86 is also a tubulin destabiliser with IC_50_ value in HeLa cells of 0.4 μM (Fig. 21).^141^ A similar trend is observed with pyrazole 87 and 88 (Fig. 21).^142,143^ A rapidly emerging target for pyrazole based anti-cancer treatments are VEGFR-2 kinase inhibitors^144^ for example pyrazole 89 and 90 which target VEGF with IC_50_ values in a range of cancer cell lines ranging from 6.1 to 13.8 μM respectively (Fig. 22).^145,146^

Pyrazoles are also potent compounds for the treatment of breast cancer by targeting the estrogen receptor α for example pyrazole 91 and 92 (Fig. 22).^147,148^ The pyrazole core is central to the mode of activity of these compounds and the clinically approved anti-cancer treatment Ibrutinib for lymphocytic leukaemia (inset Fig. 22).

## 14 Pyrazole as anti-inflammatory agents

**Fig. 23:**
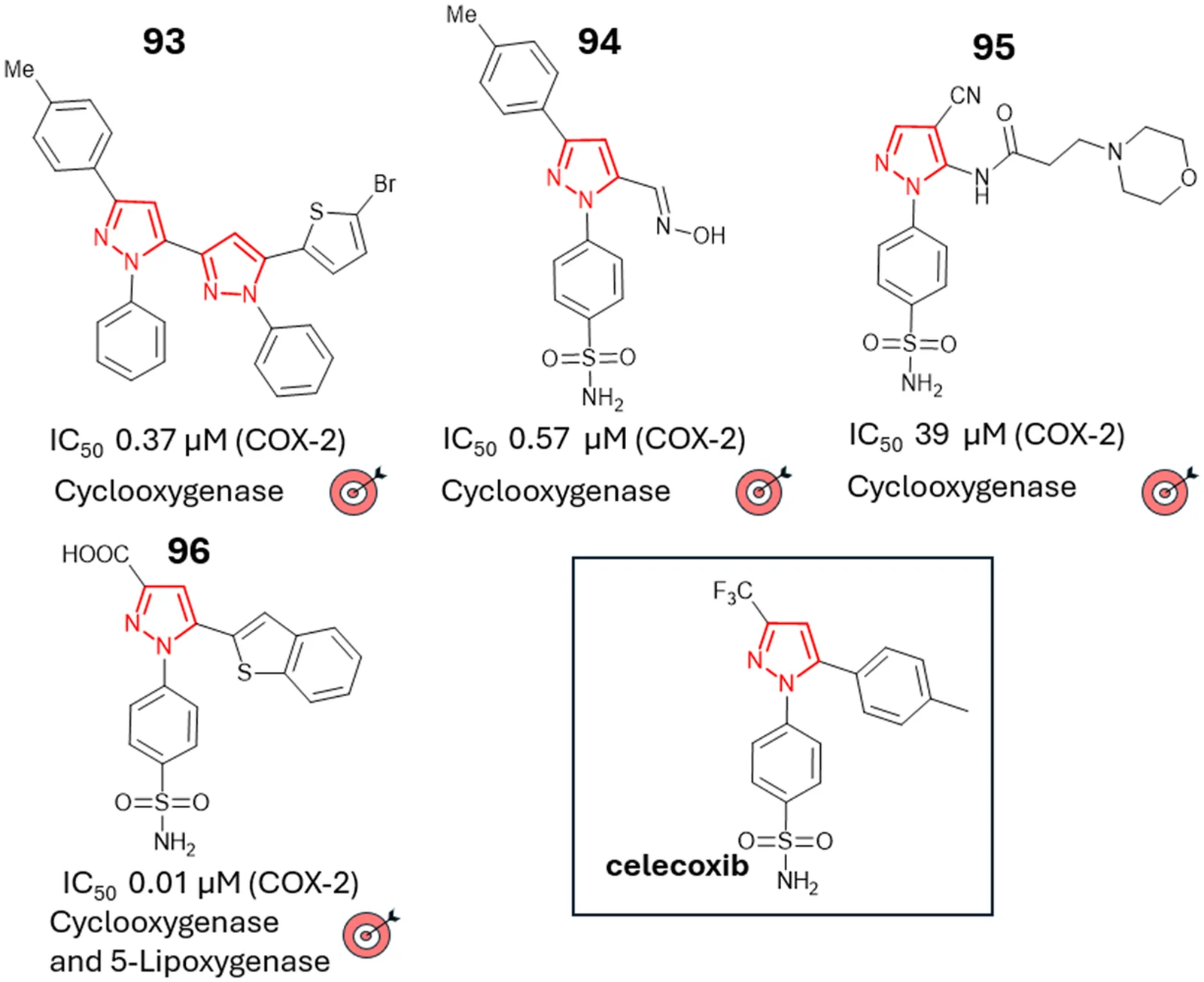
Pyrazole 93–96 with anti-inflammatory activities with approved anti-inflammatory drug celecoxib.

Pyrazoles display a range of anti-inflammatory activities, for example pyrazole 93 displayed an IC_50_ value of 0.37 μM for COX-2 (Fig. 23).^149^ This compound, containing two pyrazole rings, has a COX-2 to COX-1 selectivity ratio of >270 which is comparable to the clinically approved drug Celecoxib which has a COX-2 IC_50_ value <0.3 μM and a COX-2 selectivity ratio of 333 (inset Fig. 23). *In vivo* studies with pyrazole 94 confirmed this compound displayed very promising anti-inflammatory activities when compared to celecoxib (Fig. 23).^150^ A similar study confirmed pyrazole 95 as a COX-2 inhibitor (Fig. 23).^151^

A dual COX-2 and 5-LOX inhibitor, pyrazole 96, was reported by Kerdawy *et al.* with IC_50_ values of 0.01 and 17.8 μM respectively (Fig. 23).^152^ In summary, pyrazoles are a promising scaffold for the design of new anti-inflammatory agents^153^ with a well-established history of compounds reporting properties on par with clinically approved anti-inflammatory treatments, for example celecoxib.

## 15 Pyrazole as anti-malarial agents

**Fig. 24:**
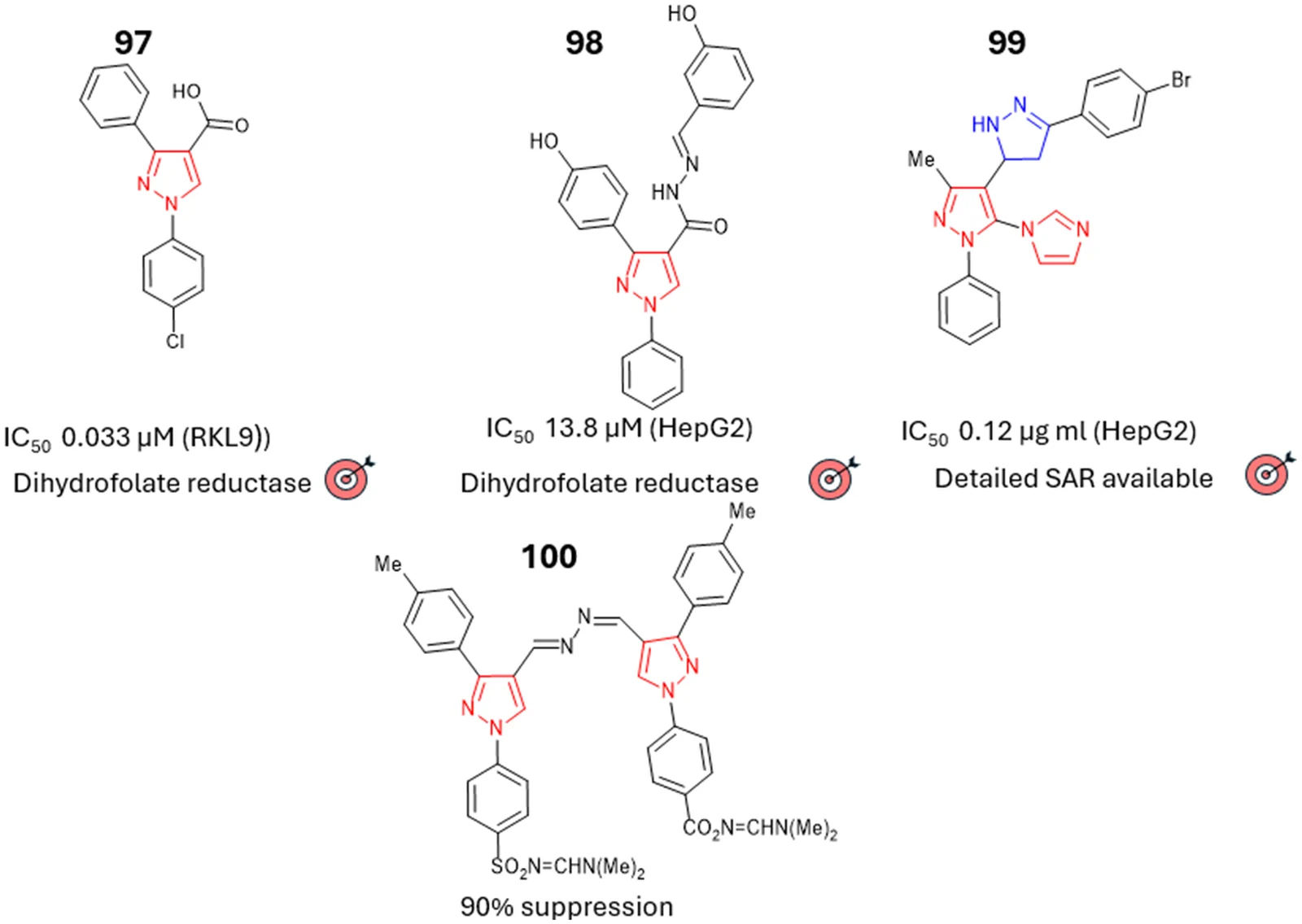
Pyrazole 97–100 with anti-malarial activities.

Pyrazoles are a scaffold for the treatment of malaria with the Bekhit group reporting pyrazole 97 displaying an IC_50_ value of 0.033 μM in the chloroquine resistant strain of *Plasmodium falciparum* (Fig. 24).^154^ Molecular docking studies confirmed the importance of the carboxylic acid for binding dihydrofolate reductase enzyme. A further study also by the Bekhit group reported a superior pyrazole, 98, with an IC_50_ value of 0.0143 μM also in the chloroquine resistant strain of *Plasmodium falciparum* (Fig. 24).^155^ This is significantly more active than the chloroquine phosphate control (IC_50_ 0.193 μM) with detailed *in silico* experiments determining binding to dihydrofolate reductase thymidylate synthase responsible for the activity observed. Kalaria *et al.* reported a mixed pyrazoline and pyrazole compound 99 with a range of anti-malarial, antioxidant and even antituberculosis activity (Fig. 24).^156^ A detailed SAR across 24 compounds revealed that electron-withdrawing groups, particularly bromine, enhanced antituberculosis and antimalarial activity, while fluorine boosted antibacterial and antioxidant potency. This highlights the potential of the pyrazoline and pyrazole pipeline for delivering structurally diverse hits across multiple biological assays. Pyrazole 100 (Fig. 24) exemplifies the potential of hydrazine-coupled dual pyrazole derivatives, demonstrating 90% *in vivo* suppression of *Plasmodium berghei* in infected mice nearly approaching the 100% suppression achieved by chloroquine phosphate.^157^ This demonstrates the promise of this scaffold for the development of novel, multicomponent treatments.

## 16 Pyrazoles as fluorescent sensors

**Fig. 25:**
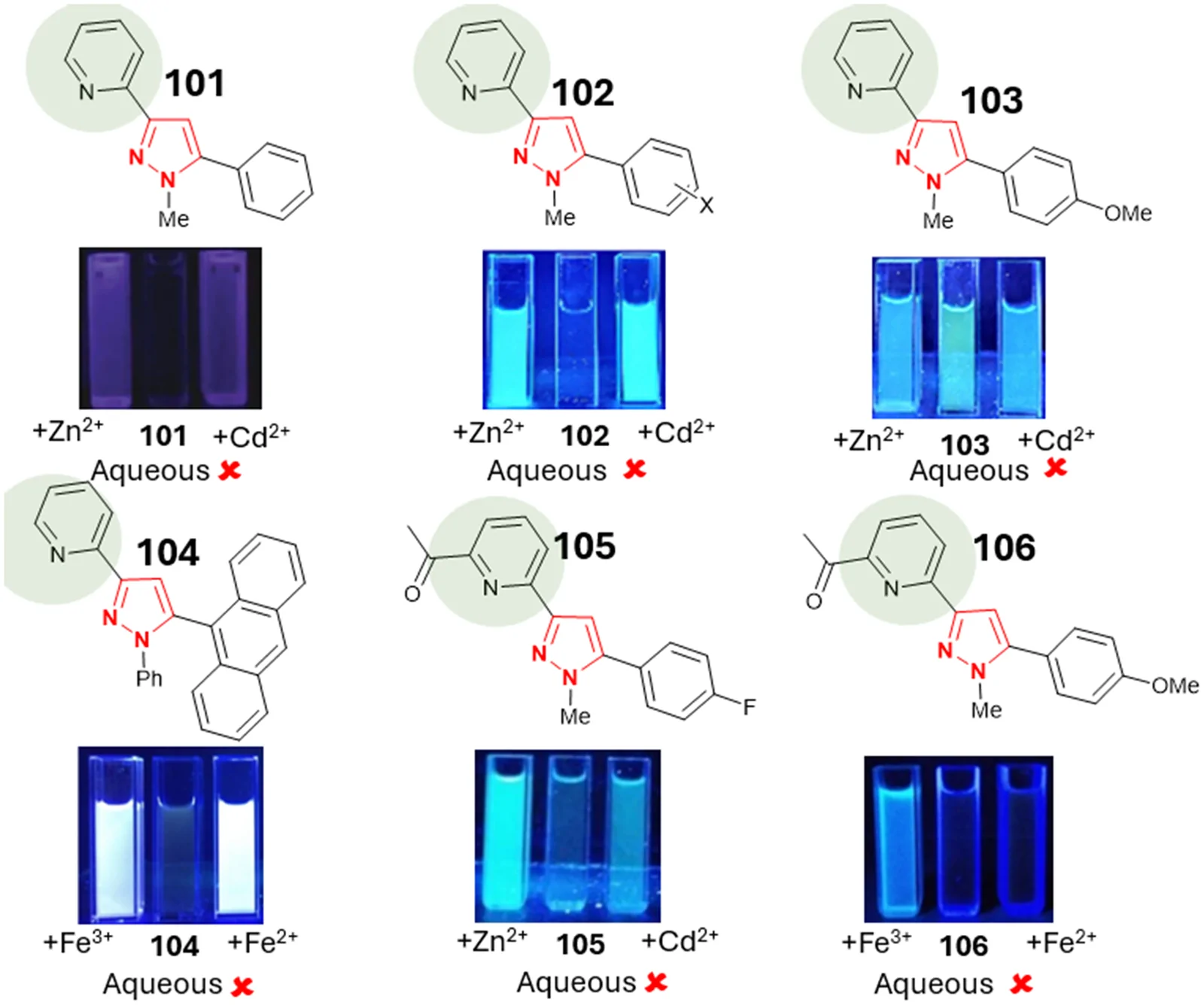
Pyrazole sensors 101–106 in organic solvent, reproduced from ref. 124, 159 and 160 with permission from RSC^124,159,160^ copyright 2012 and 2024 respectively.

**Fig. 26:**
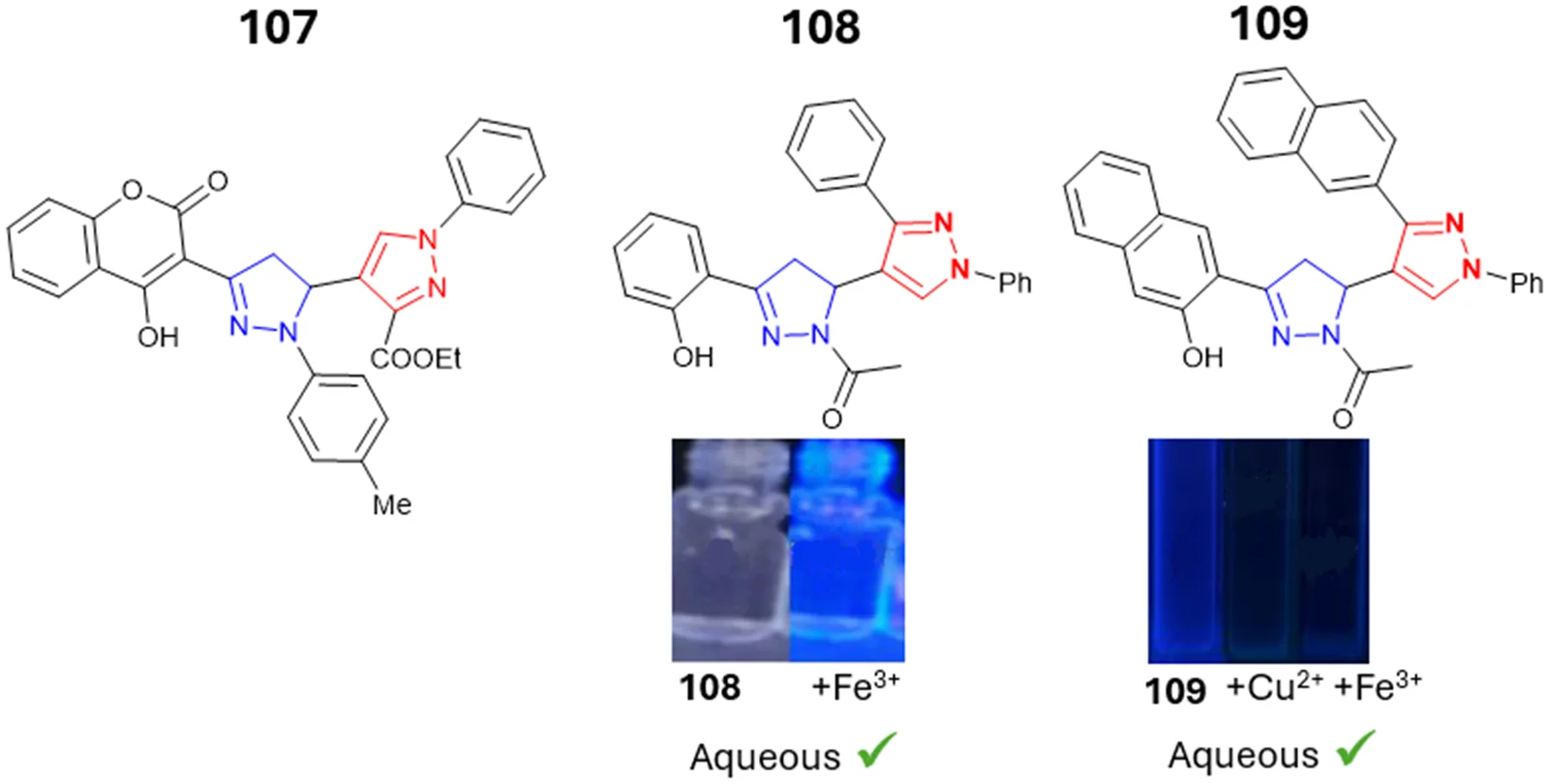
Pyrazole sensors 107–109 in aqueous environments, reproduced from ref. 162 with permission from Springer Nature^162^ copyright 2021 and Elsevier^163^ copyright 2025 respectively.

The use of pyrazole as bioimaging probes is well-established^158^ however pyrazolines typically operate *via* PET, pyrazoles use chelation-enhanced fluorescence (CHEF) or chelation-enhanced quenching (CHEQ). This is due to the aromatic nature of the pyrazole compared to the non-aromatic pyrazoline ring. For example, simple pyrazole 101 displayed minor CHEF in the presence of Zn^2+^ and Cd^2+^ only (Fig. 25).^124^ Further studies revealed that the addition of electron-withdrawing groups on the aryl ring increased CHEF in pyrazole 102 whereas the addition of electron-donating groups switched the response from “turn on” to “turn off” in pyrazole 103 (Fig. 25).^159^ The fluorescence emission wavelength can be tuned by addition of anthracene in pyrazole 104 (Fig. 25).^160^ This is analogous to the pyrazoline sensors 73–75 (Fig. 18) in which minor modifications can be used to fine-tune photophysical effects. Additional studies revealed that the addition of an acetyl side chain to pyrazole 105 increases selectivity for Zn^2+^/Cd^2+^ (Fig. 25).^160^ Substitution of the electron-withdrawing 4-F in pyrazole 105 with an electron-donating 4-OMe in pyrazole 106 switched the sensor to a “turn on” sensor for Fe^3+^/Fe^2+^(Fig. 25).^160^ Fe^3+^ is paramagnetic and typically quenches fluorescence; therefore, the discovery of a “turn on” sensor for Fe^3+^ is of note. One major drawback is these sensors only operate in organic environments, the fluorescent response in aqueous environments is essential if these sensors are to find real world applications. Three excellent examples of pyrazole based fluorescent sensors which operate in aqueous environments are pyrazole 107–109 which can detect Fe^3+^ and Cu^2+^ in aqueous environments including *in vitro* cell cultures (Fig. 26).^161–163^

## 17 Hybridization

The previous sections have examined how each scaffold, chalcone, pyrazoline, and pyrazole, possess distinct properties exploited in the pursuit of novel therapeutics and sensors. We now consider how the merging of two or more of these scaffolds into a single molecular framework, referred to as hybridisation, is unlocking further potential beyond what any individual scaffold can offer. Currently, hybrid scaffolds are designed through iterative, human intuition-led design by prior SAR knowledge. The convergence of machine learning and high-throughput screening (HTS) offers a transformative alternative, enabling thousands of existing compounds to be evaluated across multiple biological panels in an automated manner. This generates the large, consistent datasets required to train predictive models.^164^ These models can identify hybrid structures occupying regions of chemical space that human intuition would not prioritise, with the resulting candidates synthesised, screened, and fed back to iteratively refine the model. This expands the current estimate of 7.5 million compounds within this chemical space of this pipeline by severalfold.

**Fig. 27:**
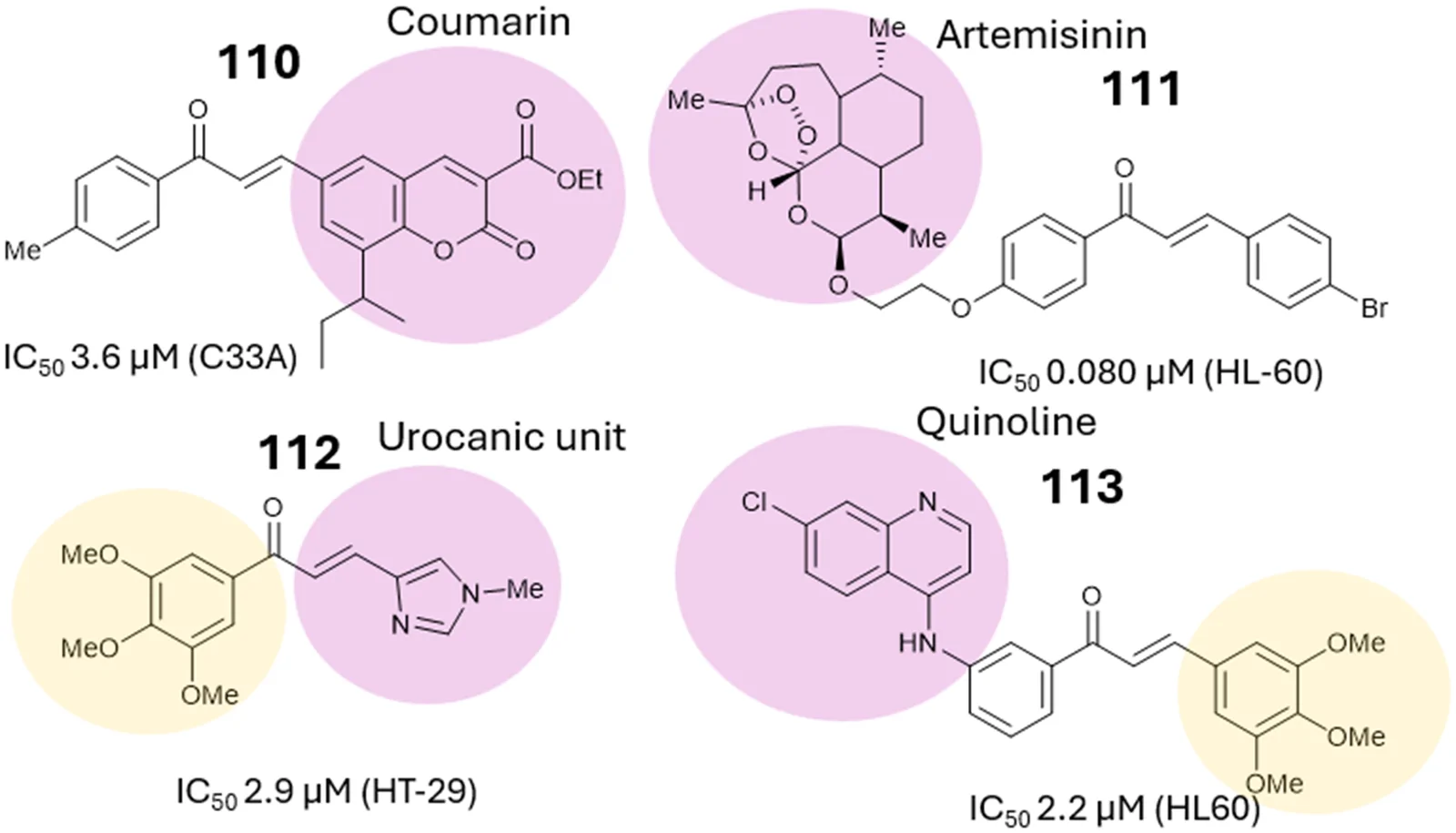
Chalcones 110–113 hybrids combining multiple pharmacophores in a single structure.

It would be impossible to generate these compounds by hand, however by careful selection of suitable candidates this chemical space can be mapped. Below we provide examples of hybridisation to demonstrate this approach. The chalcone scaffold is particularly attractive for hybridisation,^165^ for example chalcones 110–113 combine the pharmacophores coumarin, artemisinin and urocanic unit and quinone present in various natural products (purple highlight in Fig. 27).^166–169^ These chalcones could then be transformed into pyrazolines and their subsequent pyrazoles demonstrating the potential of this pipeline for novel discovery.

## 18 Structural activity relationships (SAR)

**Fig. 28:**
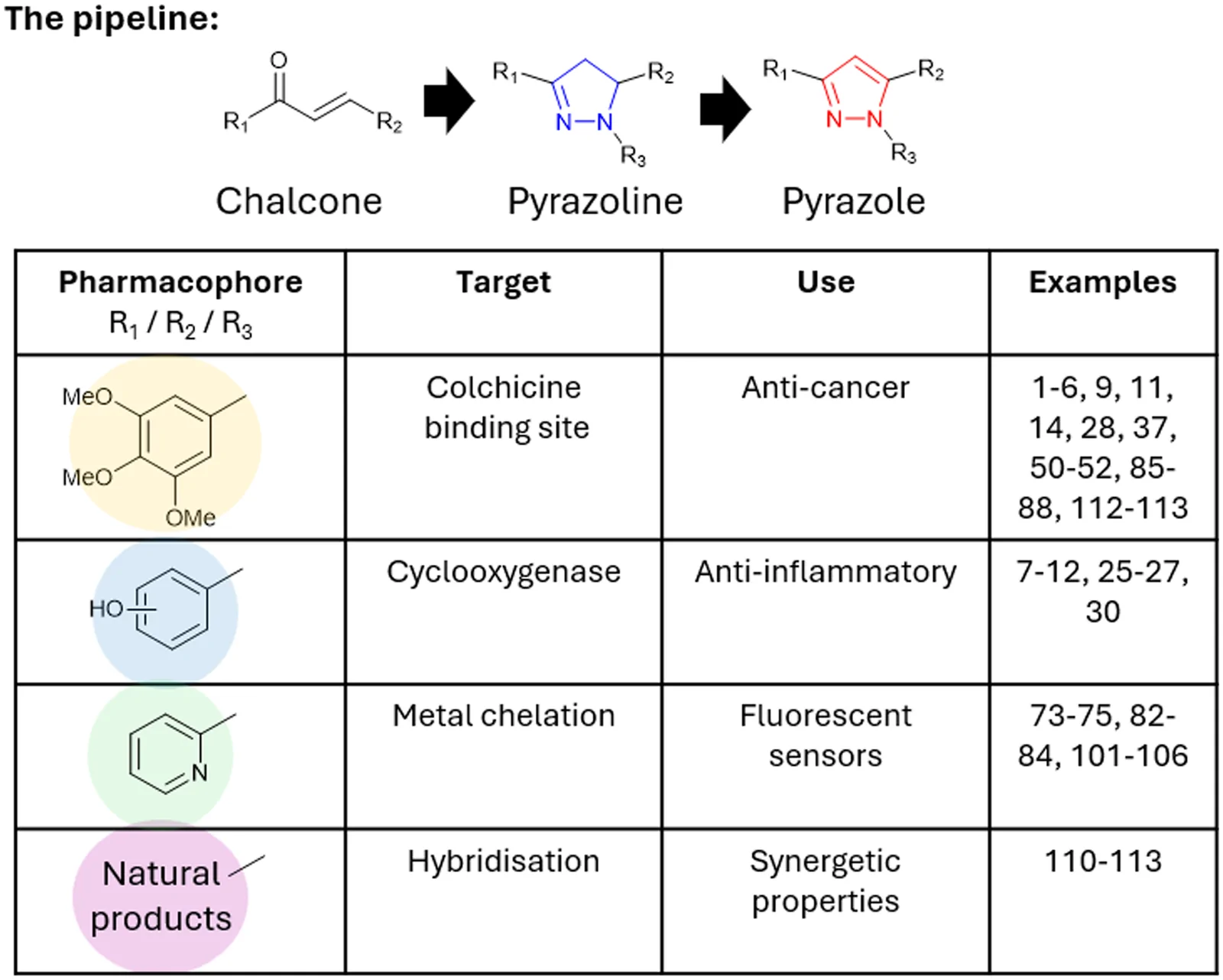
SAR summary.

A set of recurring pharmacophore motifs has emerged primarily responsible for the biological activities observed enabling a deeper understanding of the SARs associated with these scaffolds. The 3,4,5-trimethoxyphenyl unit consistently enhances cytotoxicity against cancer cell lines *via* binding to the colchicine binding site which disrupts tubulin formation (Fig. 28). Hydroxyphenyl substituents, particularly 4-hydroxyphenyl capable of hydrogen bonding are associated with anti-inflammatory activity *via* modulation of cyclooxygenase or NF-κB signalling pathways (Fig. 28). Heterocycles, such as pyridyl, confer metal-binding capacity that is exploited in fluorescent sensors. The hybridisation of different natural product motifs such as coumarin, artemisinin and quinoline foster synergetic biological properties. Crucially, these motifs are conserved as chalcone precursors are converted into pyrazolines and subsequent pyrazoles. This means that a single pharmacophore decision made at the chalcone stage propagates across the entire pipeline. Furthermore, we have highlighted how each scaffold has its own geometry which is intrinsically linked to its biological activity.

**Fig. 29:**
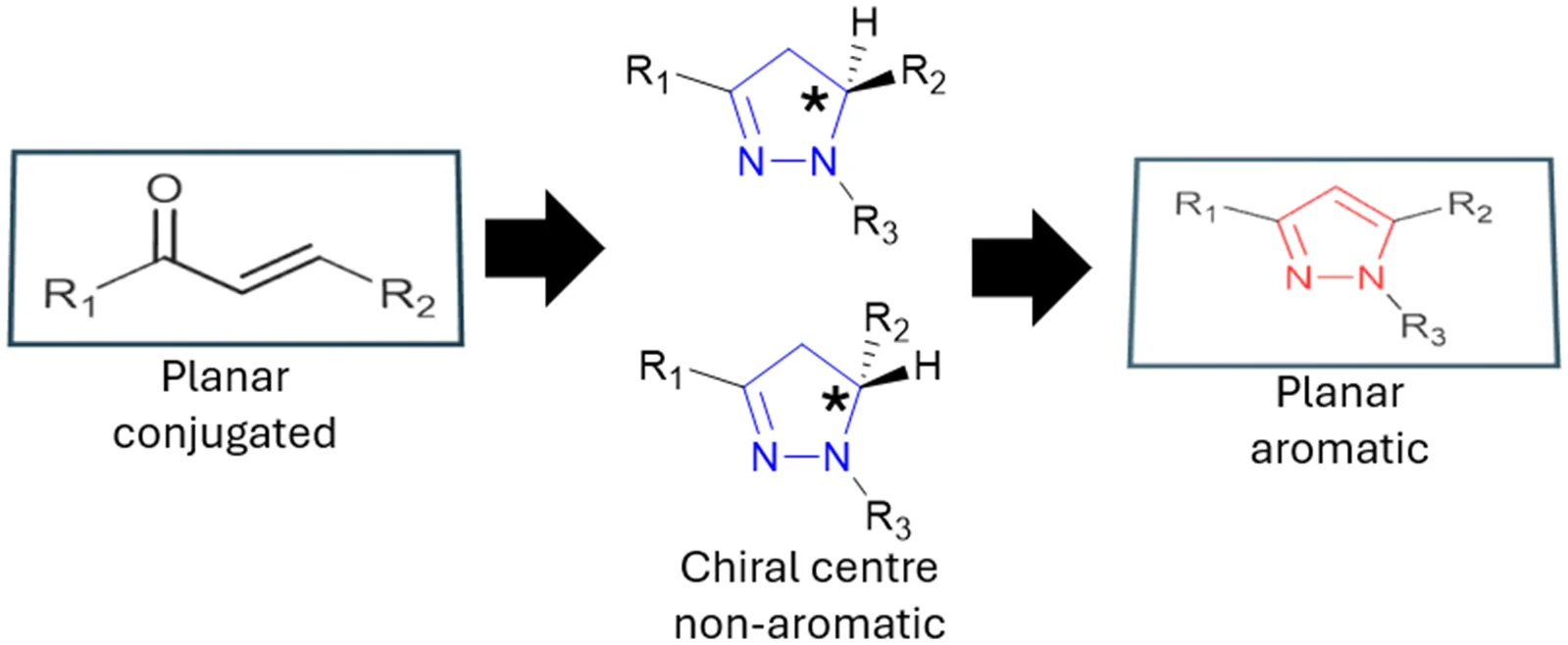
Scaffold geometry.

**Fig. 30:**
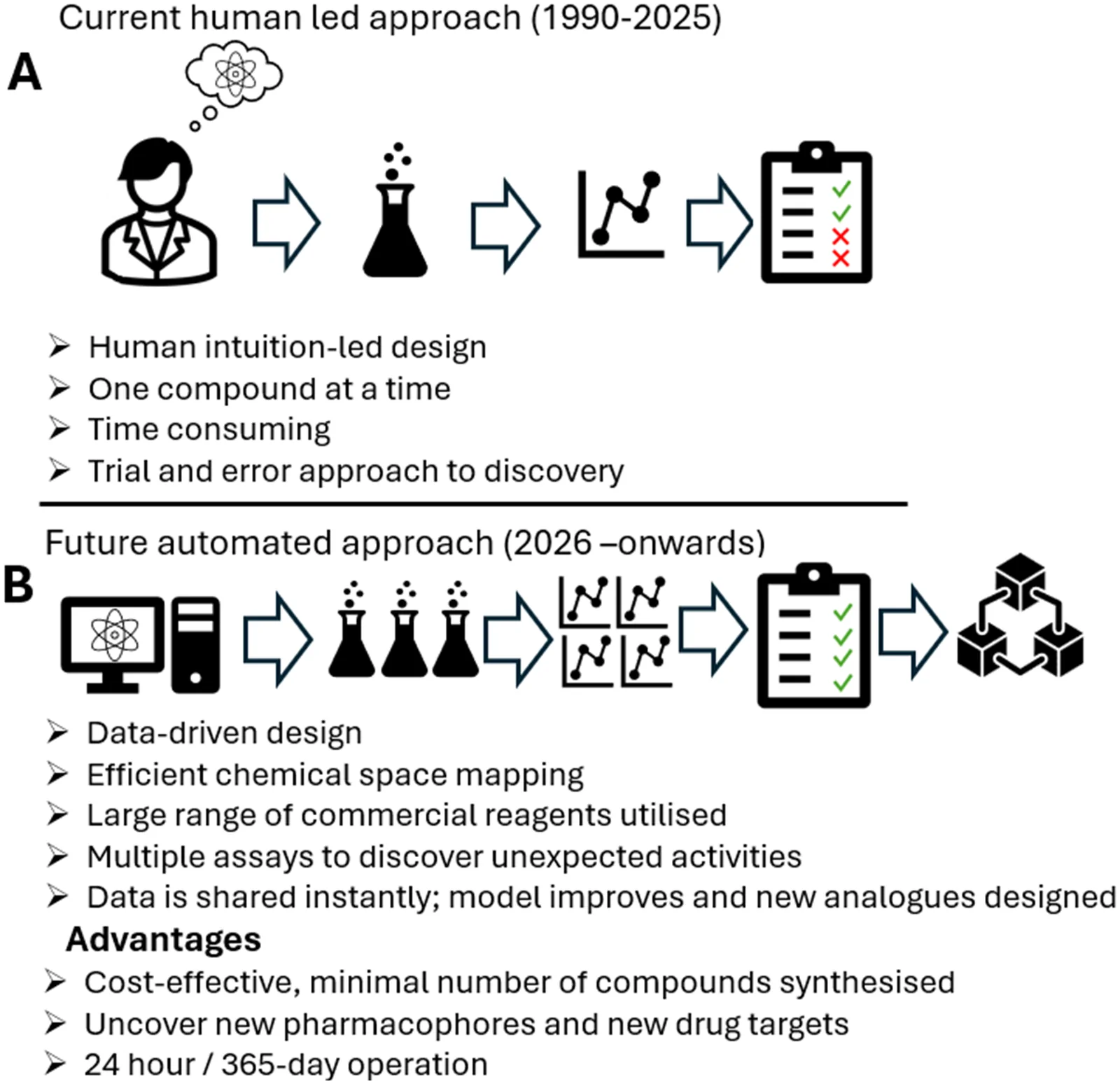
Current human-led SAR design (panel A) compared to future data-driven SAR approach exploring the full range of commercially available reagents (panel B).

The chalcone is planar enabling rigid positioning of the two R groups in opposing directions (Fig. 29), the conversion to pyrazoline introduces a third R group and a chiral centre at C-5 (* in Fig. 29) on the pyrazoline ring generating two enantiomers. Pyrazolines are often submitted for biological evaluation as a racemate however studies confirm that one enantiomer is typically more biologically active than the other. Conversion to the pyrazole removes the chiral centre restoring an aromatic planar pyrazole geometry. This significantly influences both the medicinal and fluorescent properties of each scaffold. These pharmacophores were developed over decades of time-consuming trial and error optimisation (Fig. 30A). As a result, the literature is well explored in certain areas but sparse or entirely unmapped in others. The scale of the unexplored space is considerable with over 680 000 possible chalcones from commercially available reagents alone. This presents a challenge in which new paradigms are needed with digital, data-driven approaches to SAR design (Fig. 30B) one solution. However, before we discuss the digital tools available, the clinical development of this pipeline over the last three decades offers several lessons for moving forward.

## 19 Clinical development

**Fig. 31:**
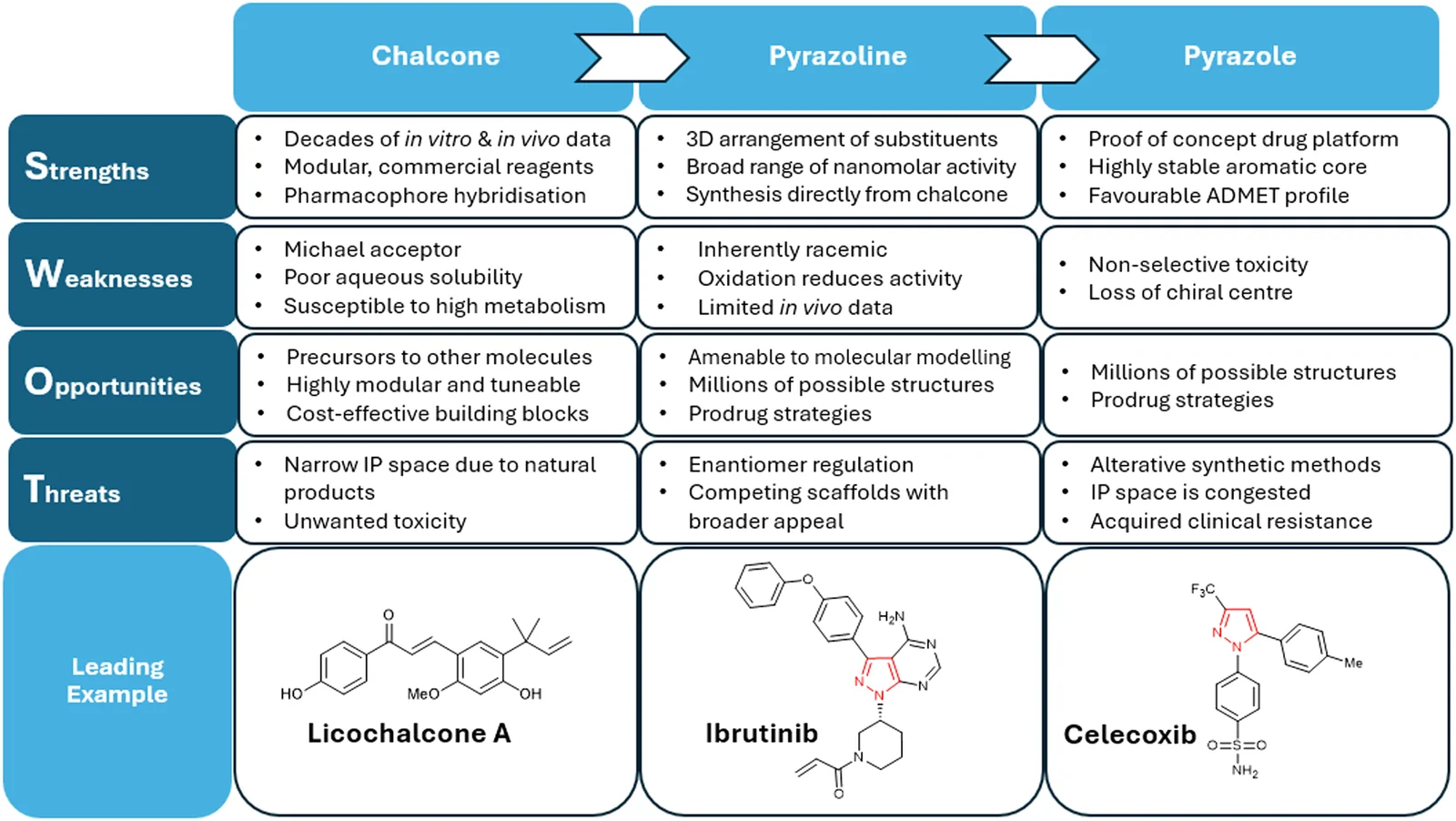
SWOT analysis for the clinical development of this pipeline.

Despite decades of investigation, the translation of chalcone, pyrazoline, and pyrazole candidates from laboratory to clinic remains limited. Understanding the intrinsic liabilities and mitigation strategies is essential if this pipeline is to reach its full therapeutic potential. From a clinical perspective, the most advanced chalcone is licochalcone A, a naturally occurring chalcone isolated from *Glycyrrhiza inflata*, which has been evaluated in multiple clinical trials as a topical treatment for acne.^170^ Its principal mechanism of action involves inhibition of the NF-κB signalling pathway, suppressing pro-inflammatory cytokine production.^171^ Topical application sidesteps the primary liability of the chalcone scaffold: the α,β-unsaturated carbonyl (enone) system to conjugate Michael addition by endogenous nucleophiles, including glutathione and cysteine residues generating off-target toxicity.^172^ In contrast, this weakness is often the mode of action for a variety of therapeutic activities observed.^173^ The enone is therefore a double-edged sword, providing intended and unintended biological activities.^174^ This electrophilic reactivity, when combined with poor aqueous solubility, presents significant barriers to clinical development. Prodrug strategies can mitigate these weaknesses, for example, a phosphate prodrug of the natural product isoliquiritigenin improved solubility facilitating *in vivo* studies.^175^ The hybridisation of cisplatin with a p53 targeting chalcone improved apoptosis when compared to each individual treatment is another notable example.^176^ Nevertheless, the chalcone scaffold itself is unlikely to advance as a stand-alone systemic therapeutic despite extensive preclinical trials involving chalcones reported.^177^ The greatest value of the chalcone scaffold lies in its role as a highly modular synthetic precursor for further drug scaffolds. A SWOT (strengths, weaknesses, opportunities, and threats) analysis summarises the clinical development of chalcones (Fig. 31). The pyrazole scaffold has a more established history with Ibrutinib, a tyrosine kinase inhibitor FDA-approved in 2013 for the treatment of chronic lymphocytic leukaemia^178^ and lymphoma.^179^ The principal strength of the pyrazoline ring for drug discovery is the introduction of a sp^3^ chiral centre at C-5 of the pyrazoline ring, enabling precise three-dimensional arrangement of substituents.^180^ However, the chiral centre introduces a significant practical issue, synthetic routes based on chalcone cyclisation with hydrazines are typically non-stereoselective, yielding racemic mixtures in which pharmacological activity is often confined to a single enantiomer while the other may be inactive.^181^ Strict clinical trial guidelines state pharmacological data must be obtained on both enantiomers before any human trials commence.^182^ Strategies to overcome this include purification *via* chiral semi-preparative HPLC^183^ or asymmetric synthesis are available,^184^ however with additional synthetic complexity and cost. A further liability is their susceptibility to aerial oxidation to the corresponding aromatic pyrazole eliminating the chiral centre and associated biological activity.^185^ The pyrazole scaffold represents the most clinically mature component of the pipeline. Celecoxib, a 1,3,5-diaryl pyrazole COX-2 selective inhibitor prescribed as an anti-inflammatory agent, particular for arthritis, is the prime example.^186^ Beyond celecoxib, eight FDA-approved protein kinase inhibitors contain a pyrazole ring, including ruxolitinib, avapritinib, asciminib, erdafitinib, crizotinib, encorafenib, pralsetinib, and pirtobrutinib are established. The greatest advantage of the pyrazole scaffold is the broad target compatibility, synthetic accessibility, and acceptable absorption, distribution, metabolism, excretion, and toxicity (ADMET) profile.^187^ The aromatic character of pyrazole confers metabolic stability relative to the pyrazoline, and the ability to fine-tune electronic and steric properties. Nevertheless, no pyrazole synthesised directly *via* a chalcone or pyrazoline has entered a phase I clinical trial. One additional feature present to all three scaffolds and one in which digital discovery tools could add significant value, is in assessing compound selectivity. Selectivity is broadly defined as intended *versus* unintended activity and is a common issue with these scaffolds. A selectivity index, for example activity in the required target *versus* off target activity should be reported, with SI ≥ 10 considered the minimum threshold for further development.^188^ Structural strategies to improve selectivity include introduction of tumour-targeting pharmacophores, exploitation of the overexpressed enzymes in specific cancer cell lines and restricting an activity profile through focused SAR. These preconditions could be configured into a digital discovery platform prioritising compounds exceeding predetermined thresholds for further development while flagging toxic compounds as problematic. This requires the synthesis and biological evaluation of thousands of compounds using standardised biological assays to generate large datasets of high-quality consistent data. The latest in artificial intelligence (AI) and machine learning (ML) algorithms learn underlying patterns predicting suitable analogues to efficiently map this chemical map. These, in turn, are synthesised and screened with the result used to reinforce and improve the predictive properties of the algorithms. This approach is transforming small molecule development for cancer immunotherapy^189^ and antibiotic discovery.^190^ We will now examine how the incorporation and convergence of digital discovery tools enable a more united, data-driven approach to the development of this pipeline.

## 20 Digital discovery tools

**Fig. 32:**
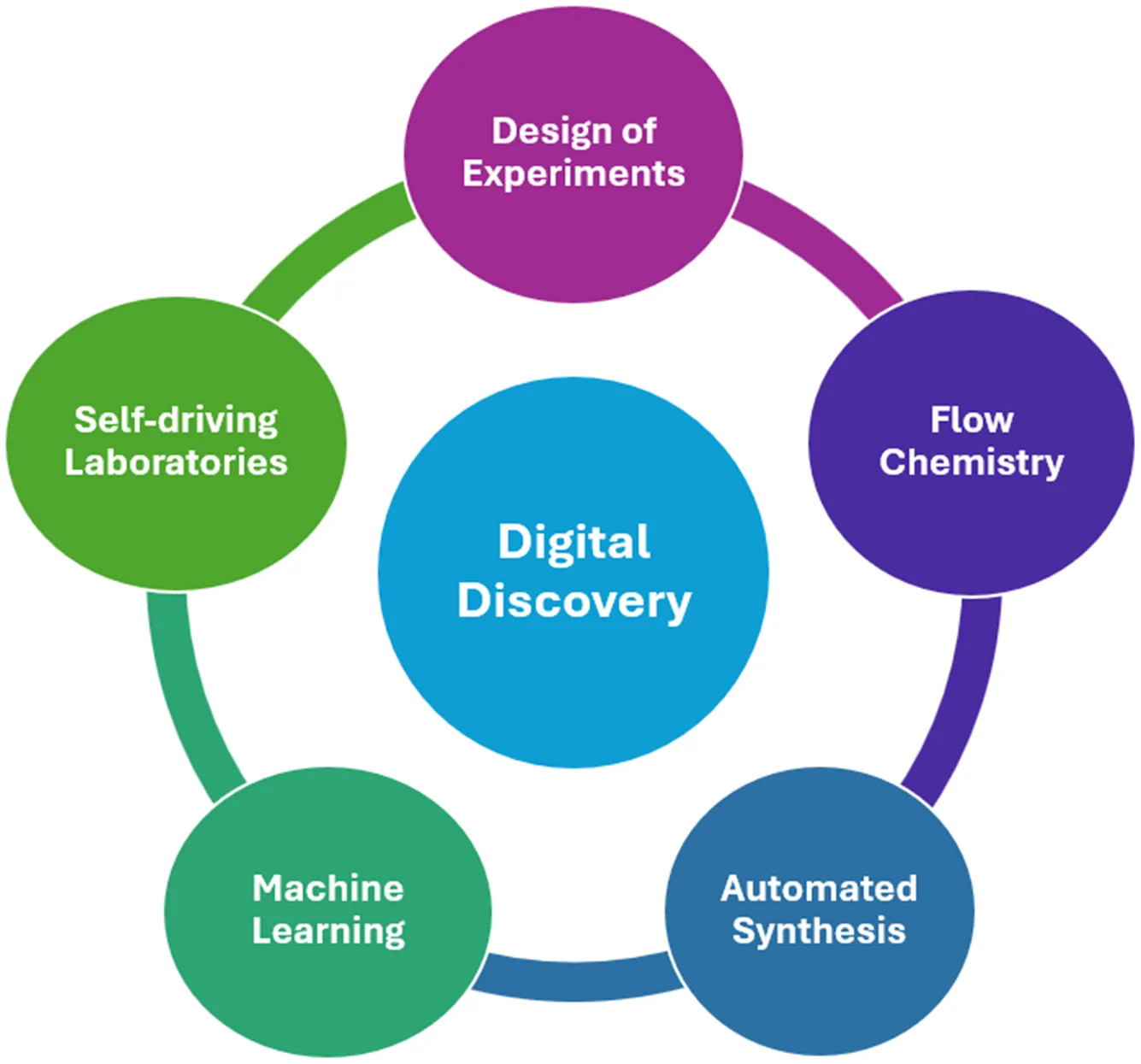
Five discovery tools to unlock the potential of this pipeline.

The age of digital chemistry, in which computational tools are employed to simulate, predict, generate, and analyse vast chemical datasets, is fundamentally reshaping how discovery is conducted.^191^ Such tools are unlocking unprecedented avenues of exploration, from structure–activity prediction to the automation of laborious experimental tasks and complex decision-making processes. Incorporating these approaches into the chalcone–pyrazoline–pyrazole pipeline offers a powerful means of navigating its vast, largely unexplored chemical space. Five aspects central to this process are examined here: the application of design of experiments (DoE) to define optimal synthetic conditions; flow chemistry for the efficient parallel production of compounds; automated synthesis platforms enabling continuous operation; machine learning of large datasets; and the integration of all these elements within a self-driving laboratory framework (Fig. 32).

## 21 Design of experiments

**Fig. 33:**
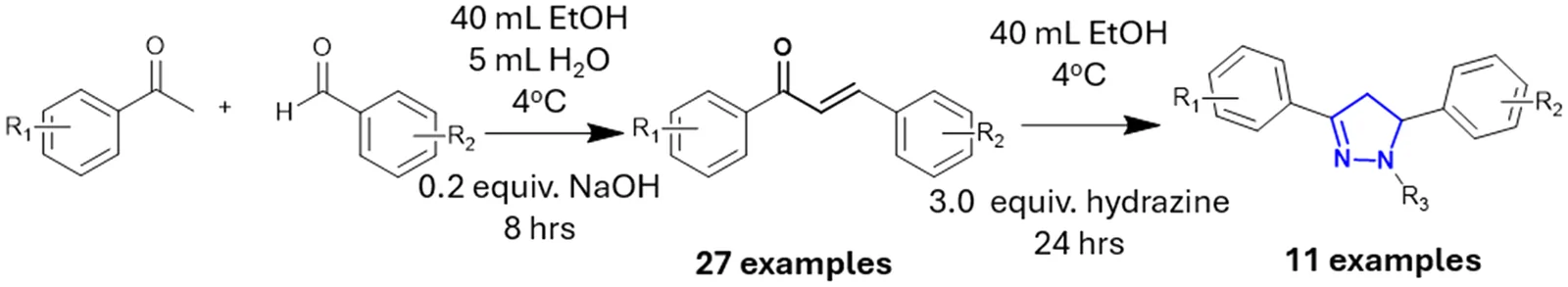
DoE synthetic conditions for chalcone and pyrazolines.

The ability to synthesise chalcones, pyrazolines and pyrazoles using the most efficient process is essential to the Design-Make-Test-Analyse (DMTA) cycle at the core of this discovery pipeline.^192^ Design of experiments (DoE) devised by Fisher in 1935 varies multiple factors (solvent, temperature, catalyst and time) simultaneously within a structured statistical design, enabling the modelling of interactions and prediction of optimal parameters.^193^ DoE is a considerably more efficient approach than one factor at a time (OFAT) optimisation, particularly for multivariable syntheses. Recent examples of DoE in chemistry include B(OCH_2_CF_3_)_3_-mediated direct amidation,^194^ Heck–Suzuki reaction optimisation^195^ and benzimidazol-2-one synthesis.^196^ A recent DoE study examined over 105 data points to establish optimised conditions for the synthesis of 27 chalcones and their subsequent conversion into 11 pyrazolines (Fig. 33).^132^

The use of 0.2 equiv. NaOH in MeOH at 4 °C afforded chalcone formation in under 8 hours with yields of up to 95%. When performed on a 10 mmol scale, these conditions delivered gram-scale chalcones directly *via* recrystallisation, eliminating the need for time-consuming column chromatography. Applying a comparable DoE strategy to the pyrazoline-forming step yielded the following optimised conditions for chalcone-to-pyrazoline conversion: 3.0 equiv. hydrazine, 4 °C, EtOH, 24 hours (Fig. 33). There are only a few examples of DoE applied to pyrazoles, for example electrochemical decarboxylative alkylation^197^ and the Knorr synthesis of pyrazole.^198^ There is yet to be a DoE study specifically on the pyrazoline to pyrazole transformation or direct chalcone to pyrazole synthesis and this is a key research gap to address. This is essential to move to the next phase of the process, the incorporation of flow chemistry.

## 22 Flow chemistry

**Fig. 34:**
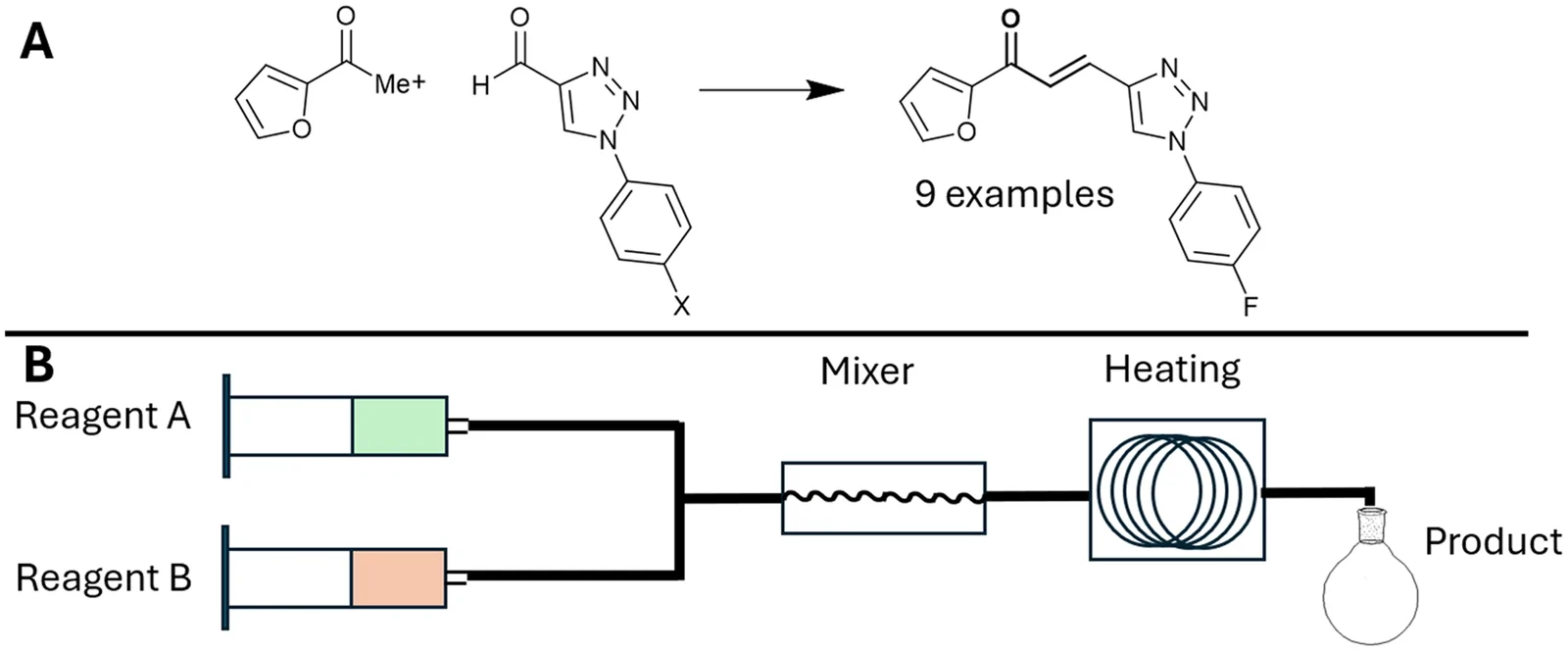
Flow chemistry for chalcone synthesis, panel A for reaction synthesis, panel B for experimental design.

**Fig. 35:**
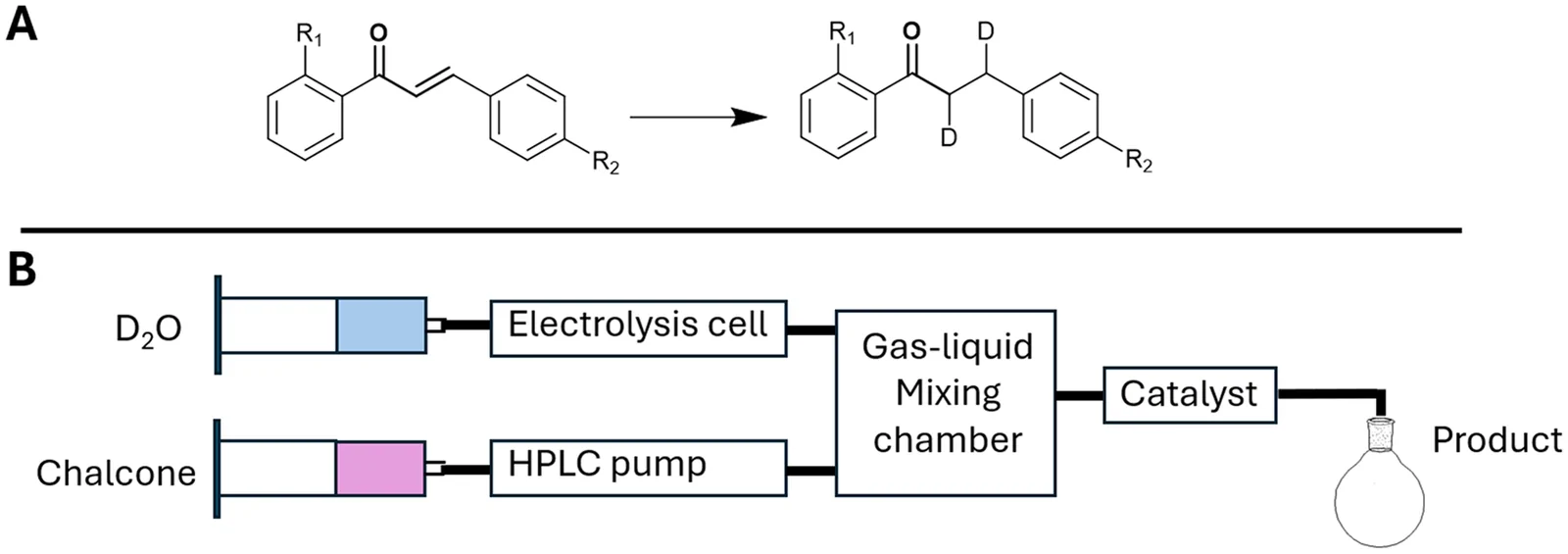
Flow chemistry for deuterium-labelled chalcones, panel A for reaction synthesis, panel B for experimental design.

The traditional approach to compound synthesis has been a batch process in which reagents are combined in a single vessel to generate one compound at a time. However, flow chemistry (or continuous-flow chemistry) represents a fundamental paradigm shift, moving synthesis away from static flasks into a continuous stream of moving fluids.^199^ By pumping reactants through microscopic tubes or precisely engineered channels, this approach replaces bulky, traditional reactors with dynamic, highly controlled microenvironments. This transition from macro-scale batching to micro-scale flow unlocks unprecedented control over reaction parameters, transforming how we approach chemical synthesis from the laboratory bench to industrial manufacturing.^200^ The ability to perform on-line analysis^201^ is also valuable to ensure compounds are extracted when synthesis is complete preventing time and energy wastage. Flow chemistry offers significant advantages when performing high-throughput screening and experimentation.^202^ To date, there are limited examples of applying flow chemistry to this pipeline. Ganesh *et al.* reported the synthesis of nine 1,2,3-triazole-furan hybrid chalcone derivatives under flow conditions.^203^ The resulting chalcones were screened for anti-microbial activity with the most prominent bearing a single fluorine substituent (Fig. 34). Fülöp *et al.* labelled a range of chalcones with deuterium under flow conditions which lacked the disadvantages of classical batch methods using harsh and/or expensive catalysts (Fig. 35).^204^

**Fig. 36:**
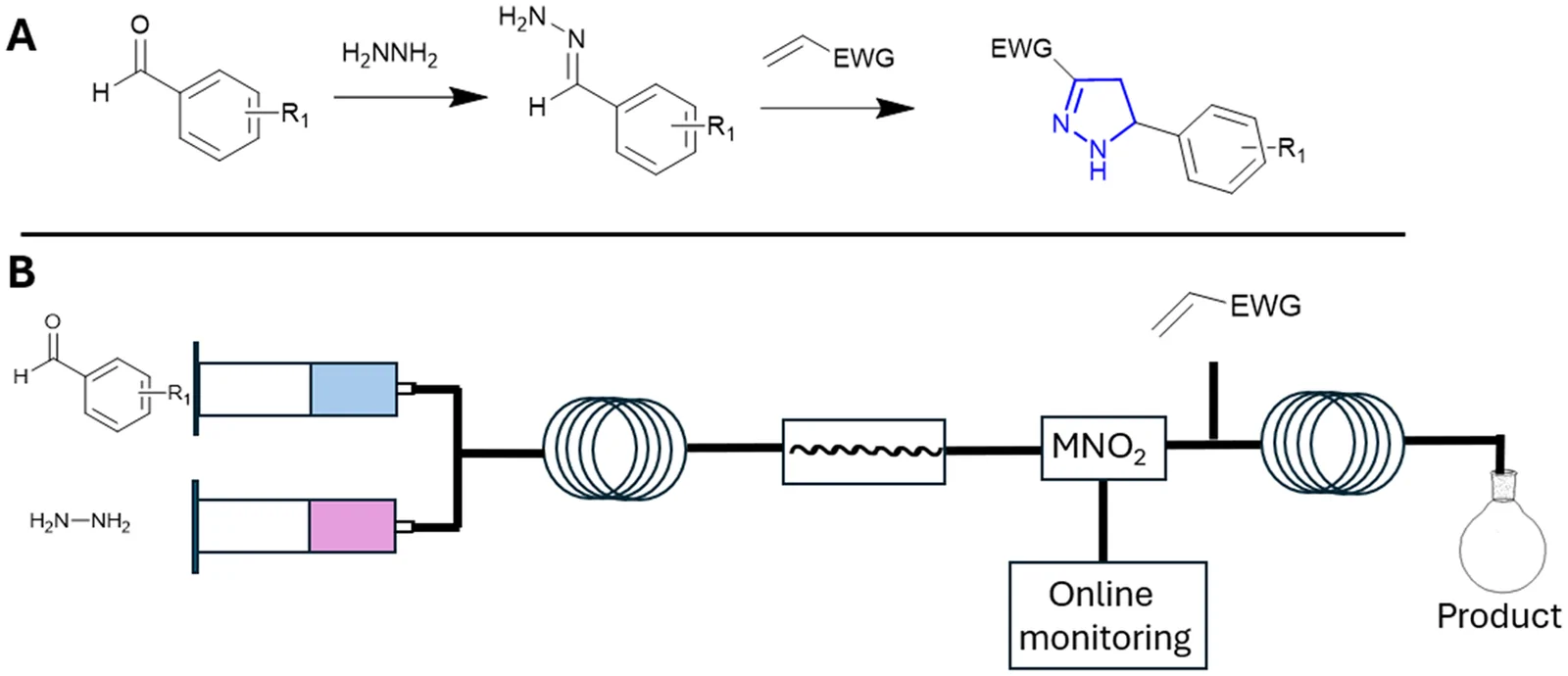
Flow chemistry for pyrazoline synthesis, panel A for reaction synthesis, panel B for experimental design.

Ley *et al.* reported an elegant synthesis of twenty substituted pyrazolines *via* [3 + 2]cycloaddition reaction of diazo compounds with a MnO_2_ catalyst (Fig. 36).^205^ Performing this reaction under flow conditions with online reaction monitoring enabled automation of this process with minimal human intervention. Jamison *et al.* applied flow chemistry to generate over thirty-three highly substituted pyrazolines and pyrazoles *via* an automated assembly-line workflow.^206^ The conversion of anilines to pyrazoles *via* flow chemistry was also reported.^207^ While these pyrazolines and pyrazoles were not synthesised directly from chalcones, they provide proof-of-concept and validate the significant advantages flow chemistry. There remains a research need to apply flow chemistry directly to the chalcone-pyrazoline-pyrazole pipeline. The application of automated synthesis platforms provides further examples of incorporating such approaches in modern materials discovery.

## 23 Automated synthesis

**Fig. 37:**
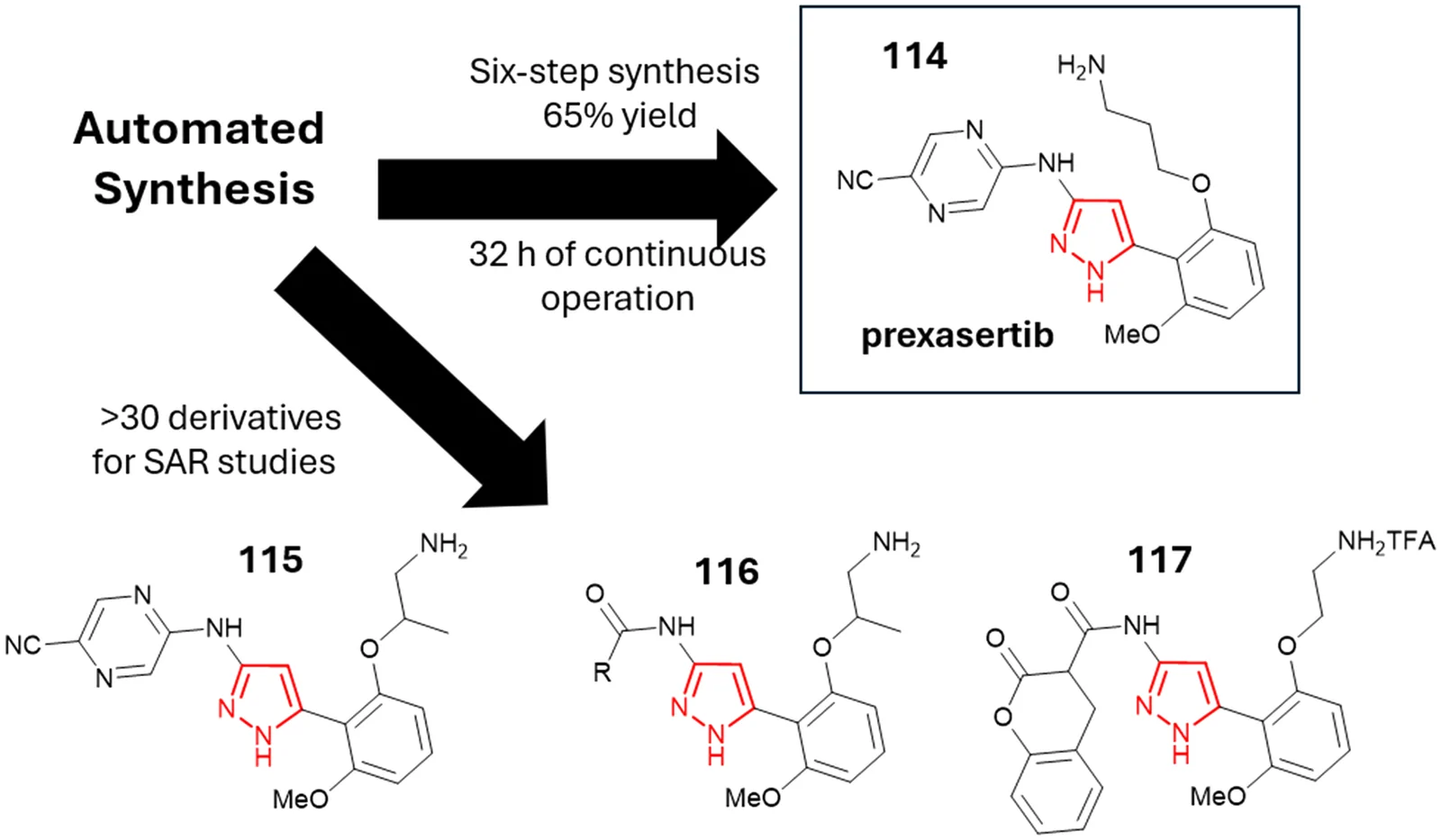
Automated synthesis of pyrazoles.

Traditional bench chemistry, while responsible for many of the examples discussed within, is inherently labour-intensive and ill-suited to exploration of the 7.5 million possible compounds this pipeline can provide.^1^ Automated synthesis platforms address this directly, executing repetitive synthetic tasks with precision and consistency that frees the chemist to focus on experimental design, data interpretation, and the creative aspects of discovery that remain uniquely human. A leading example in automated synthesis reported by Wu *et al.* applied to a kinase inhibitor prexasertib 114 (Fig. 37).^207^ This drug was synthesised *via* a six-step fully automated synthesis with 65% isolated yield following 32 hours of unattended continuous operation. Alongside prexasertib itself, twenty-three derivatives, including pyrazolines 115–117 were also produced without the need to reconfigure the platform. This work illustrates how automated synthesis platforms can greatly accelerate the Design-Make-Test-Analyse (DMTA) cycle *via* a fully self-contained system. Further work is needed to apply such technologies to chalcones and pyrazolines with the study by Wu *et al.* exemplifying the significant advantages on offer.

## 24 Machine learning

**Fig. 38:**
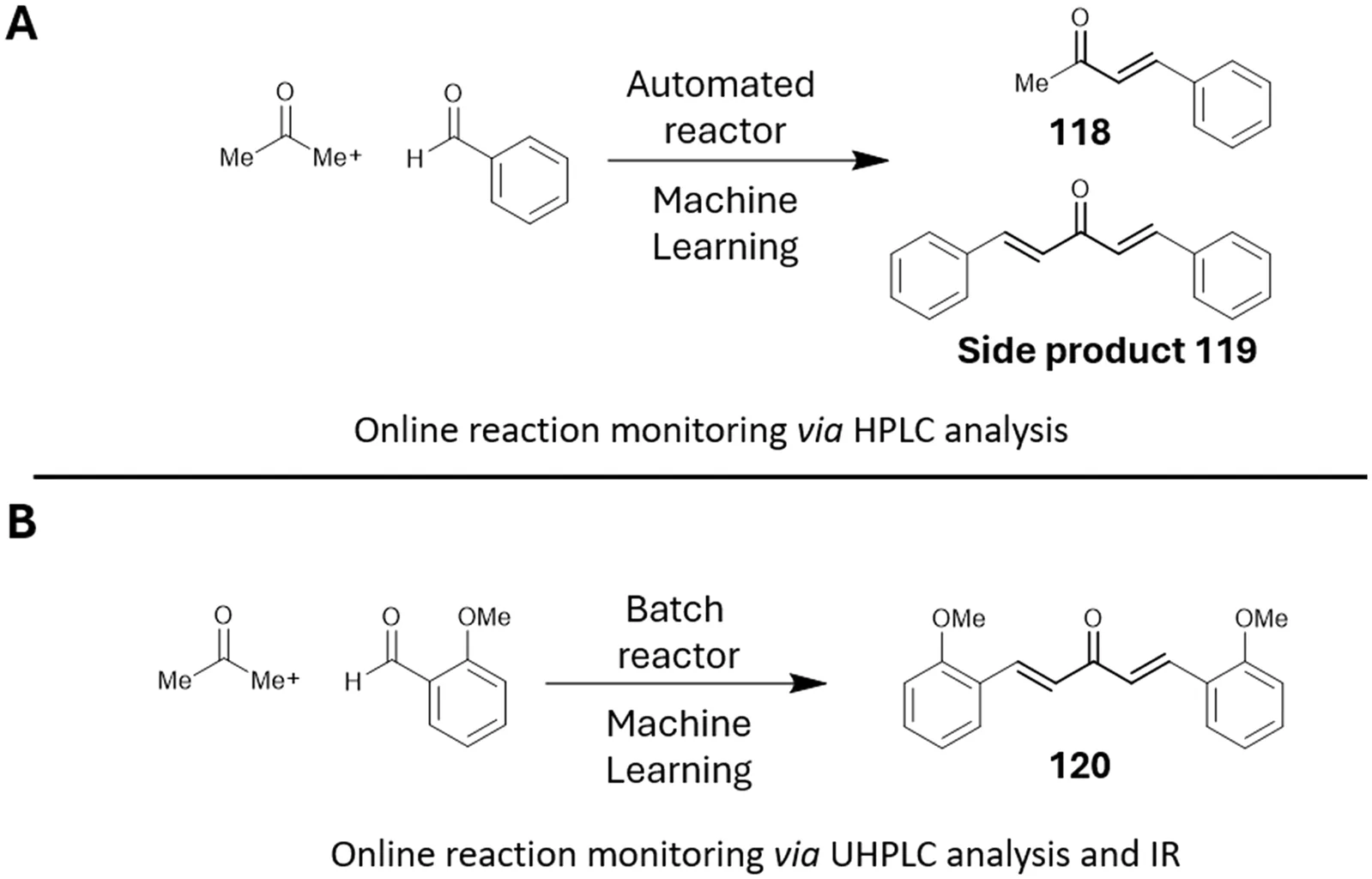
Machine learning to optimise synthesis of chalcone 118 in panel A and chalcone 120 in panel B.

Machine learning (ML) is a specific subset of artificial intelligence (AI) that allows computers to learn from data and improve at tasks over time without being explicitly programmed.^208^ Machine learning offers a transformative approach as models can learn continuously from experimental data and adapt in real time. When coupled with automated flow platforms, this creates a closed-loop system simultaneously balancing yield, selectivity, throughput, and resource without human guidance. The Claisen–Schmidt condensation, the foundational reaction in chalcone synthesis has emerged as a productive testbed for such approaches. One study by Bourne *et al.* used ML to determine optimal conditions for the synthesis of product 118 from the reaction of acetone and benzaldehyde while minimising the formation of side product 119 (Fig. 38A).^209^ HPLC provided the input for the model which once optimised yielded 118 with negligible (<1%) formation of 119. A similar approach was applied by Kopać *et al.* for the formation of product 120 also using ML (Fig. 38B).^210^ Both approaches demonstrate the advantages of incorporating automated self-optimizing systems into chalcone synthesis using ML methods when compared to traditional batch methods of optimisation.

**Fig. 39:**
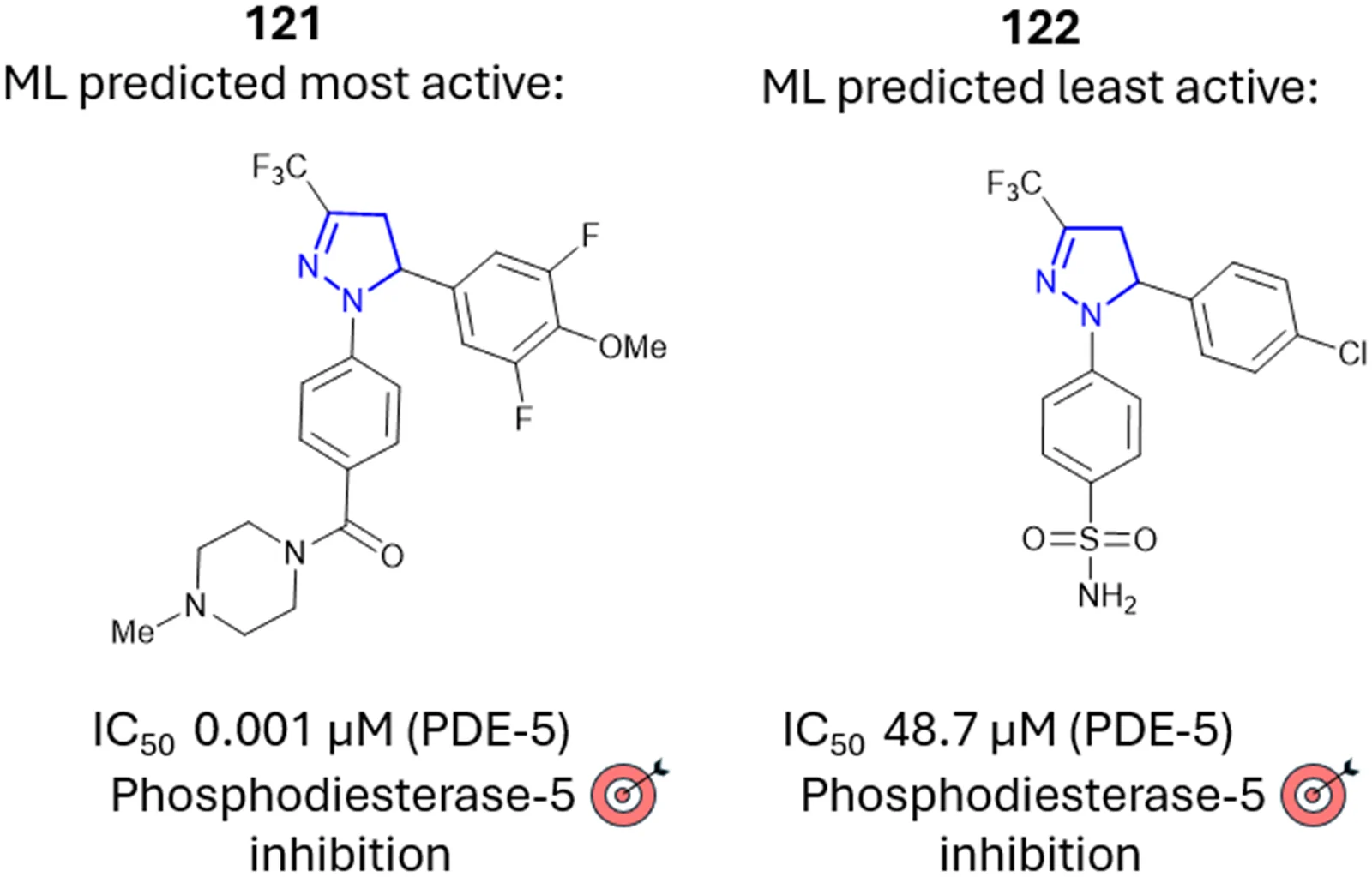
Machine learning for lead compound identification.

The application of ML is not limited to synthesis; Nandi *et al.* reported the ML based SAR analysis on a dataset of over sixty tri-substituted pyrazolines with IC_50_ values reported in the literature.^211^ This dataset was split into training and test datasets and a range of ML algorithms applied including k-nearest neighbours, random forest and logistic regression.^211^ The ML predicted lead compound was confirmed as pyrazoline 121 and the predicted least active compound was predicted and confirmed as pyrazoline 122 (Fig. 39). Two further applications of ML for the prediction of biological activity include ML SAR for flavones and ML SAR for cyclin dependent kinases.^212,213^ In summary ML has an important part in the future development of this pipeline. As yet there are limited examples of ML tools directly on chalcone, pyrazolines or pyrazoles and this presents a key research gap going forward.

## 25 Self-driving laboratories

**Fig. 40:**
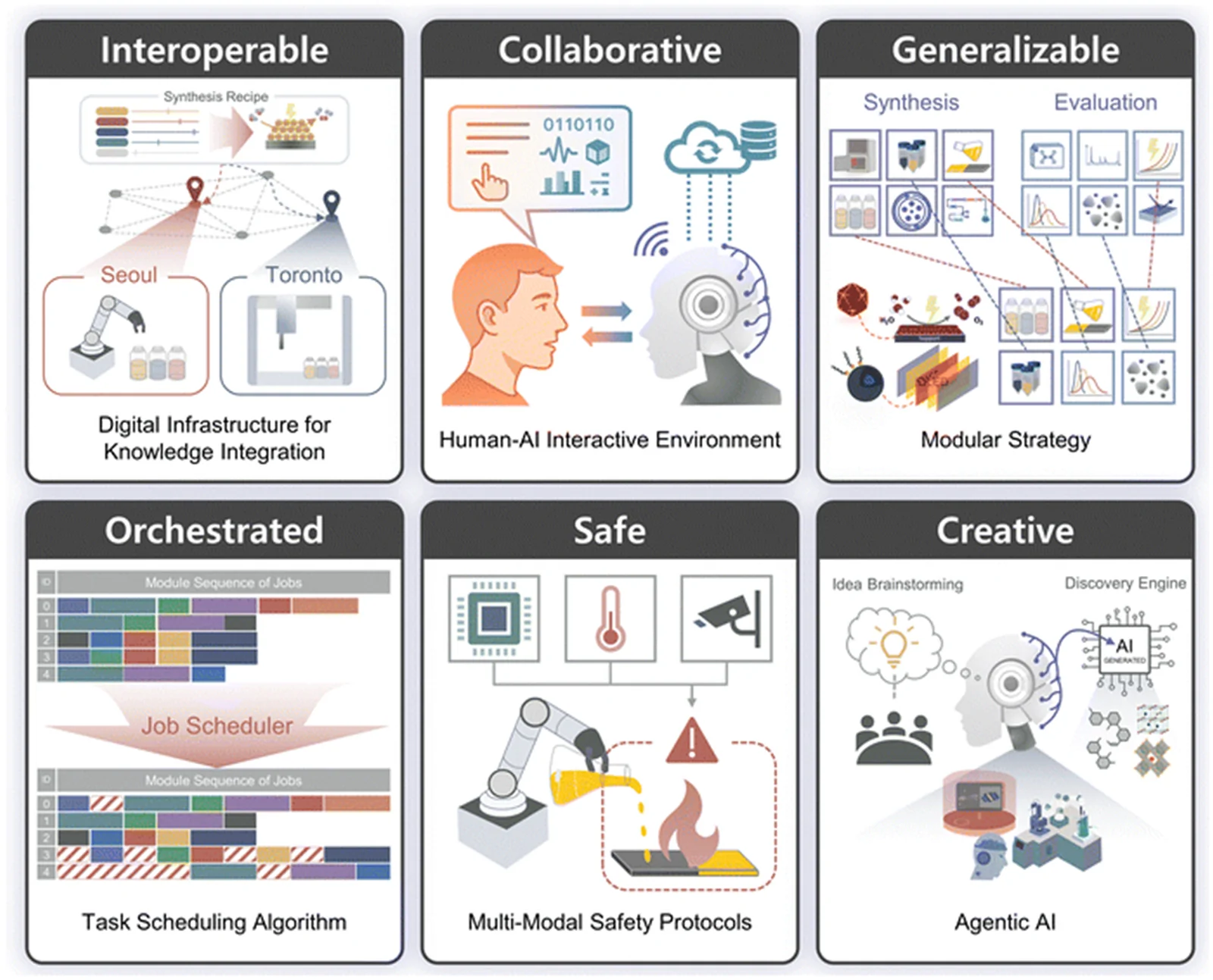
Six features of a self-driving laboratory, reproduced from ref. 214*a*, permission from RSC^214*a*^ copyright 2026.

We have introduced four digital tools; design of experiments, flow chemistry, automated synthesis, and machine learning with each capable of independently accelerating the chalcone–pyrazoline–pyrazole discovery pipeline. Yet their greatest power lies not in isolation but in integration. The self-driving laboratory (SDL) represents the logical convergence of these technologies into a single, closed-loop discovery system.^214^ First conceptualised as an extension of laboratory automation, SDLs have matured into sophisticated autonomous research platforms capable of operating continuously, making decisions independently, and compressing experimental timescales from months to days. Han *et al.* proposed six key features of a successful SDL for chemistry and materials discovery (Fig. 40).^214^

**Fig. 41:**
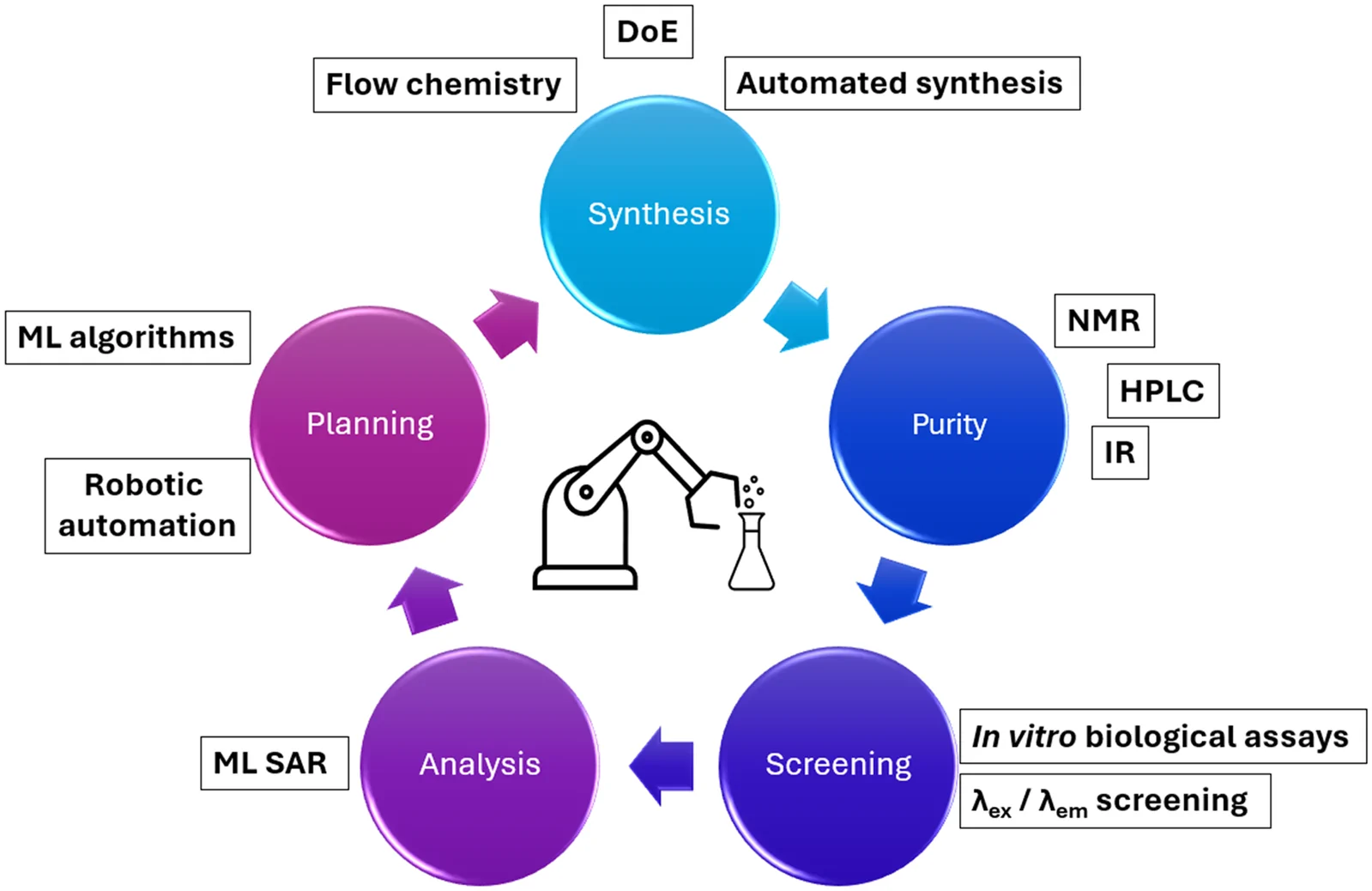
Closed-loop synthesis of the proposed pipeline.

To date, no self-driving laboratory has been applied to chalcone, pyrazoline, or pyrazole discovery, a significant research gap given the therapeutic and sensing potential these scaffolds collectively represent. Self-driving laboratories broadly fall into two architectural categories: fixed robotic systems,^215^ in which automated equipment is physically integrated within specific analytical instruments, and mobile robotic systems, in which autonomous robots navigate a conventional laboratory environment. Fixed platforms – exemplified by liquid-handling robots, offer high throughput, precise volumetric control, and robust reproducibility but are constrained by their configuration and lack reconfigurability. Mobile robotic systems can navigate existing laboratory infrastructure and operate side by side with human chemists in a shared environment.^216^ This allows the sharing of scientific equipment, saving on the purchase of bespoke apparatus, however working alongside humans introduces additional health and safety requirements. For this pipeline, access to multiple equipment will be required therefore a mobile robotic architecture is the more appropriate long-term framework (Fig. 41).

**Fig. 42:**
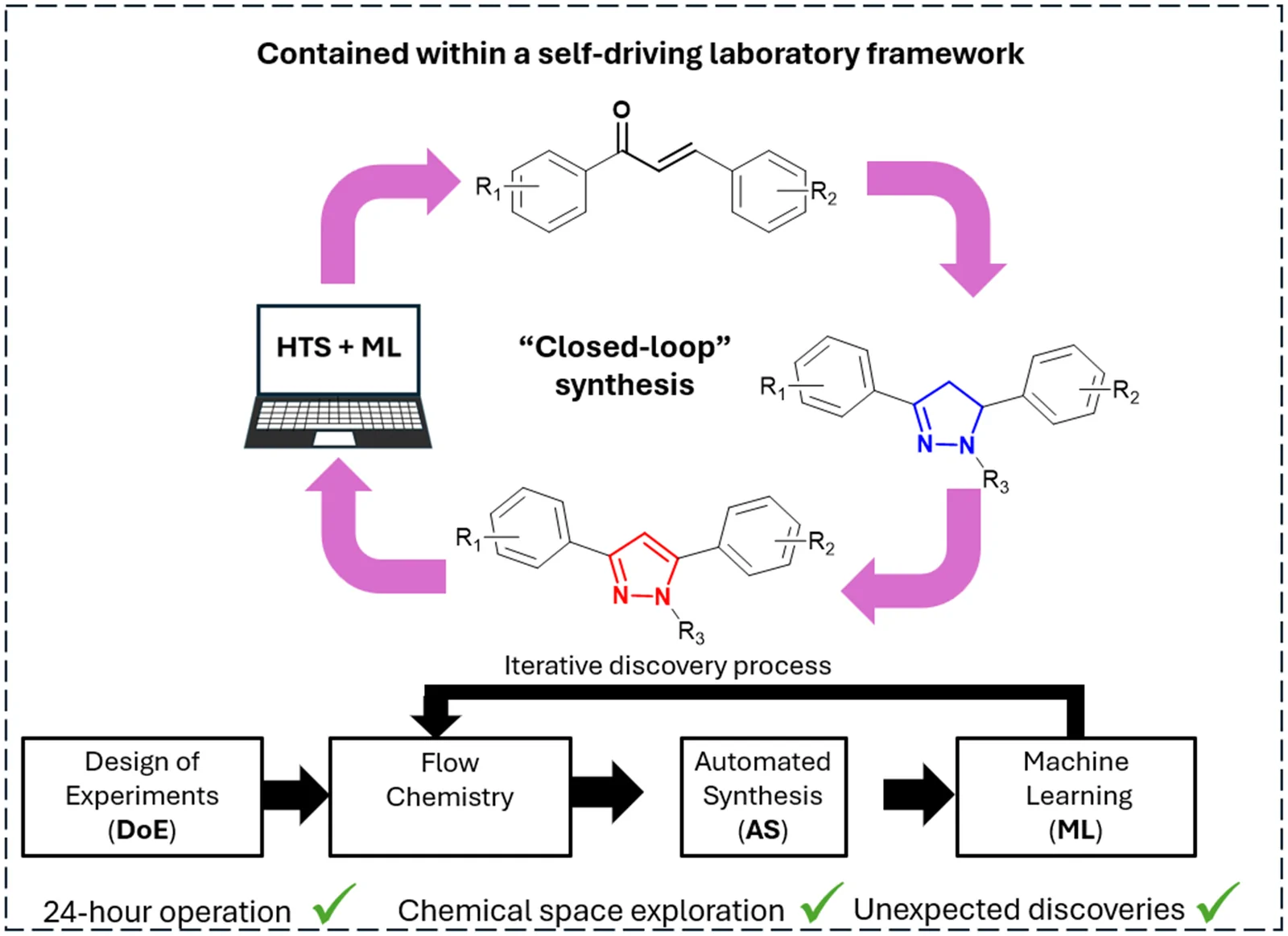
“Closed-loop” synthesis of the chalcone, pyrazoline and pyrazole privileged pipeline *via* digital tools within a self-driving laboratory.

The ability to synthesise, characterise and prepare samples for *in vitro* biological assays alongside screen for useful fluorescence properties is essential. In summary, the synergistic utilisation of DoE, flow chemistry, automated synthesis, and machine learning, all operating within a mobile robot SDL framework is ideal. This workflow will enable vast datasets to be generated with 24-hour operation and offer a means of systematically exploring this chemical space estimated to exceed 7.5 million accessible compounds from commercially available starting materials alone (Fig. 42). Such an approach is essential if this pipeline is to reach its full potential.

## 26 Green chemistry and digital sustainability

**Fig. 43:**
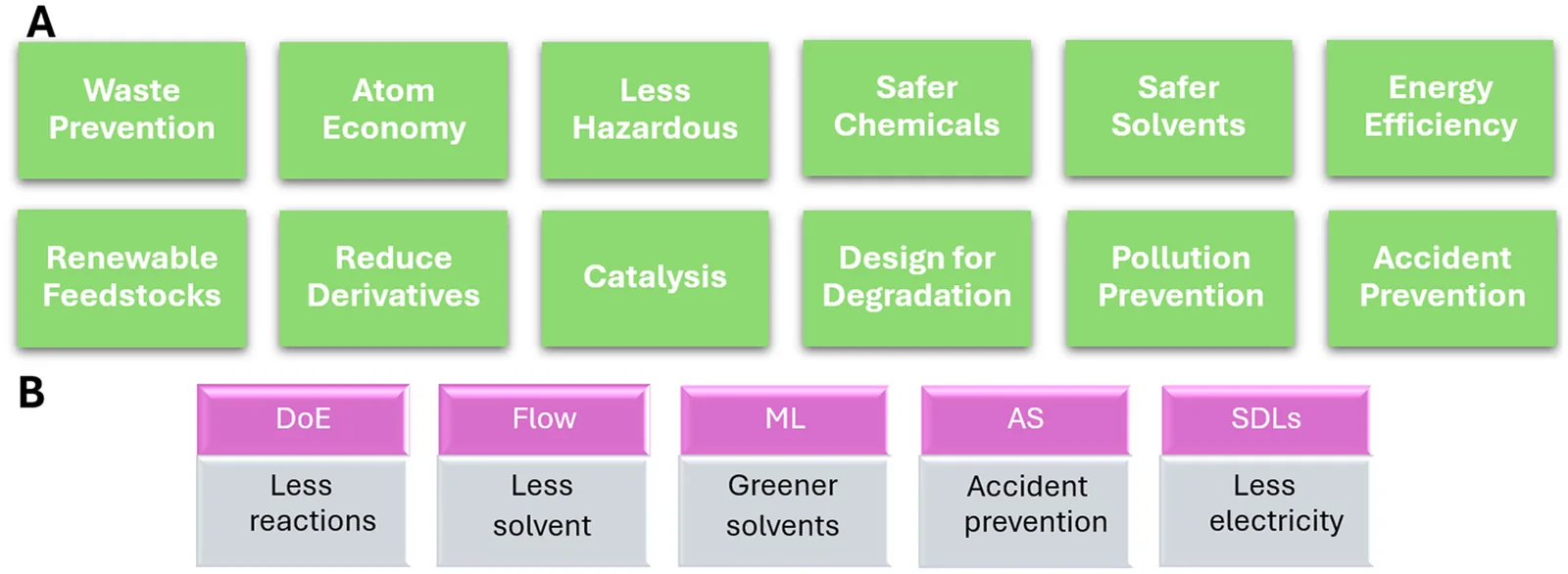
The 12 principles of green chemistry (panel A) and the potential of digital tools to achieve these goals (panel B).

Green chemistry is founded on twelve principles designed to reduce or eliminate the use and generation of hazardous substances in chemical products and processes (Fig. 43A).^217^ As well as accelerating the discovery process, digital tools have a valuable role in meeting several of these principles (Fig. 43B). For example, the use of design of experiments to determine optimal reaction conditions using the minimum number of experiments and flow chemistry reduce the number of reactions, solvent volume and reaction times.

**Fig. 44:**
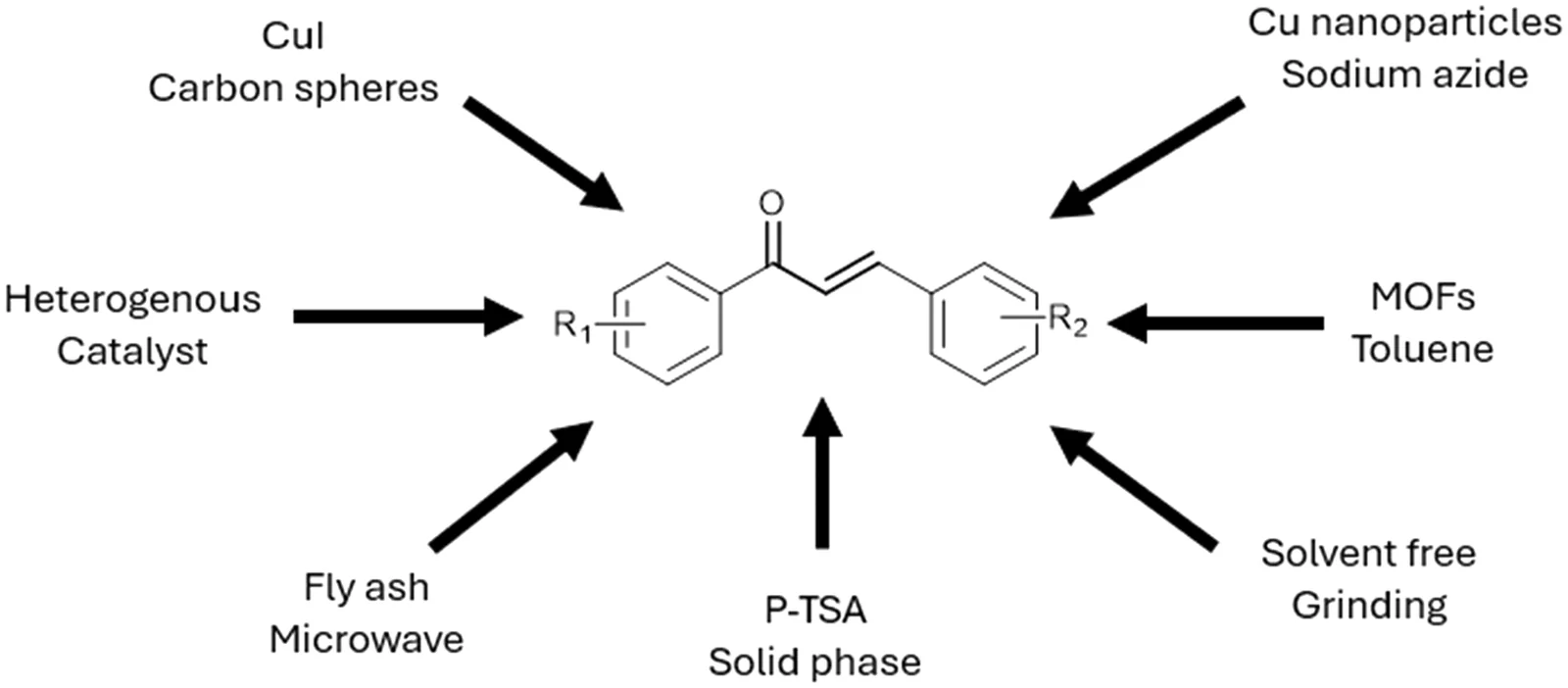
Recently reported green chemistry approaches to chalcone formation.

Machine learning can be used to predict optimal solvent and catalyst conditions increasing atom efficiency and reducing waste product formation. The use of automated synthesis platforms removes the human from the traditional laboratory bench reducing exposure to harmful chemicals and potential accidents. The use of self-driving laboratories operate continually 24 hours, 365 days a year ensuring laboratory instrumentation is used efficiently reducing idle time. The environmental factor (E-factor) is an established metric^218^ to measure sustainability of a chemical process and is defined as the total mass of waste/mass of product with a E-factor of zero for a process producing zero waste. The incorporation of the 12 principles of green chemistry and the use of E-factors should be incorporated into the use of digital tools as standard. This could easily be achieved by utilising the extensive use of green chemistry already applied to the synthesis of chalcones, pyrazolines and pyrazoles. For example, the use of solvent-free grinding,^219^ nanoparticle catalysis,^220^ microwave reactors,^221^ solid phase catalyst^222^ and ultrasonic irradiation^223^ (Fig. 44) are a small selection of such approaches applied to the synthesis of chalcones alone. Similar approaches have been applied to pyrazolines^224^ and pyrazoles.^225^ The incorporation of such novel synthetic approaches into the above digital tools will accelerate and greatly increase the sustainability of the discovery process.

## 27 Conclusions and outlook

The past three decades have established chalcones, pyrazolines, and pyrazoles as highly versatile frameworks spanning both medicinal chemistry and fluorescent sensing applications. Across these scaffold classes, simple synthetic transformations from inexpensive and commercially available precursors enable rapid access to structurally diverse compounds with significant biological and photophysical activity. Notable examples highlighted in this review include chalcone 2 (Fig. 3), pyrazoline 52 (Fig. 13), and pyrazole 86 (Fig. 21), each demonstrating nanomolar anti-cancer potency. Chalcone 21 (Fig. 6) and pyrazoline 64 (Fig. 15) exhibited promising anti-inflammatory profiles alongside pyrazolines such as 66 (Fig. 16) and 71 (Fig. 17) illustrate the potential in addressing pressing global challenges such as coronavirus and malaria respectively. These scaffolds should not be viewed in isolation, they form a synthetic and functional pipeline, in which each transformation leads to the next. On this basis, we introduce the concept of a “privileged pipeline”, defined as a linear synthetic sequence in which successive transformations yield multiple privileged scaffolds that can be systematically explored as a unified and diverse discovery platform. To our knowledge, this terminology has not previously been described. Despite the substantial progress made, the scale of the accessible chemical space remains largely underexplored with over 7.5 million theoretical compounds from commercially available reagents within this pipeline alone. The hybridisation of well-established pharmacophores multiplies this estimate even further. We are unable to explore this vast chemical space using human intuition and traditional bench chemistry alone, the integration of digital discovery tools is essential. Design of experiments, flow chemistry, automated synthesis platforms and machine learning are required. When combined within a self-driving laboratory framework, these tools form a closed-loop system capable of continuously designing, synthesising, and evaluating new compounds with minimal human intervention 24 hours, 7 days a week (Fig. 41). Such an integrated approach is ideally suited to this pipeline, where modular synthesis, structural diversity, and multiple activities create an ideal environment for data-driven discovery. The use of digital tools can also greatly accelerate the adoption of green chemistry and meet the objectives of the 12 principles of green chemistry (Fig. 43). By adopting a synergistic strategy in which automated synthesis, high-throughput screening, and machine learning reinforce one another, the full potential of this pipeline can be realised.

## Author contributions

Alexander Ciupa authored the manuscript.

## Conflicts of interest

There are no conflicts to declare.

## Supplementary Material

MD-OLF-D6MD00424E-s001

## Data Availability

No primary research results, software or code have been included and no new data were generated or analysed as part of this review. Supplementary information (SI) is available. See DOI: https://doi.org/10.1039/d6md00424e.
