# Supplementary material for: Three privileged scaffolds, one pipeline: chalcones, pyrazolines and pyrazoles in drug discovery and fluorescent sensing
Source: RSC Med Chem. 2026 Aug 20;17(10):4742–65. doi: 10.1039/d6md00424e (PMC13539734; doi:10.1039/d6md00424e)
Supplement: Supplementary file 1 [file MD-017-D6MD00424E-s001.pdf]

Supplementary Information (SI) for RSC Medicinal Chemistry.
This journal is © The Royal Society of Chemistry 2026

ESI: Three privileged scaffolds, one pipeline: chalcones, pyrazolines and pyrazoles in drug discovery and fluorescent sensing

Alexander Ciupa*

ESI 1

literature search for pyrazoles via the Reaxys database between 1990 and 2025

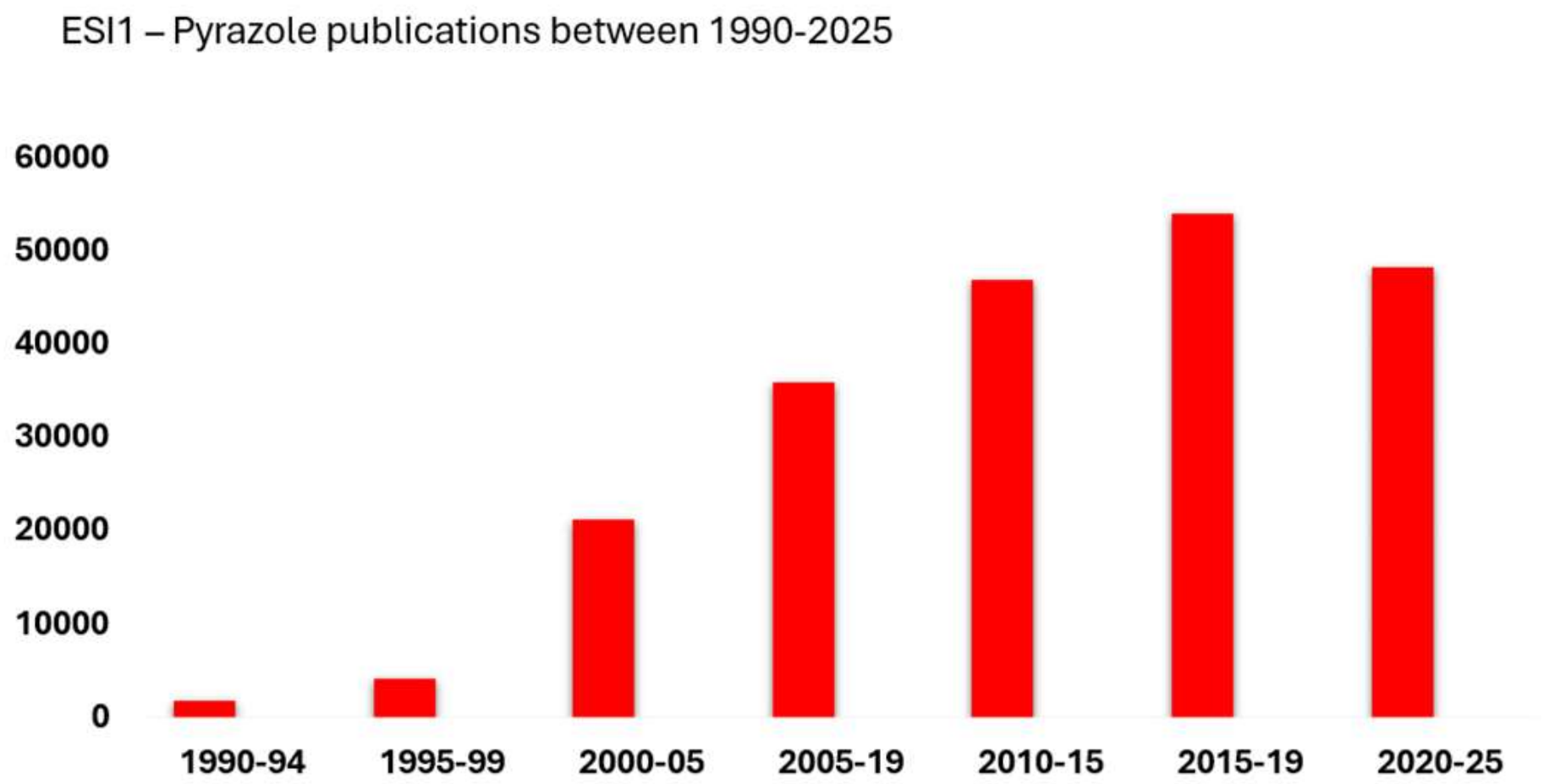

ESI 2 Commercially available starting materials

Reagent supplier 1: MERCK

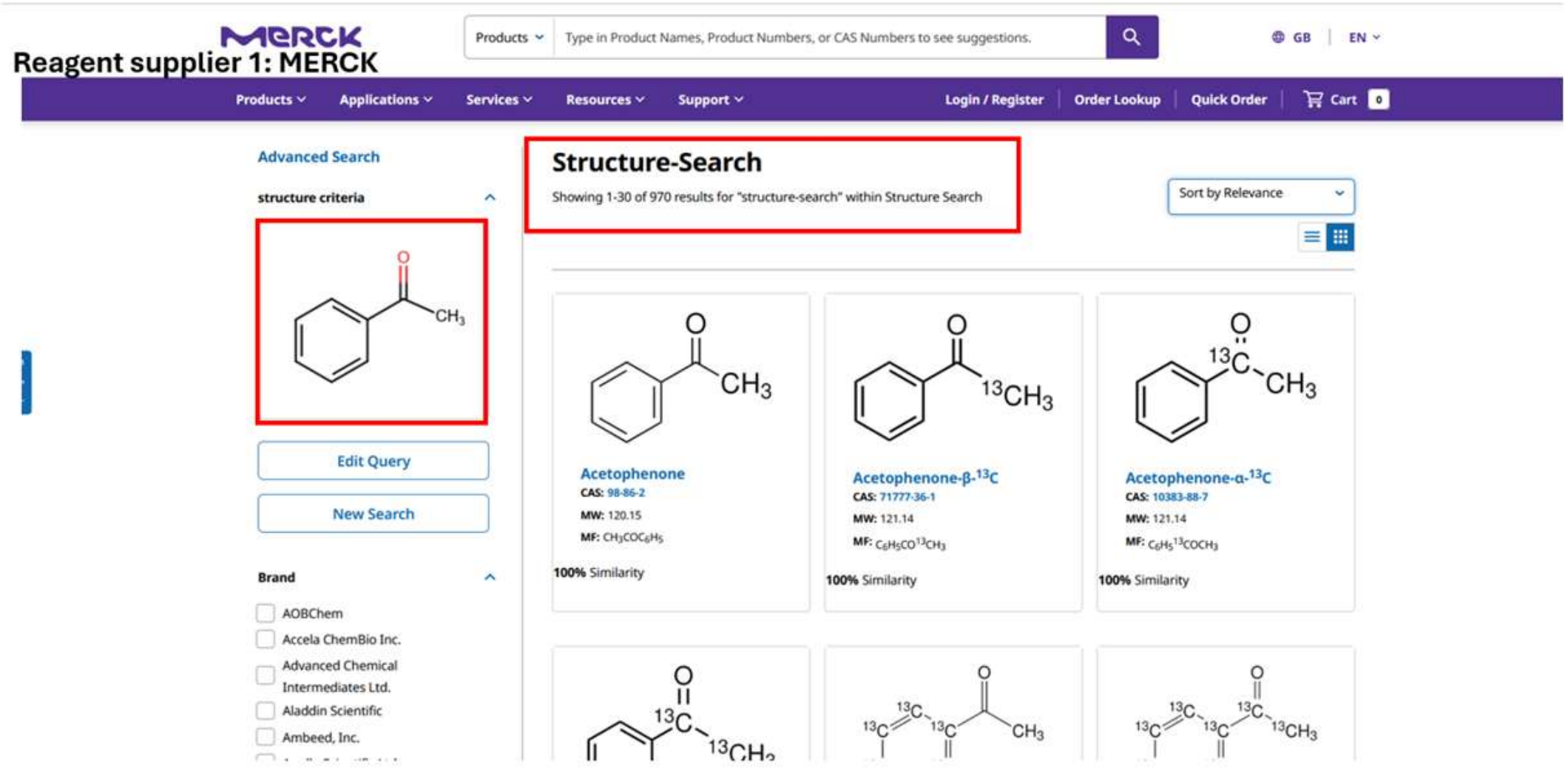

Reagent supplier 1: MERCK

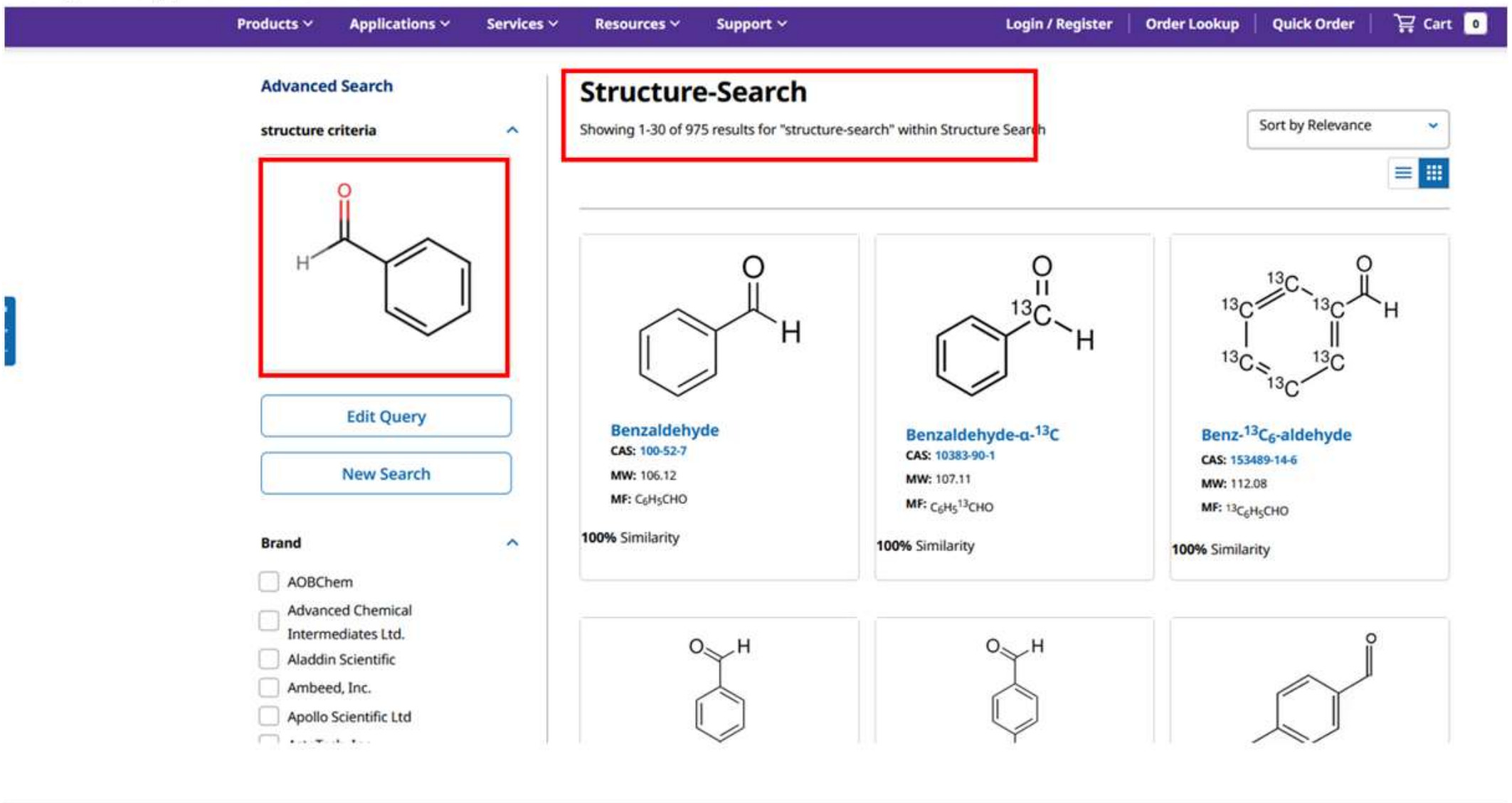

Reagent supplier 2: TCI

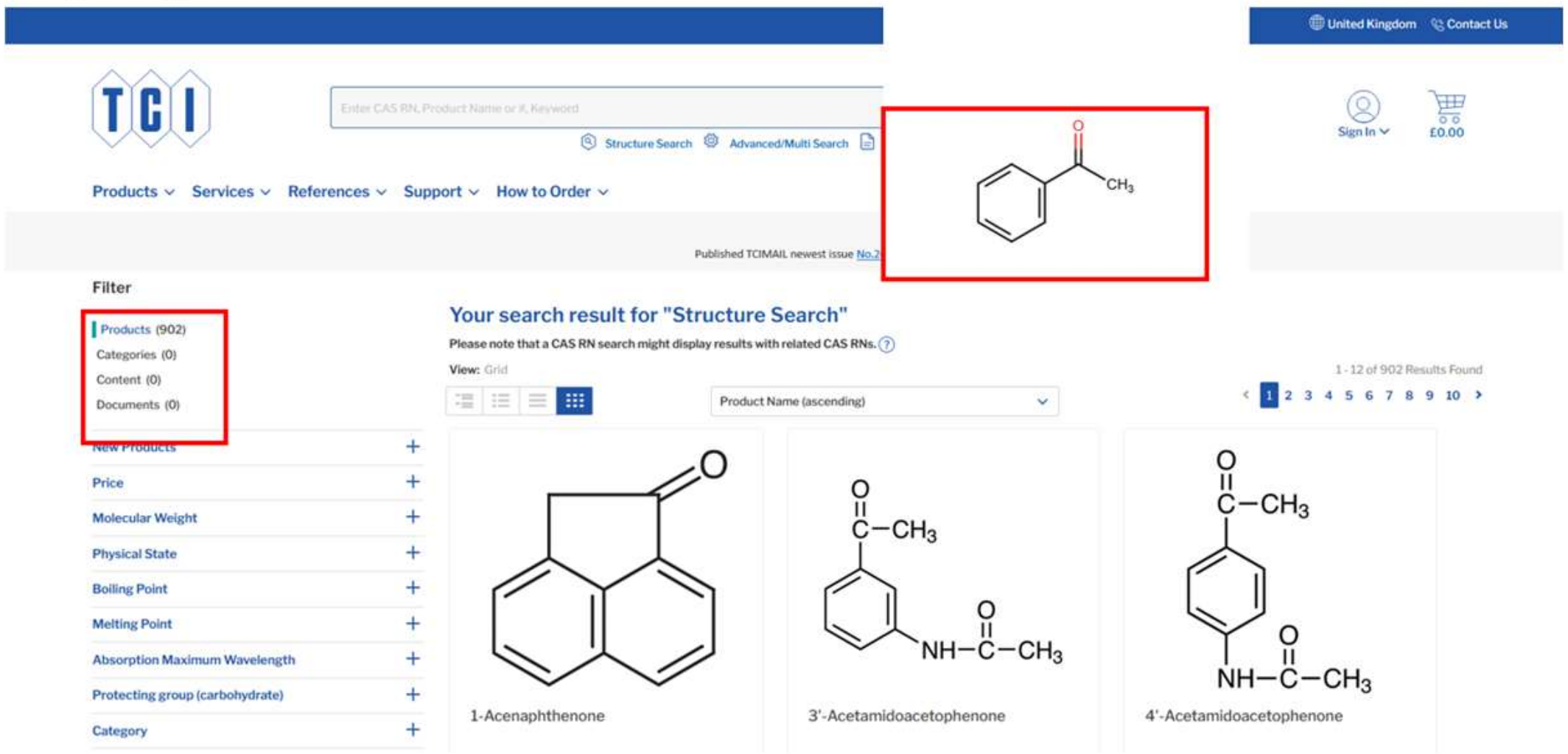

Reagent supplier 2: TCI

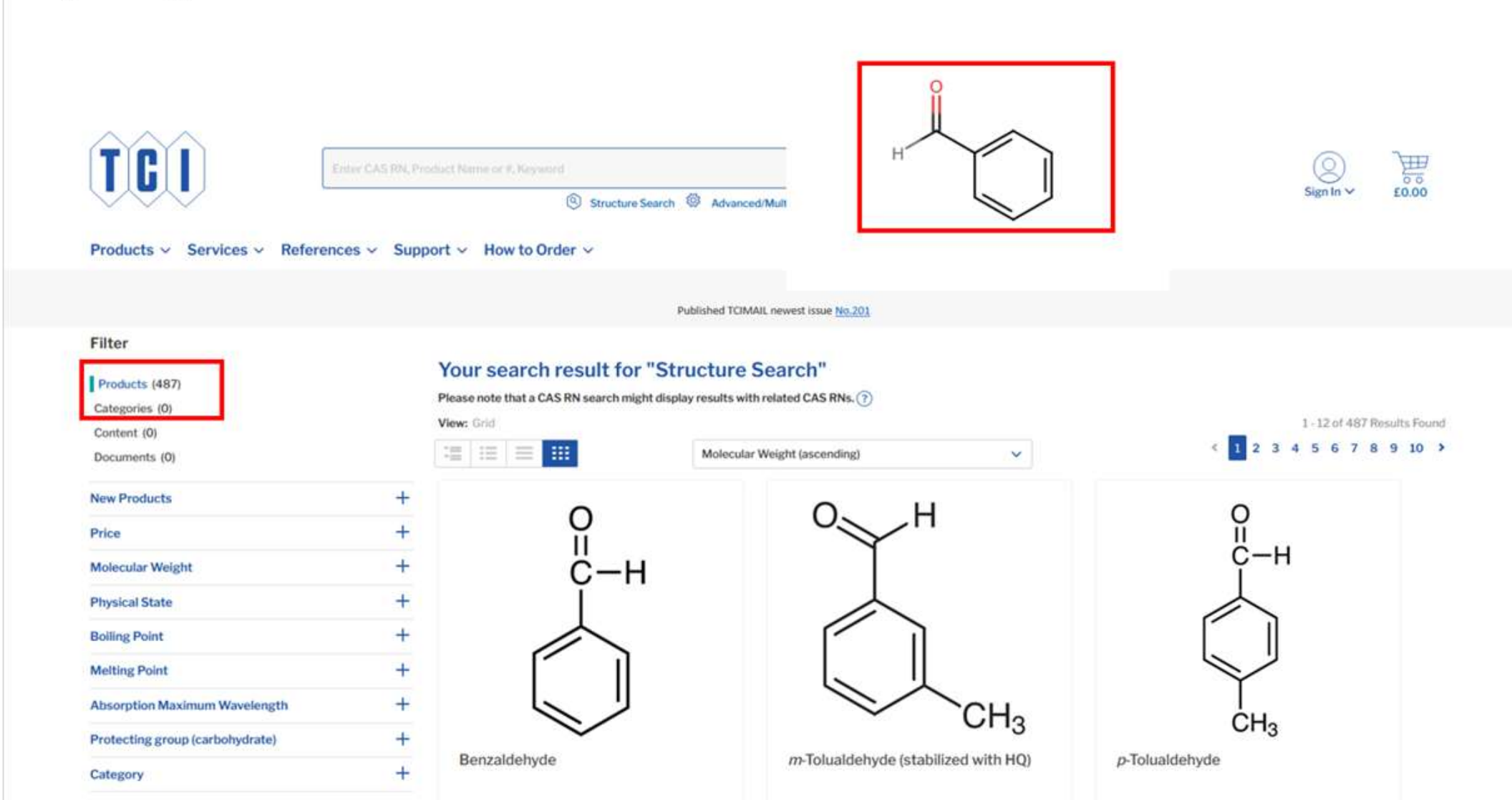

Small selection of commercially available hydrazines (more are available):

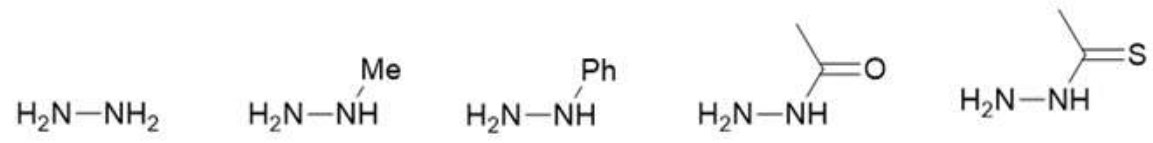

Calculations:

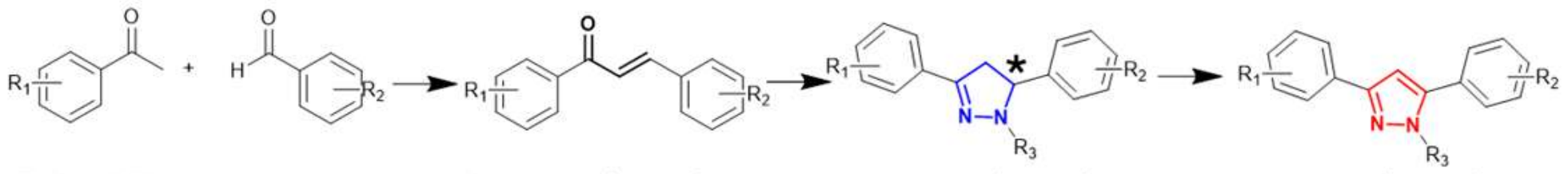

MERCK: 970
TCI: 902
Average: 936

MERCK: 975
TCI: 487
Average: 731

Conservative estimate:
936 x 731 = 684,216
CHALCONES

Conservative estimate:
684,216 x 5 = 3,421,080
PYRAZOLINES

Conservative estimate:
3,421,080
PYRAZOLES

Conservative estimate of combined libraries:
684,216 + 3,421,080 + 3,421,080
= 7,526,376 unique compounds

Supplier data taken on 7th May 2026
These are conversative estimates using commercially available reagents, the use of custom synthesised reagents will result in this potential library being considerably larger.
Pyrazolines are racemic due to a stereogenic centre on position 5 of the ring, enantiomer purification will double the Pyrazoline library (not included in estimates in the manuscript)

5,140 reported chalcones in the literature

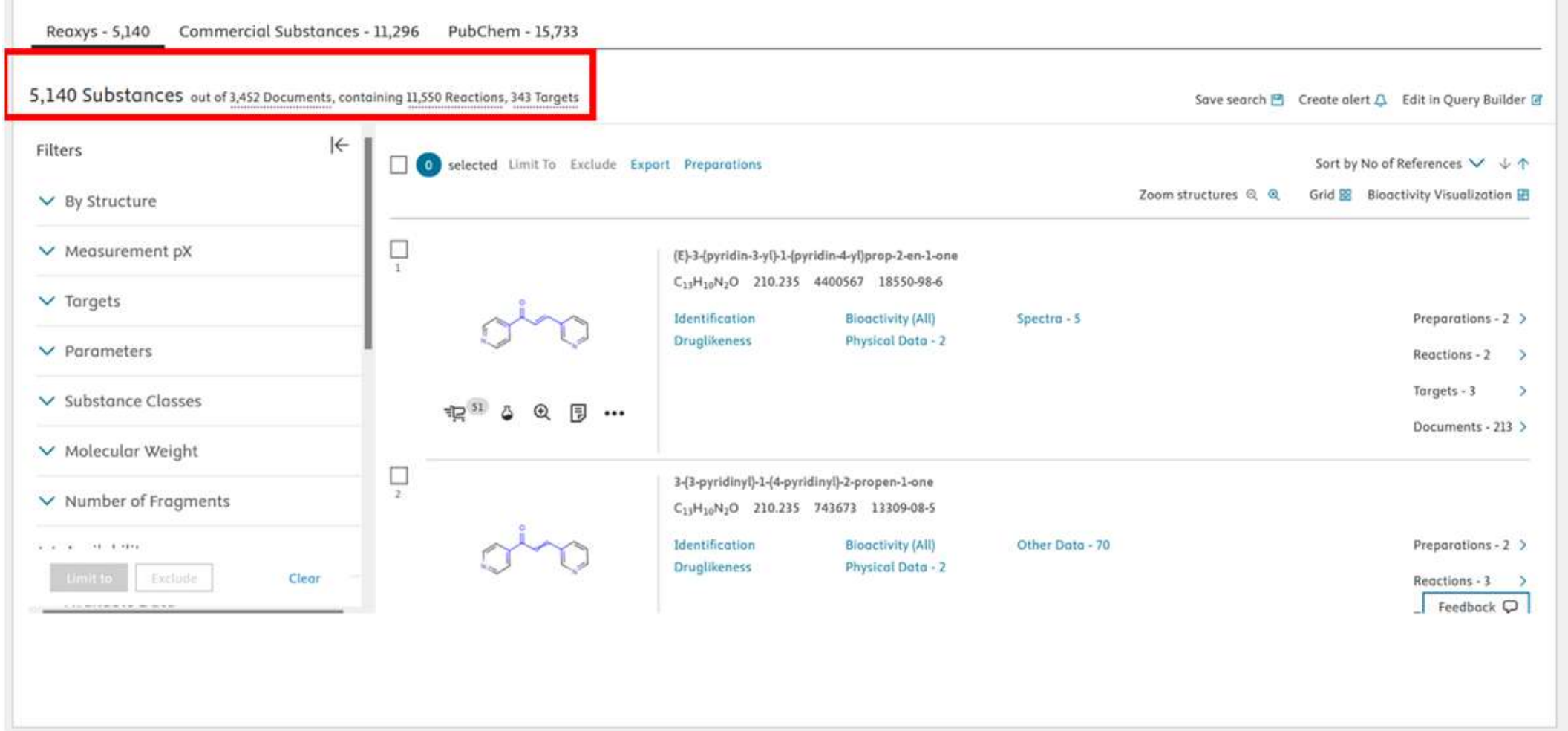

ESI 3 Literature Search

The Scopus database was the primary source of literature for this work, the search terms used for each section are below alongside the total documents found in Red. A graph of the publications per year and authors is also provided. The examples in the manuscript were selected as representative examples of each scaffold and its application using the literature search.

Search terms: Chalcone / anti-cancer / anti-proliferative / article / 1990 -2025 **486 Documents**

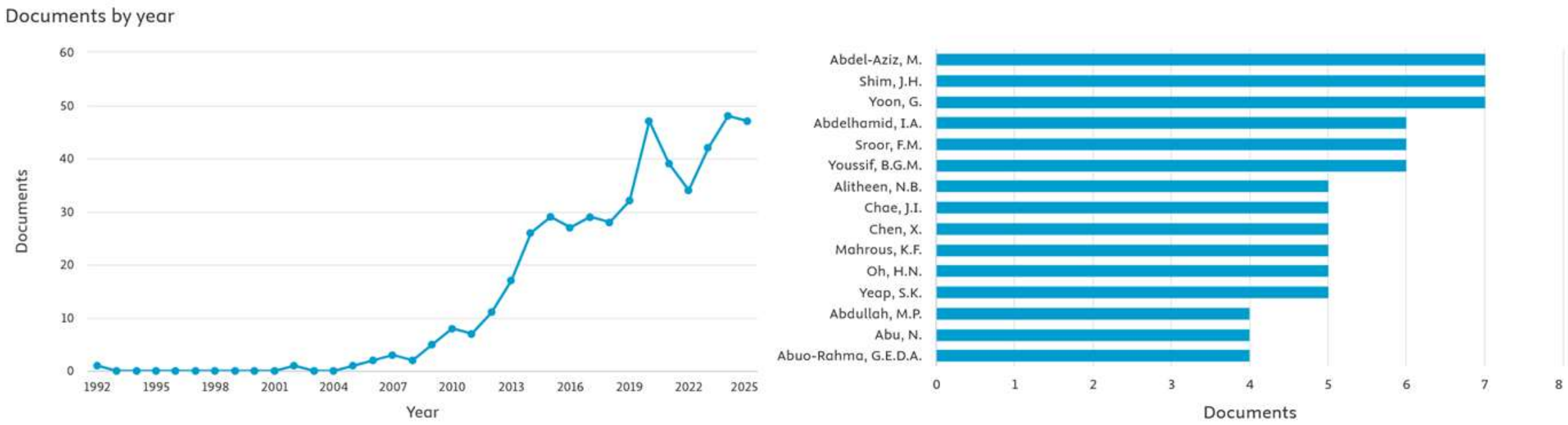

Search terms: Chalcone / anti-inflammatory/ article / 1990 -2025 **1100 Documents**

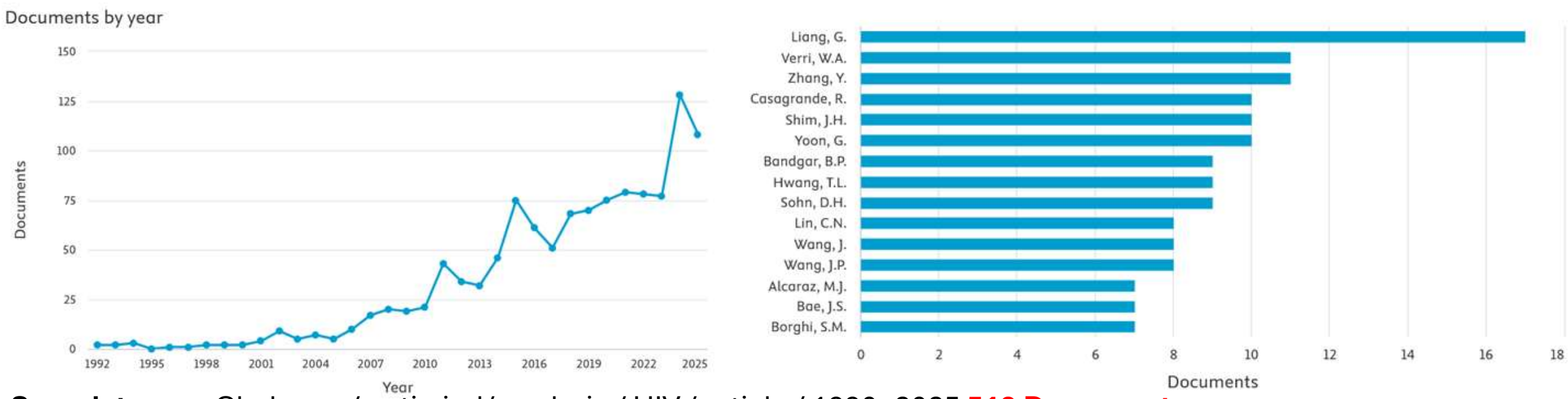

Search terms: Chalcone / anti-viral/ malaria / HIV / article / 1990 -2025 **512 Documents**

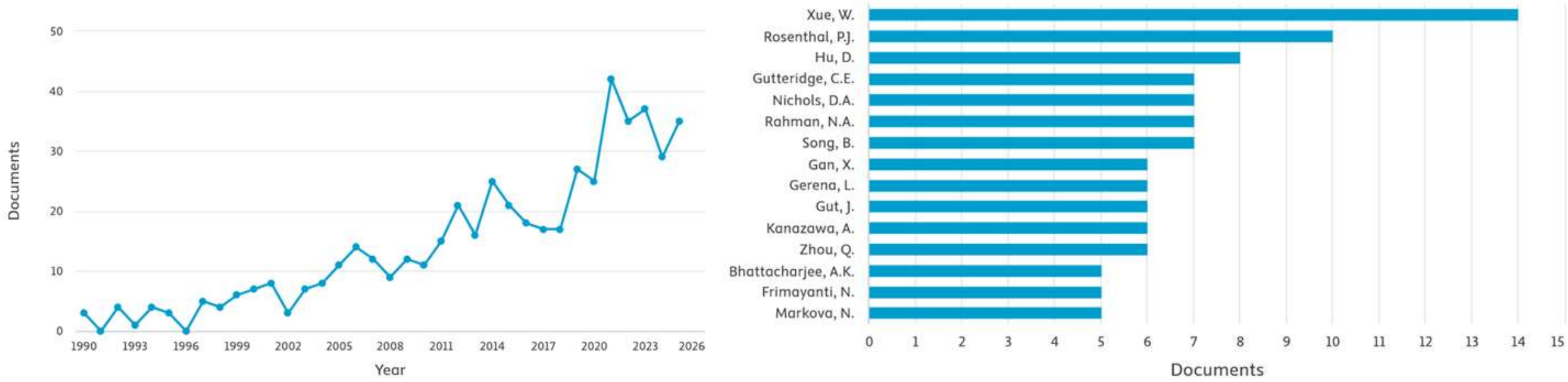

Search terms: pyrazoline / anti-cancer/ anti-proliferative article / 1990 -2025 **190 Documents**

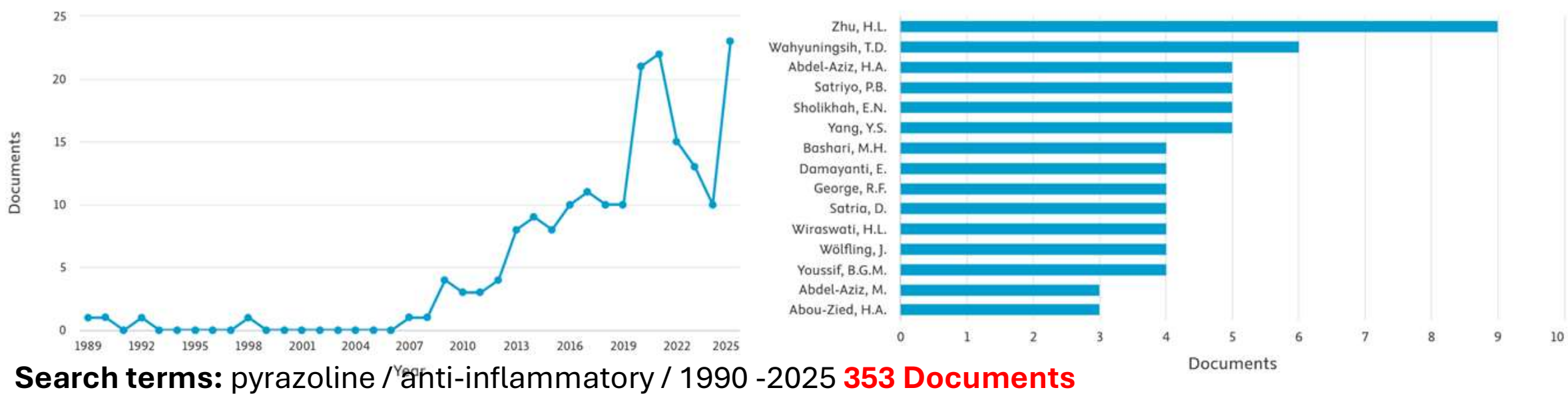

Search terms: pyrazoline / anti-inflammatory / 1990 -2025 **353 Documents**

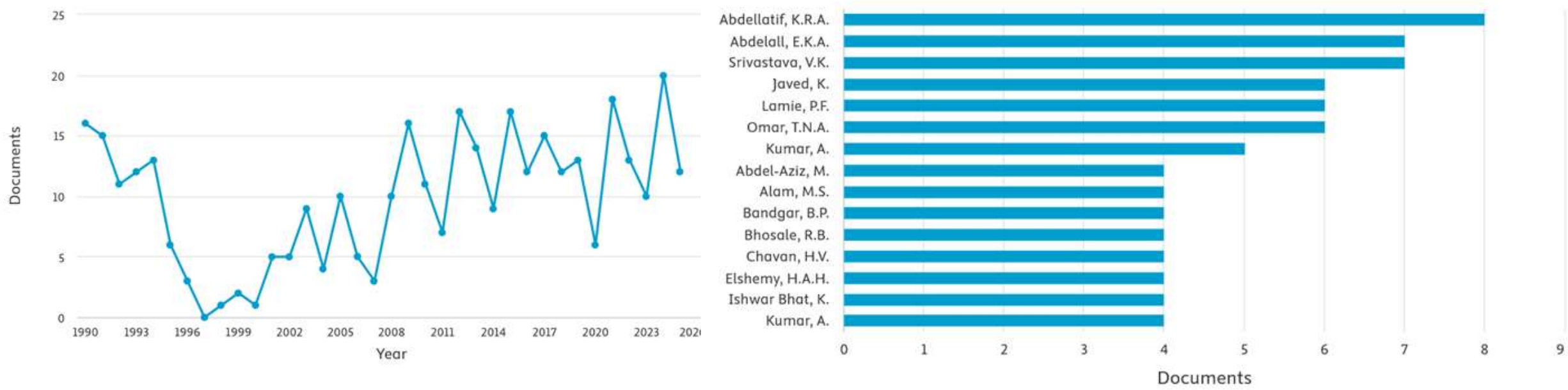

Search terms: pyrazoline / Anti-viral HIV/ Malaria / HIV 1990 -2025 **54 Documents**

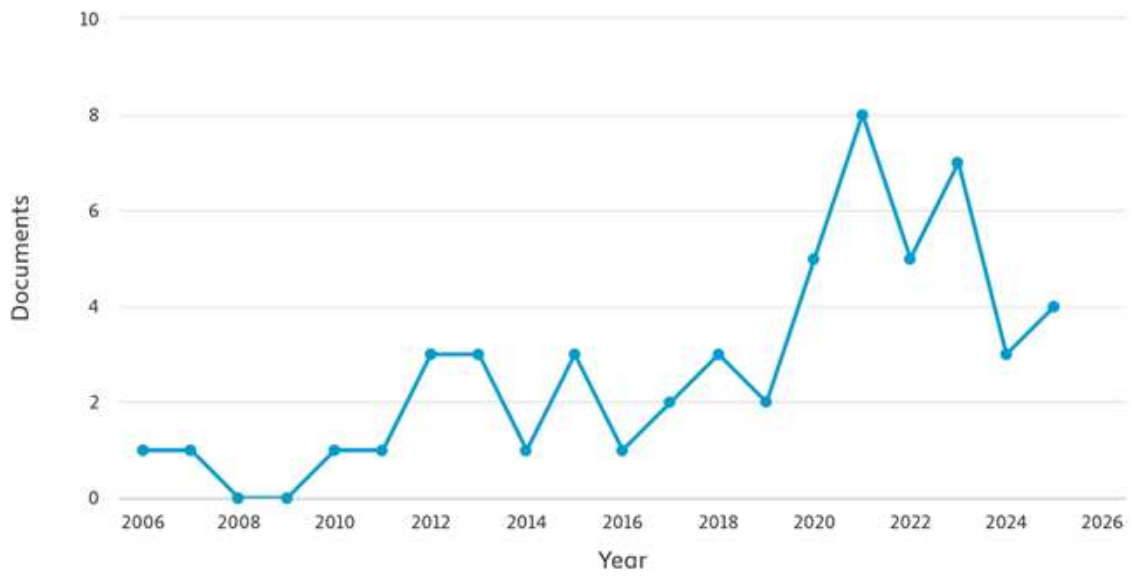

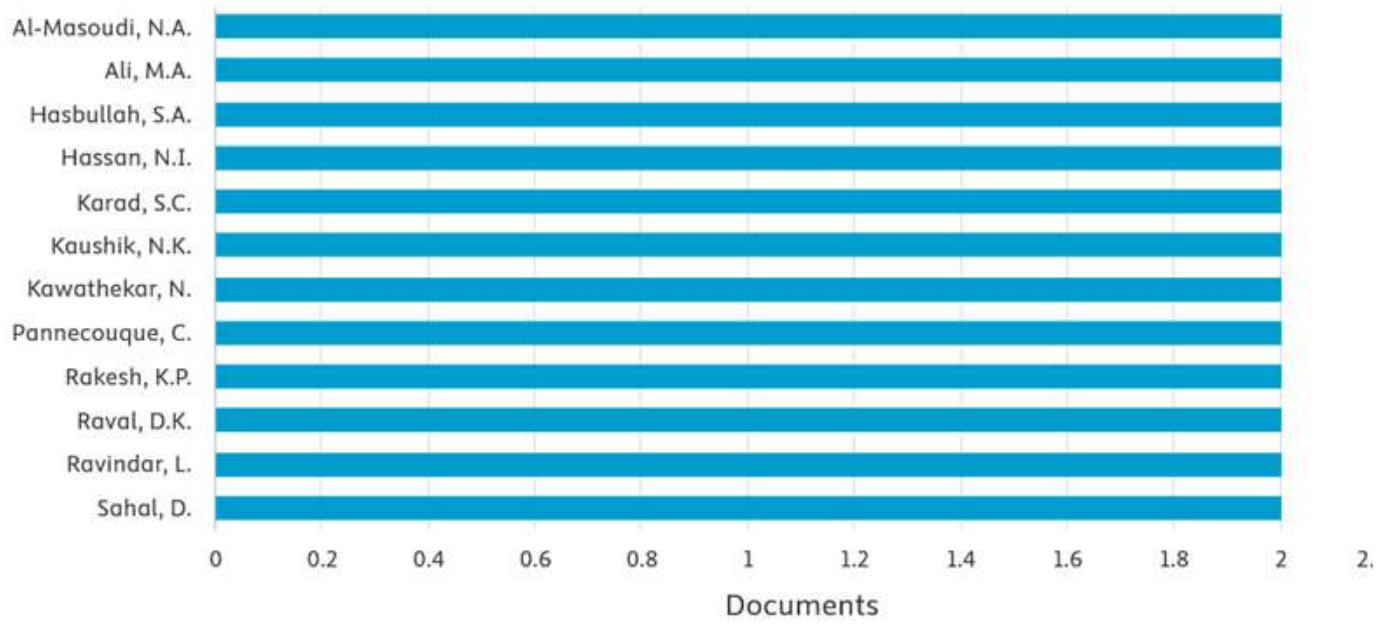

Search terms: Pyrazole / anti-cancer / anti-proliferative / article / 1990 -2025 **690 Documents**

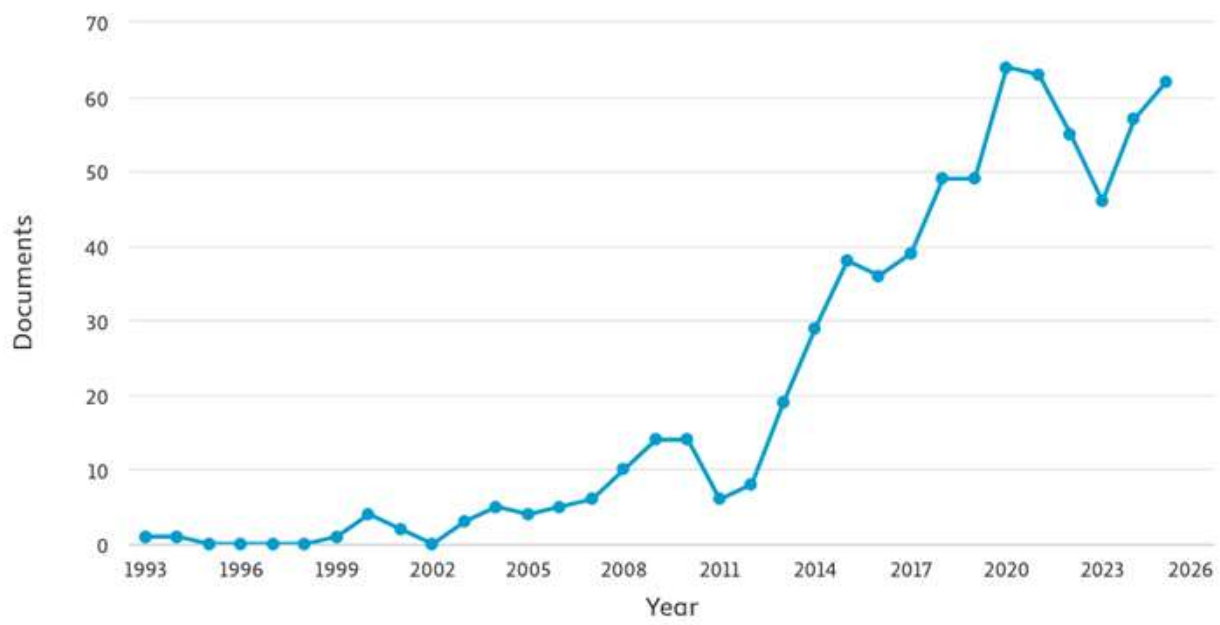

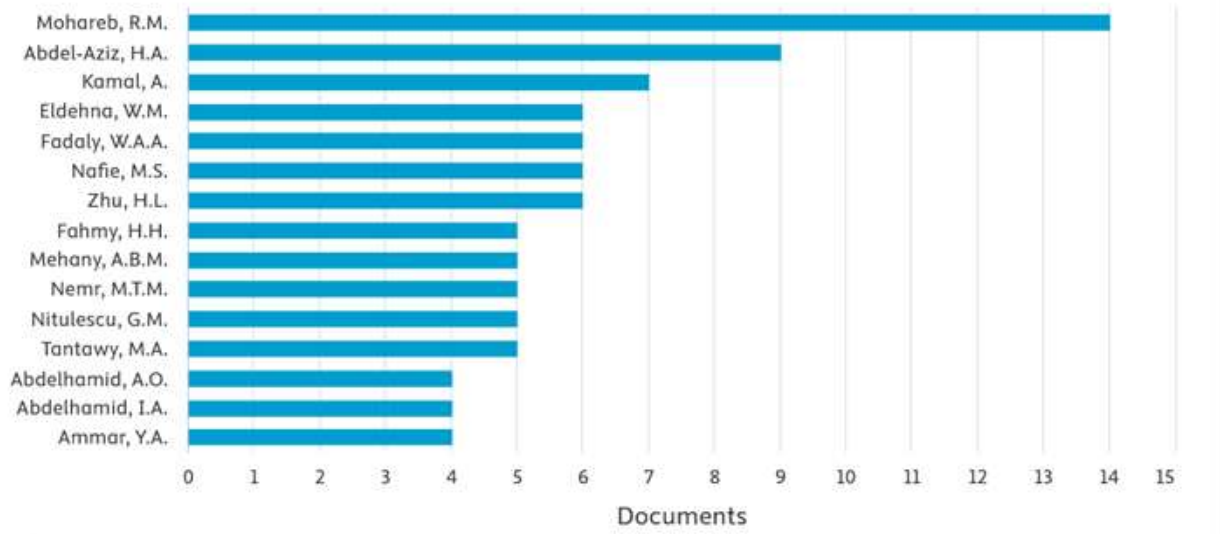

Search terms: Pyrazole / anti-inflammatory/ article / 1990 -2025 **2356 Documents**

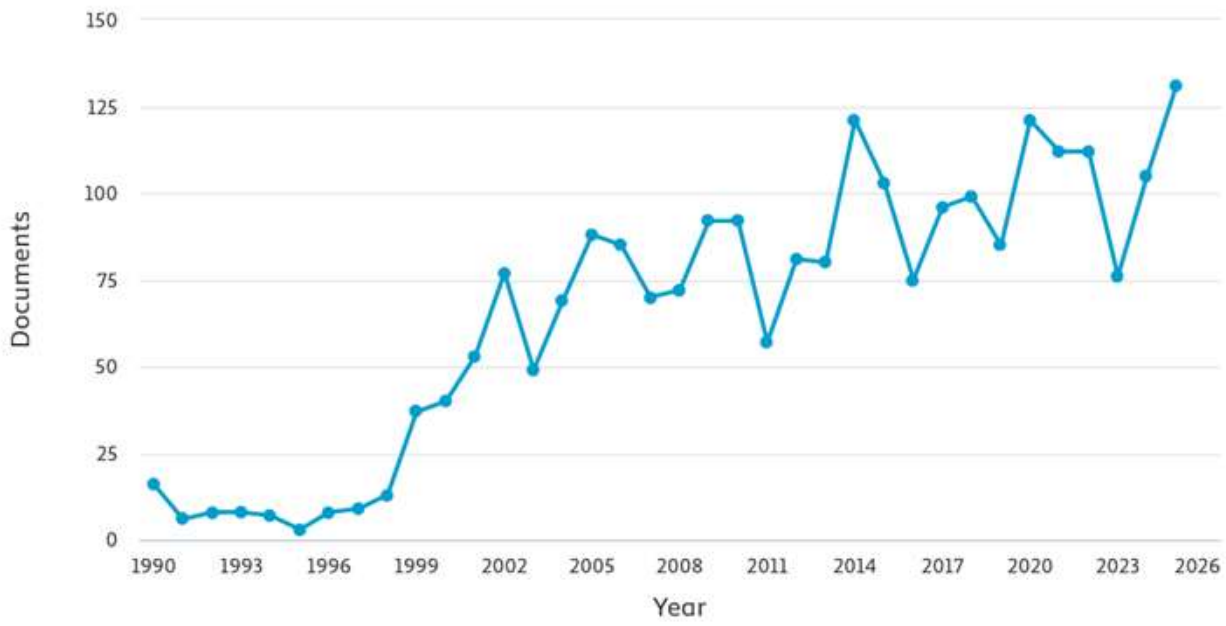

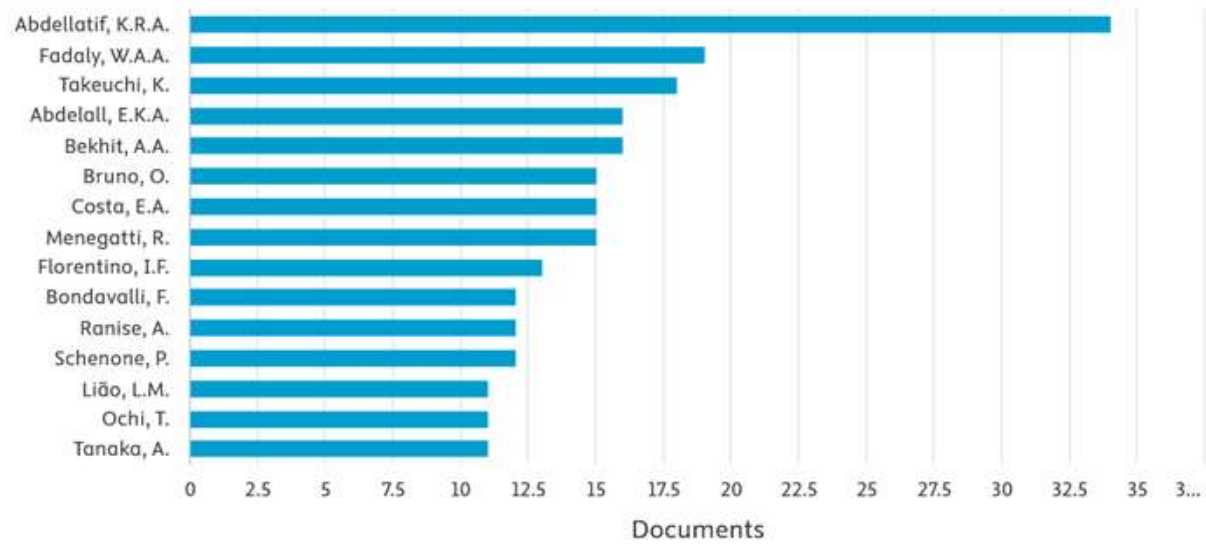

Search terms: Pyrazole / anti-viral/ HIV/ malaria article / 1990 -2025 **512 Documents**

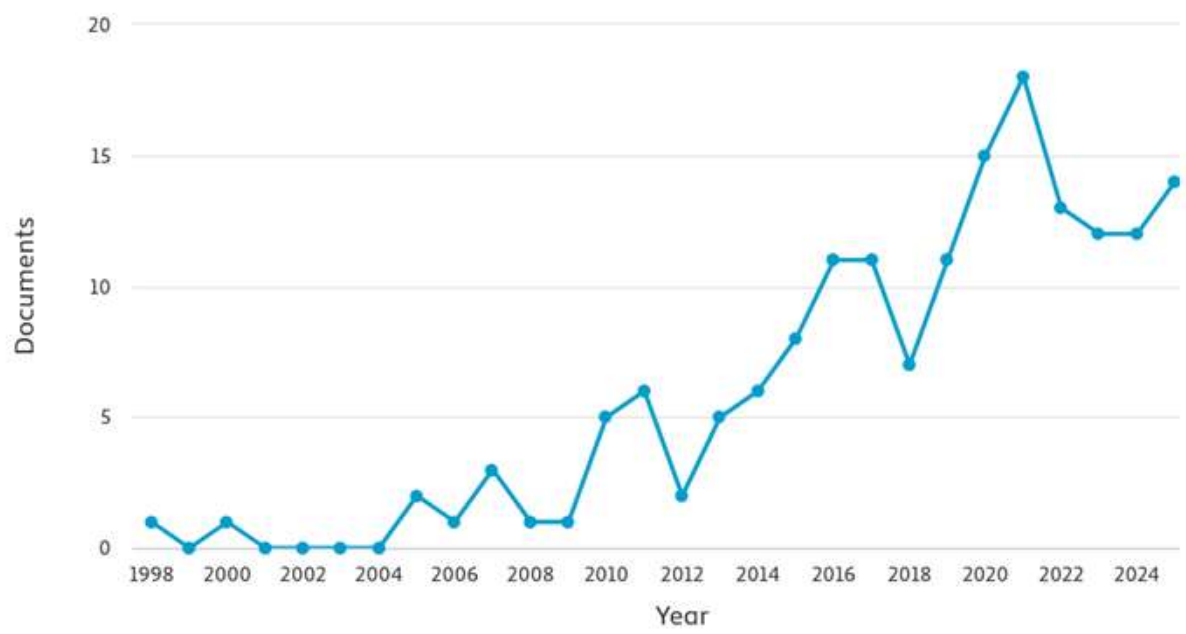

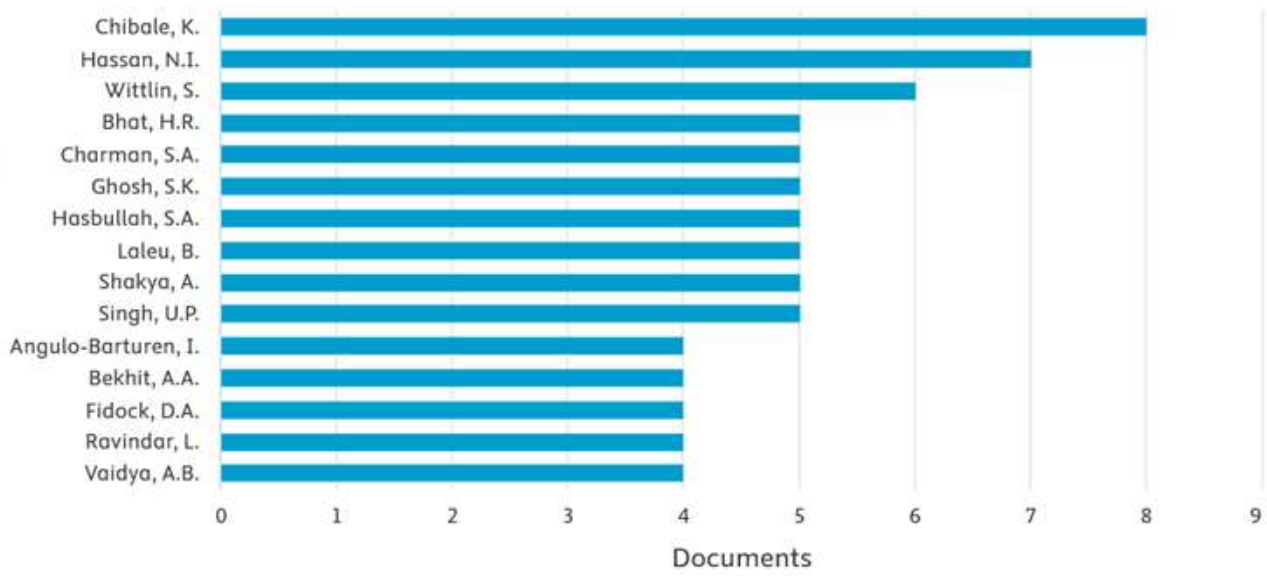

Search terms: Green chemistry / chalcone/ pyrazoline / pyrazole 1990 -2025 **821 Documents**

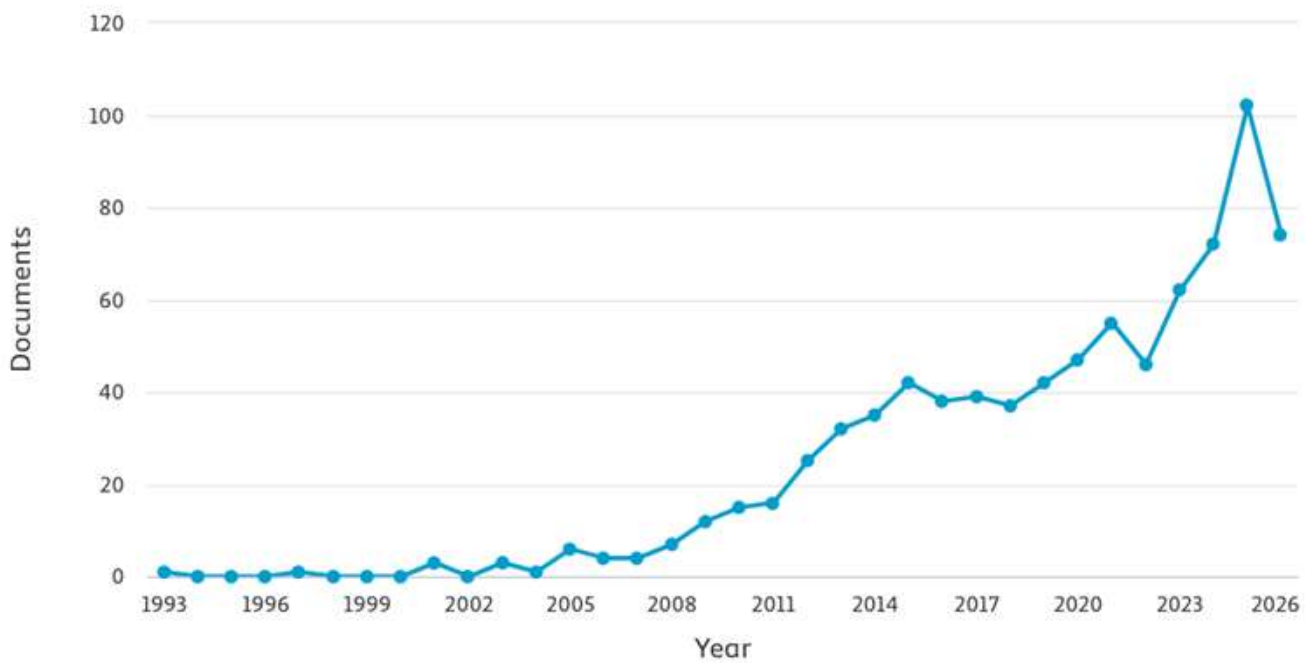

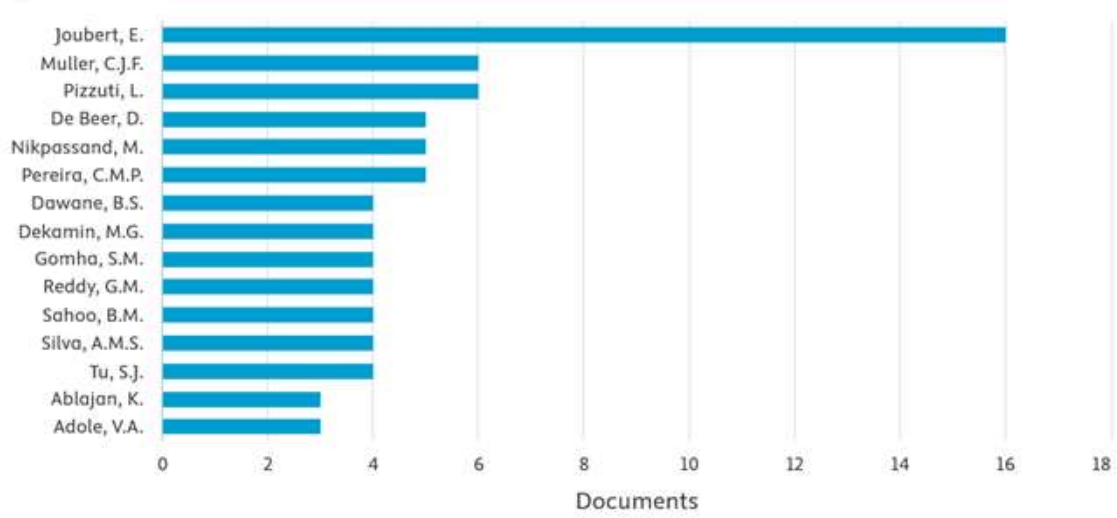

ESI 4 Table of examples used in the manuscript:

Lead compound	IC_{50}	Mode of action	No compounds	Reference
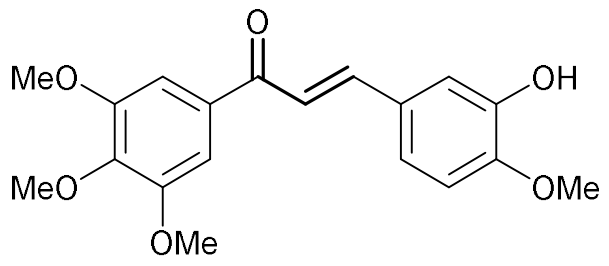	4.3 nM	Tubulin binder	8	*Bioorg. Med. Chem. Lett.*, 1998, **8**, 1051.
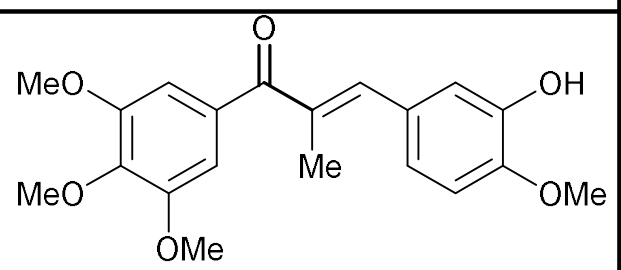	0.21 nM	Tubulin binder	97	*Bioorg. Med. Chem.*, 2009, **17**, 7698.
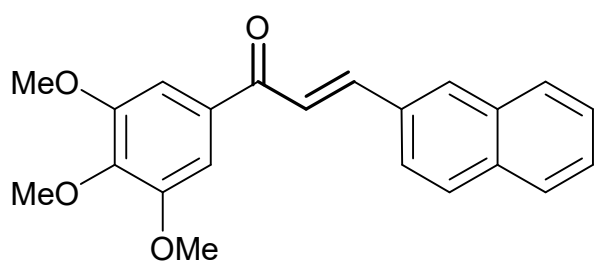	0.7 μM	Tubulin binder	5	*Eur. J. Med. Chem*. 2013, **63**, 501
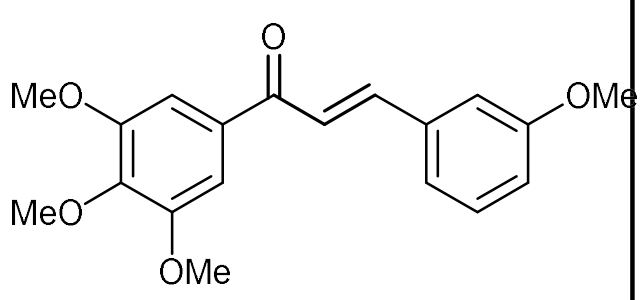	1.8 μM	Nitric oxide	23	*Bioorg. Med. Chem.*, 2009, **17**, 7909.
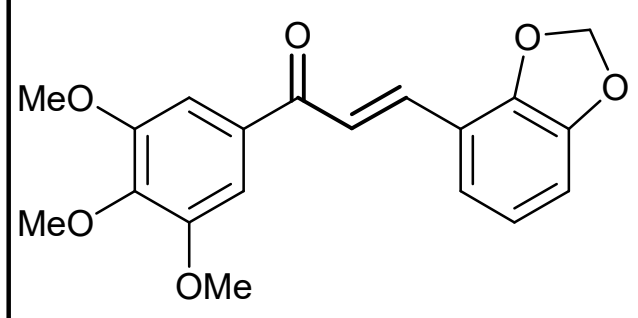	2.9 μM	EGFR	33	*J. Mol. Struct.*, 2023, **1275**, 134572.
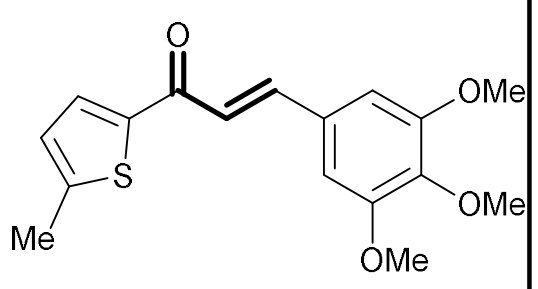	5.5 μM	Tubulin binder	23	*Eur. J. Med. Chem.*, 2019, **184**, 111752.
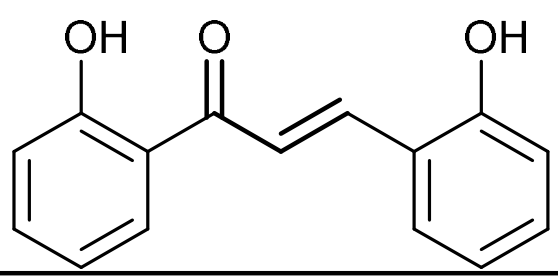	100 μM	Mitochondrial membrane	10	*Chem. Biol. Interact.*, 2004, **148**, 57.
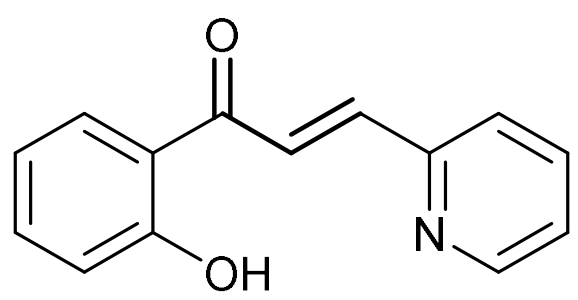	13.8 μM	DNA Damage	41	*Bioorg. Med. Chem.*, 2007, **15**, 3356.
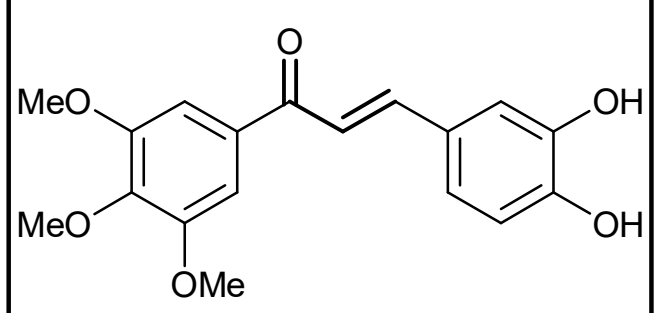	1.9 μM	NK-κB	34	*J. Med. Chem.*, 2009, **52**, 7228.
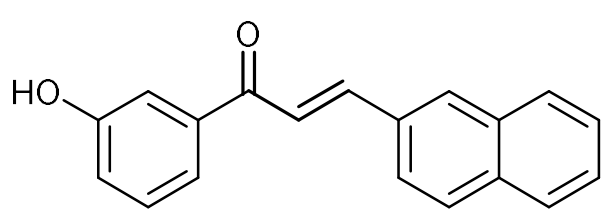	1.44 μM	GSH and ATP	30	*J. Med. Chem.*, 2012, **55**, 1382.
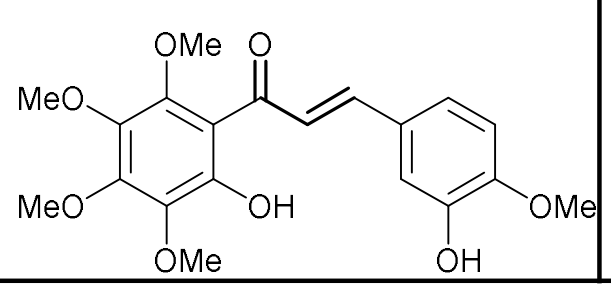	34 μM	Not determined	14	*Chem. Nat. Compd.*, 2019, **55**, 11.

Lead compound	IC_{50}	Mode of action	No of compound reported	Reference
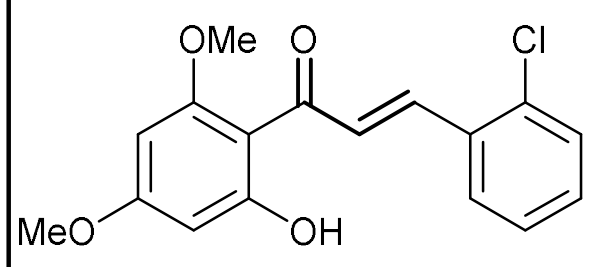	25 µM	Nrf2 and GSH	12	*Int. J. Mol. Sci.*, 2023, **24**, 10667
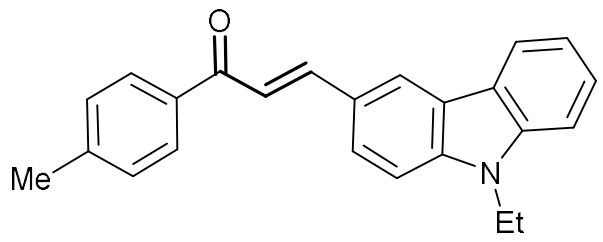	0.22 µM	Topoisomerase II	13	*Eur. J. Med. Chem.*, 2018, **145**, 498
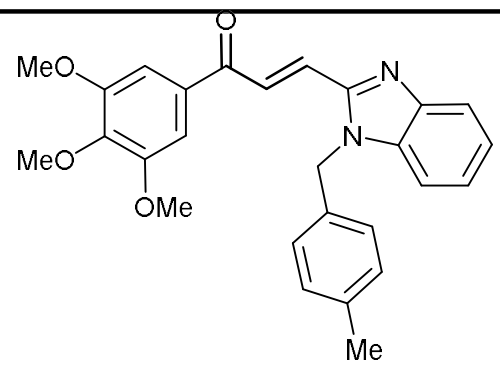	3.8 µM	Topoisomerase II	42	*Molecules*, 2020, **25**, 3180.
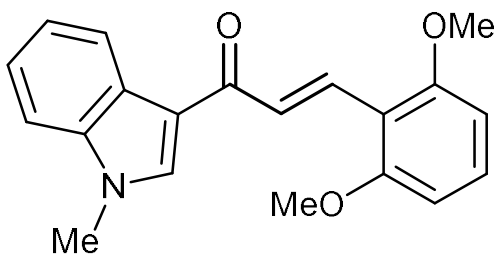	9.27 µM	Breast cancer resistance protein	44	*J. Med. Chem.*, 2012, **55**, 3193.
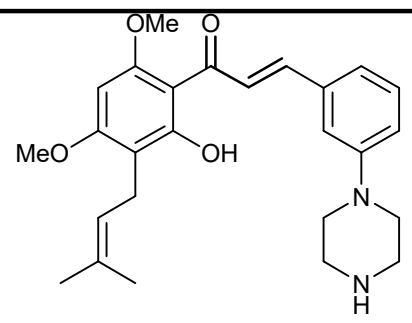	65.7 µM	Not determined	1	*Frontiers in Pharmacology*, 2021,**6**, 653306.
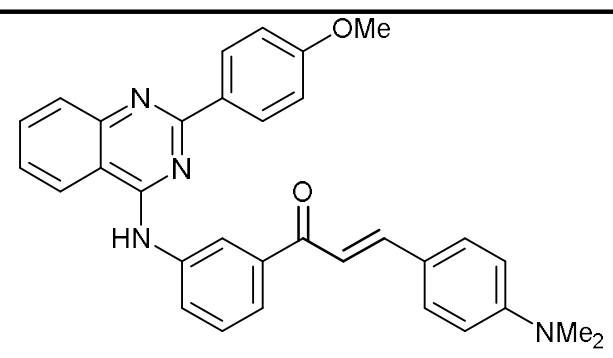	27.8 nM	EGFR	16	*RSC Med. Chem.*, 2026, **17**, 2984
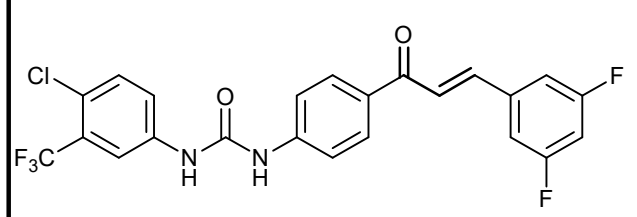	1.3 µM	EGFR	19	*Molecules*, 2025, **30**, 4526.
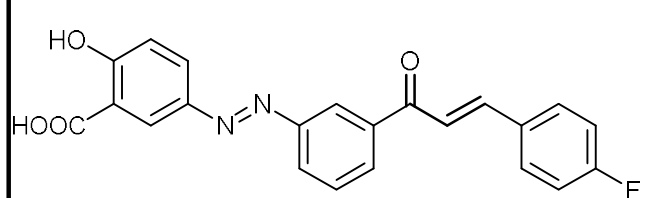	1.2 µM	COX-2	18	*Molecules*, 2022, **27**, 1158
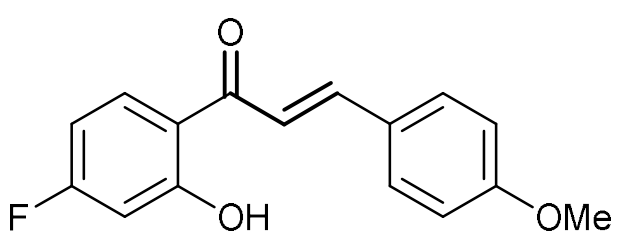	1.53 µM	COX-2	8	*J. Enzyme Inhib. Med. Chem.*, 2015, **30**, 484.
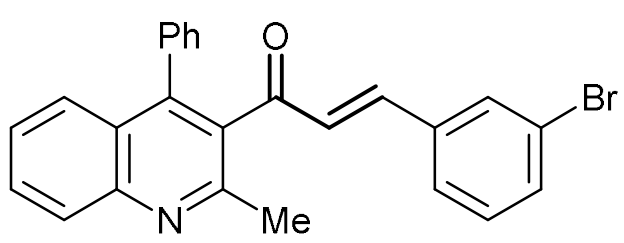	0.19 µM	COX-2	24	*Bioorg. Med. Chem. Lett.*, 2020, **30**, 127173.
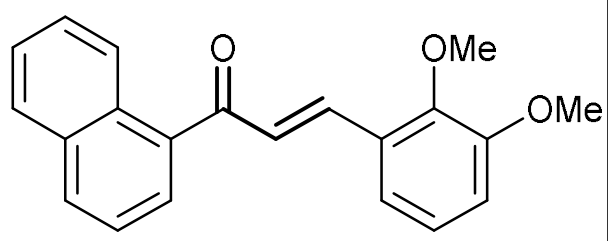	88% inhibition	THF-α and IL-6 cytokine	23	*Drug Des., Dev. Ther.*, 2014, **8**, 1405.

Lead compound	IC_{50}	Mode of action	No of compound reported	Reference
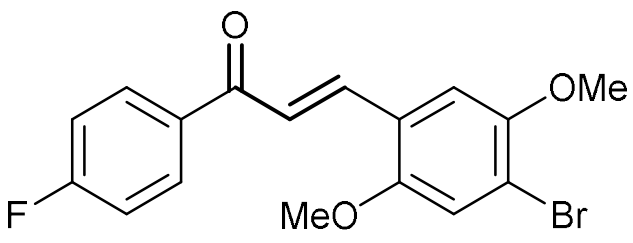	0.25 μM	IL-1β cytokine	37	*ACS Med. Chem. Lett.*, 2022, **13**, 560
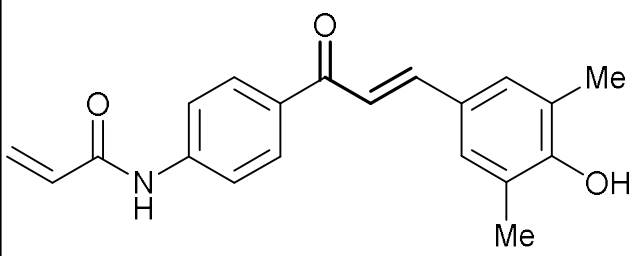	0.19 μM	COX-2	40	*Eur. J. Med. Chem. Rep.* **2023**, 7, 100100
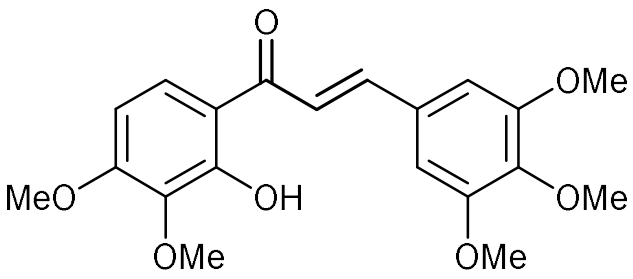	1.1 μM	Nitric oxide	24	*Med. Chem. Res.*, 2015, **24**, 1672.
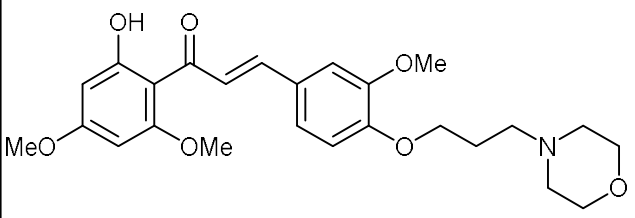	6.4 μM	Nitric oxide	53	*Eur. J. Med. Chem.*, 2020, **193**, 112216
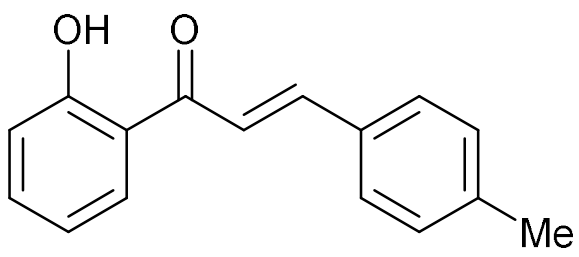	5.25 μM	Lipoxygenase	31	*Bioorg. Med. Chem.*, 2009, **17**, 8073.
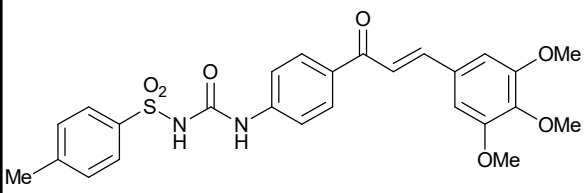	0.45 μM	Lipoxygenase and COX-2	20	*Life Sci.*, 2006, **78**, 2911
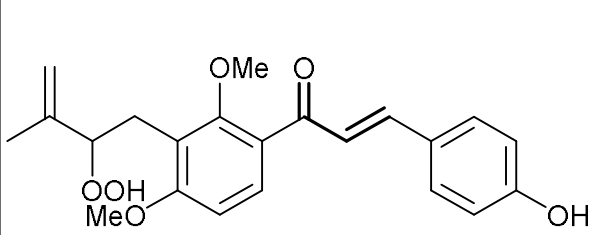	1.2 μM	Papain-like protease	9	*J. Enzyme Inhib. Med. Chem.*, 2016, **31**, 23.
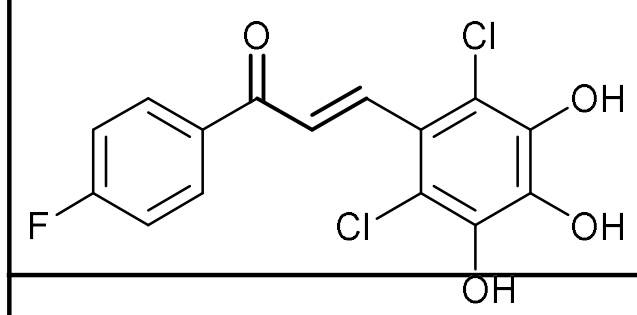	83.2 nM	3-chymotrypsin-like protease	4	Chem. *Biol. Drug Des.*, 2019, **94**, 2023.
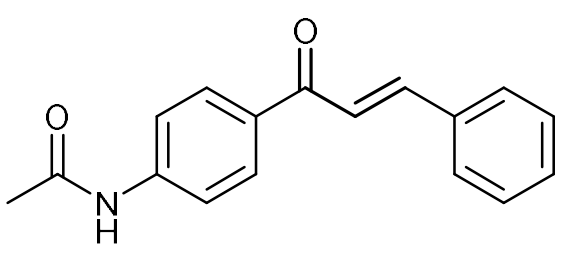	*In silico* study	Main protein (Mpro)	6	*Biochem. Biophys. Res. Commun.* 2021, **537**, 71.
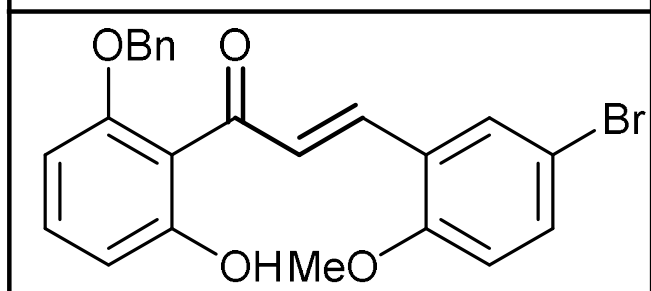	86% inhibiti on of HIV-1	HIV integrase	32	*Bioorg. Med. Chem.*, 2016, **24**, 2768.
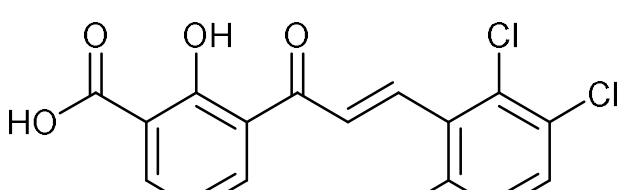	3.7 μM	HIV integrase	63	*Bioorg. Med. Chem.*,

Lead compound	IC_{50}	Mode of action	No of compound reported	Reference
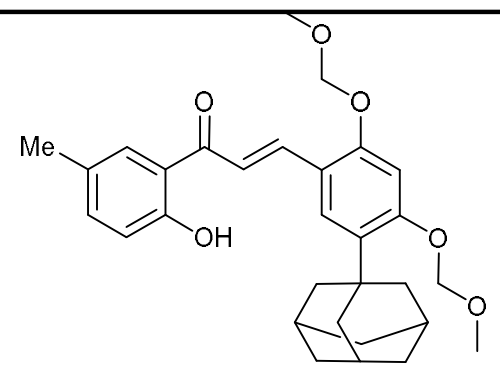	HIV Provirus	Human transcriptional elongation factor	14	*Sci. Rep*. 2017, **7**, 10657.
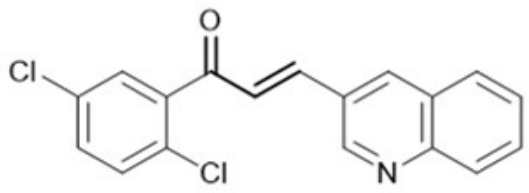	0.23 μM	Trophozoite cysteine	29	*J. Med. Chem*., 1995, **38**, 5031.
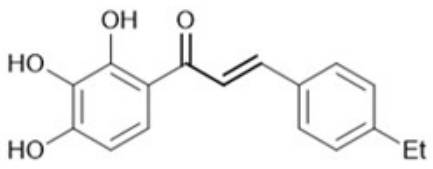	2.42 μM	Not determined	102	*Bioorg. Med. Chem*., 2003, **11**, 2729.
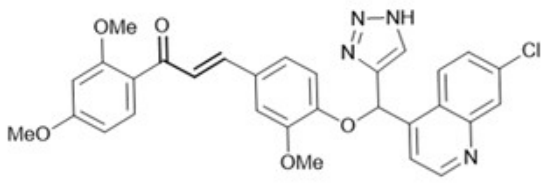	0.09 μM	B hematin	46	*Bioorg. Med. Chem*., 2010, **18**, 8243
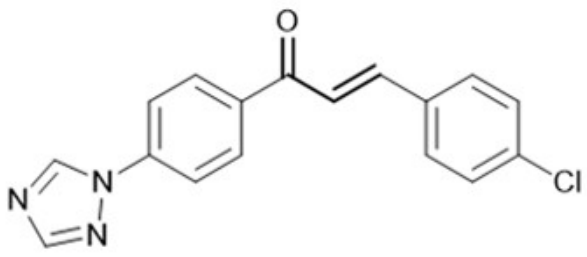	1.52 μg/mL	Cysteine protease	14	*Eur. J. Med. Chem*., 2008, **43**, 1530.

Lead compound	IC_{50}	Mode of action	No of compound reported	Reference
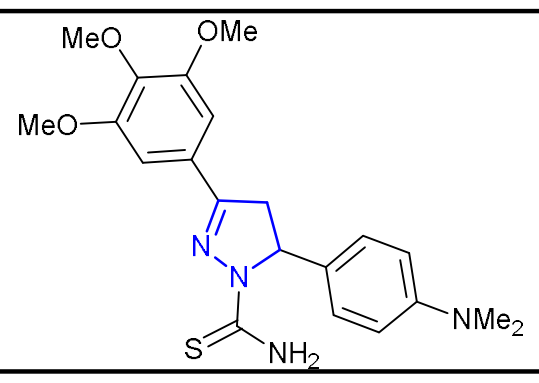	6.9 µM	Tubulin destabiliser	8	*Arch. Pharm.*, 2011, **344**, 572.
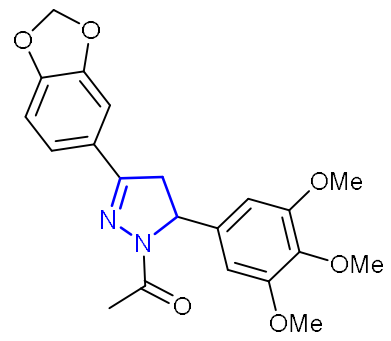	1.4 µM	Not determined	8	*Arch. Pharm.*, 2011, **344**, 572.
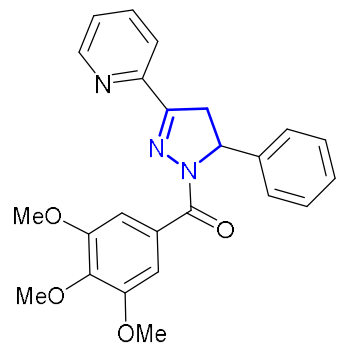	0.69 µM	Tubulin destabiliser	12	*MedChem Comm*, 2013, **4**, 956.
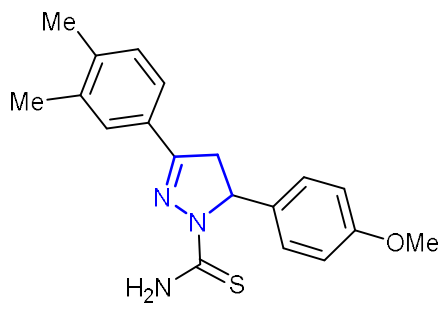	80 nM	EGFR	23	*Bioorg. Med. Chem.*, 2010, **18**, 4606.
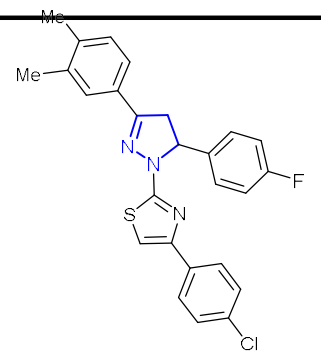	70 nM	EGFR	23	*Bioorg. Med. Chem.*, 2010, **18**, 4606.
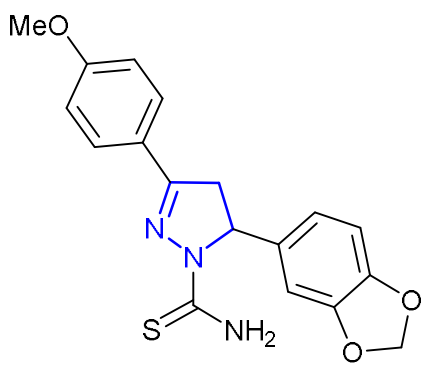	1.88 µM	Not determined	18	*Arch. Pharm. Chem. Life Sci.*, 2012, **345**, 275.
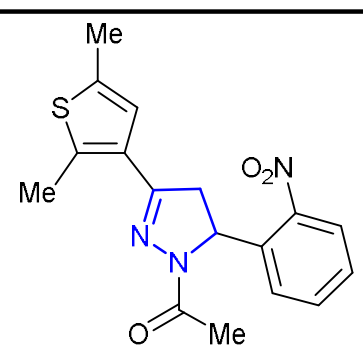	3.1 µM	Topoisomerase II	29	*RSC Adv.*, 2016, **6**, 14880
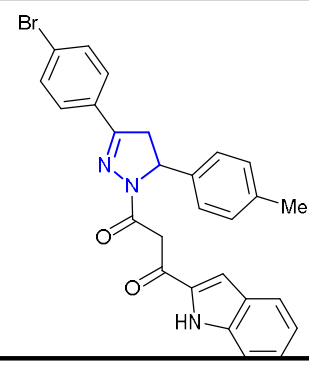	3.2 µM	Topoisomerase II	12	*Drug Dev. Res.*, 2022, **83**, 1555.
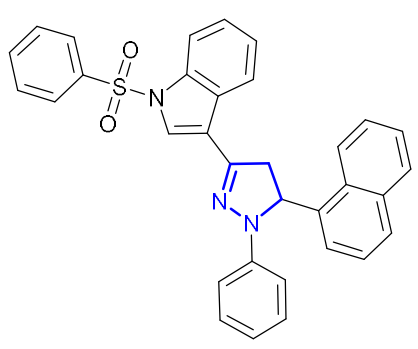	3.2 µM	Topoisomerase I	20	*Bioorg. Med. Chem. Lett.*, 2020, **30**, 126925.
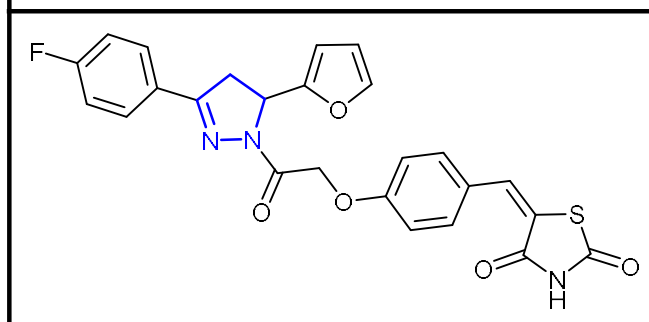	18.8 µM	Multi-target: EGFR-2 and HDAC	19	*RSC Med. Chem.*, 2021, **12**, 1540.

Lead compound	IC_{50}	Mode of action	No of compound reported	Reference
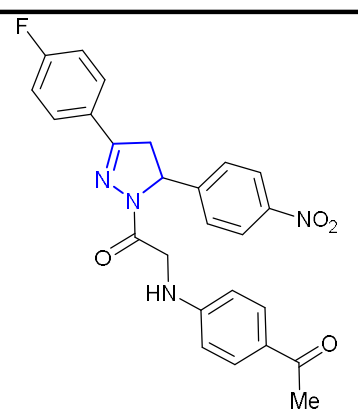	10 μM	COX-2	18	*Bioorg. Med. Chem.*, 2016, **24**, 2032.
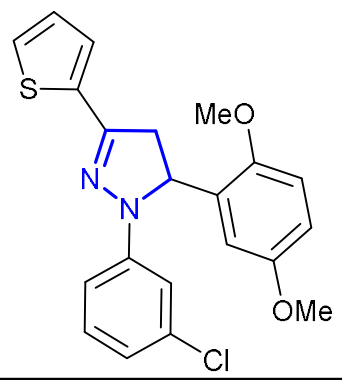	10 μM	COX-2	18	*Bioorg. Med. Chem.*, 2016, **24**, 2032.
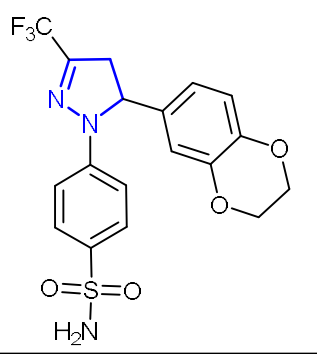	19.8 μM	COX-2	24	*Bioorg. Med. Chem.*, 2014, **22**, 4109.
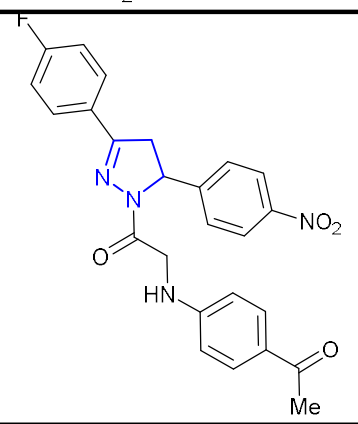	12.1 μM	COX-2	20	*Bioorg. Med. Chem.*, 2008, **16**, 3907.
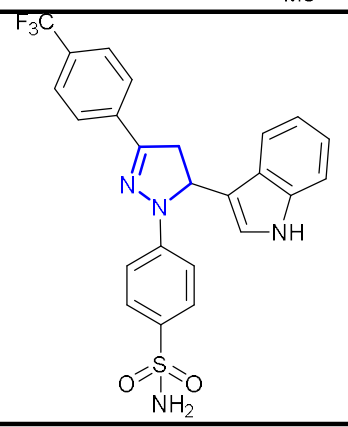	1.4 μM	COX-2	34	*ChemBioChem*, 2023, **24**, e202300116
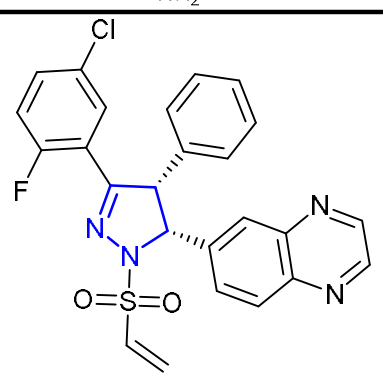	2.5 nM	Coronavirus main protease	21	*ChemBioChem*, 2023, **24**, e202300116
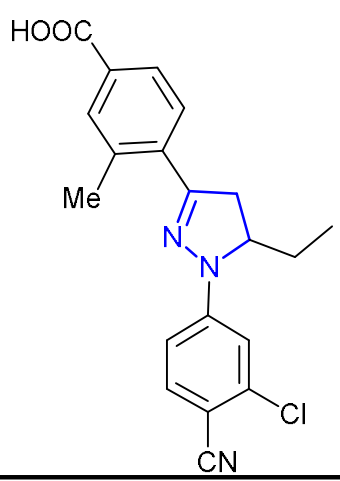	6.6	NSP14 MTase	35	*ACS Med. Chem. Lett.*, 2025, **16**, 1918
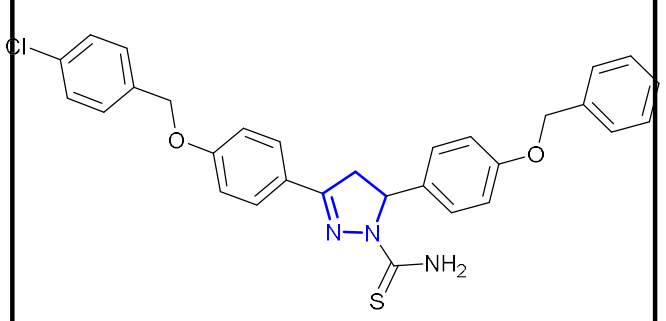	*In silico*	SARS-CoV-2 main protease	3	*Res. Chem. Intermed.*, 2022, **48**, 4729.
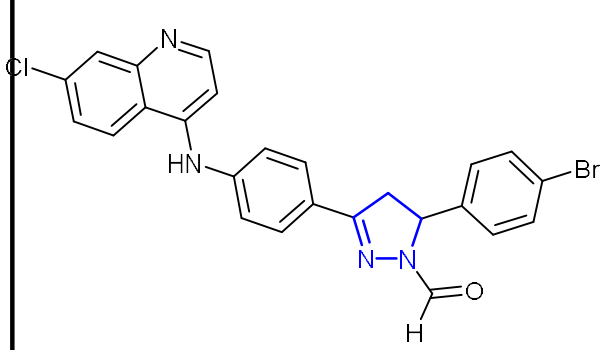	14.1 μg/mL	*P. Falciparum*	12	*Sci. Rep.*, 2025, **15**, 767.

Lead compound	IC_{50}	Mode of action	No of compound reported	Reference
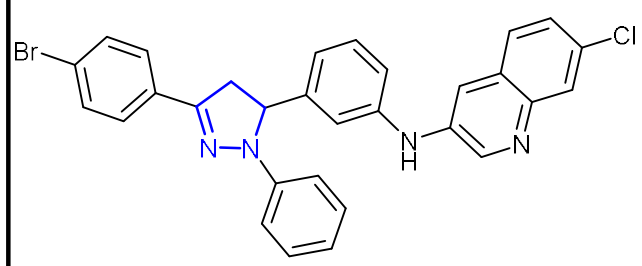	5.54 µg/mL	*P. Falciparum*	37	*Eur. J. Med. Chem.*, 2017, **131**, 237
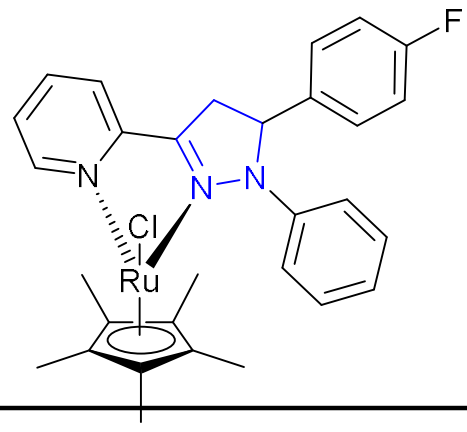	0.54µg/mL	DNA Intercalation	7	*J. Biomol. Struct. Dyn.*, 2016, **35**, 1599.
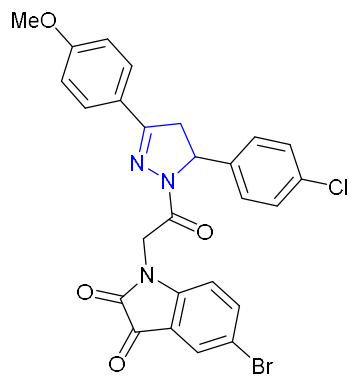	1.81 µM	Not determined	11	*Biopolym. Cell.*, 2020, **36**, 48
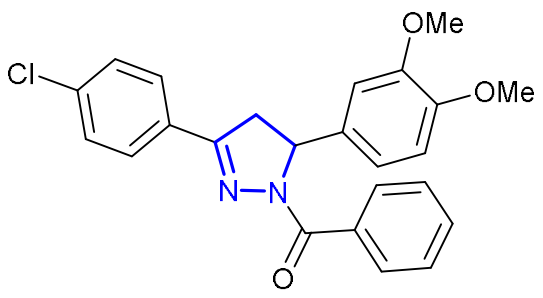	5.55 µM	Falcipain-2-receptor	4	*Trop. J. of Pharm. Res*, 2022, **21**, 1255
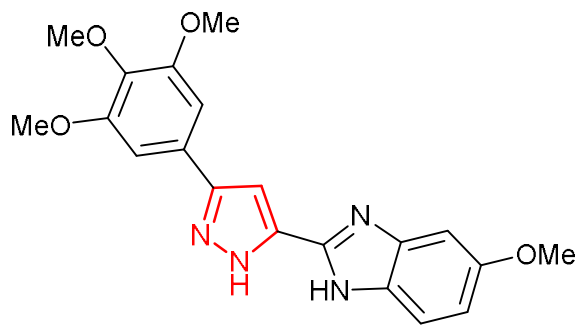	0.7 µM	Microtubule disruption	20	*Bioorg. Med. Chem.*, 2015, **23**, 1082.
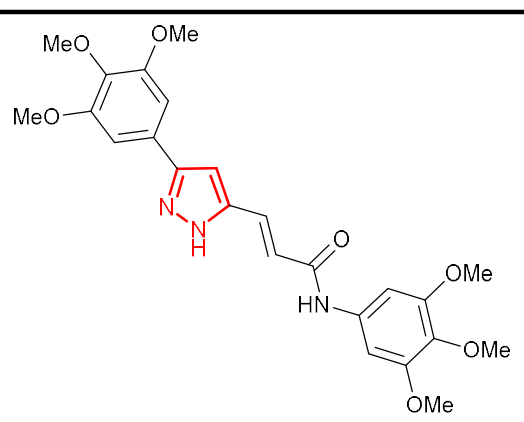	0.4 µM	Microtubule disruption	23	*Org. Biomol. Chem.*, 2015, **13**, 10162.
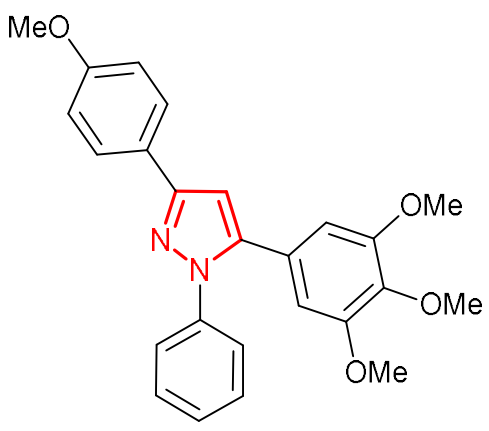	17.3 µM	Not determined	4	*Drug Res.*, 2021, **71**, 388.
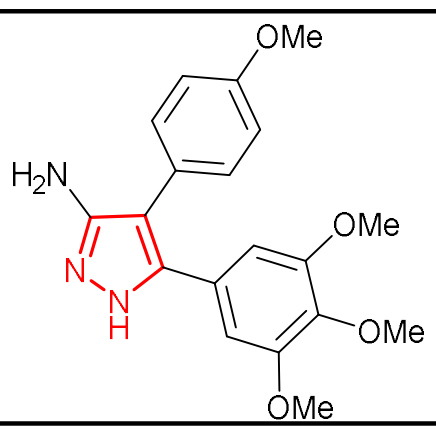	1.8 µM	Microtubule disruption	1	*Invest. New Drugs*, 2010, **28**, 493.
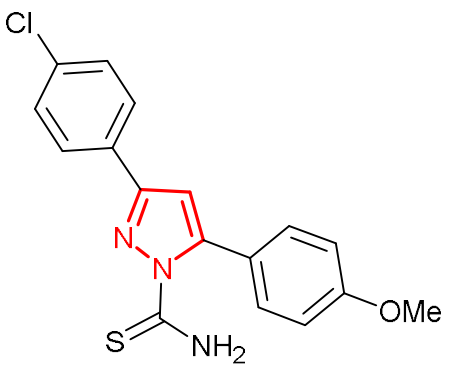	6.1 µM	VEGF	22	*Med. Drug Discovery*, 2025, **28**, 100227.

Lead compound	IC_{50}	Mode of action	No of compound reported	Reference
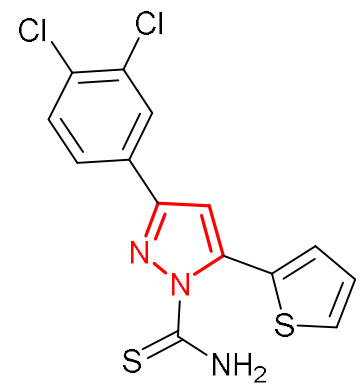	13.8 μM	VEGF	24	*Bioorg. Chem.*, 2022, **123**, 105770.
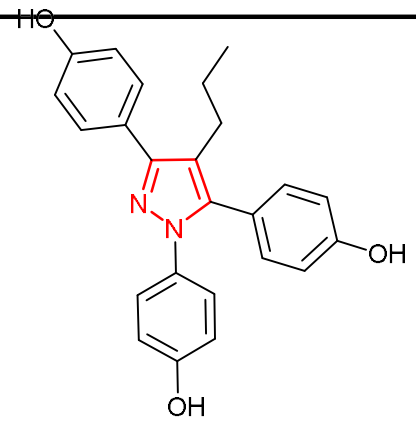	0.12 relative binding	Estrogen receptor	18	*J. Med. Chem.*, 2000, **43**, 4934.
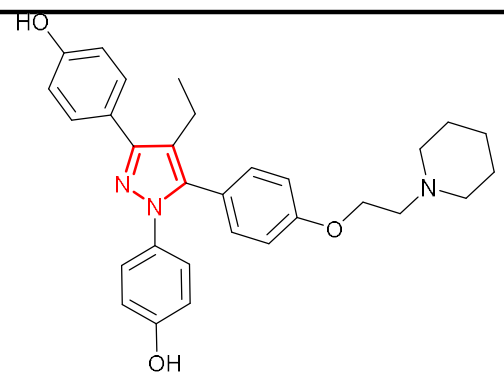	0.12 relative binding	Estrogen receptor	6	*Bioorg. Med. Chem.*, 2001, **9**, 151.
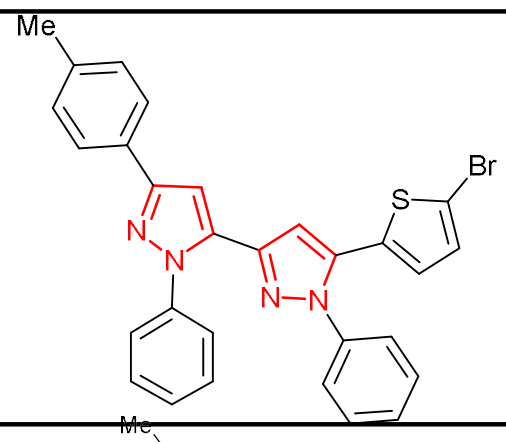	0.37 μM	COX-2	12	*Biorg. Med. Chem.*, 2004, **12**, 1935.
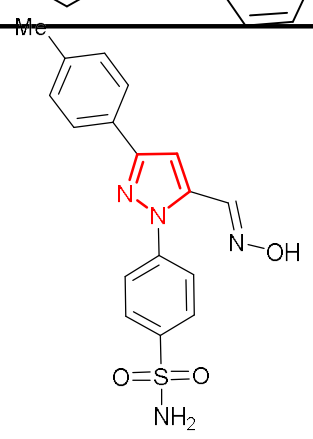	0.57 μM	COX-2	1	*Eur. J. Pharm. Sci.*, 2022, **168**, 106080.
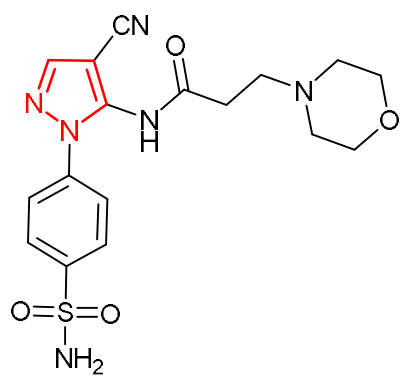	39 μM	COX-2	14	*Eur. J. Med. Chem.*, 2019, **171**, 332.
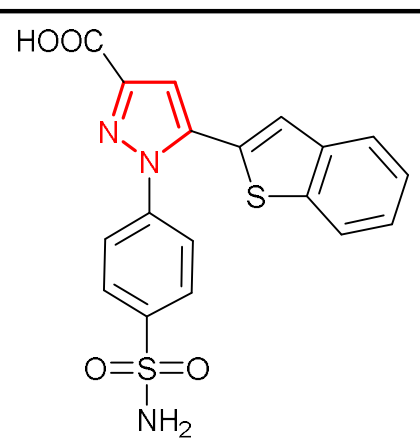	0.01 μM	COX-2	14	*Eur. J. Med. Chem.*, 2020, **189**, 112066.
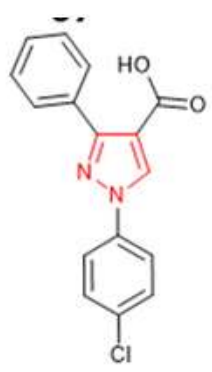	0.033 μM	Dihydrofolate reductase	6	*Arch. Pharm.*, 2012, **345**, 147.
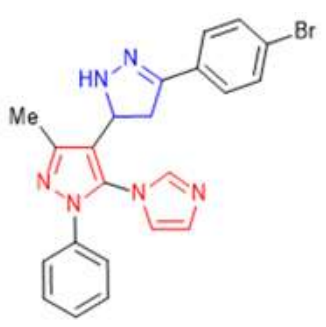	0.12 μg ml	Not determined	4	*Eur. J. Med. Chem.*, 2018, **163**, 353

Lead compound	IC_{50}	Mode of action	No of compound reported	Reference
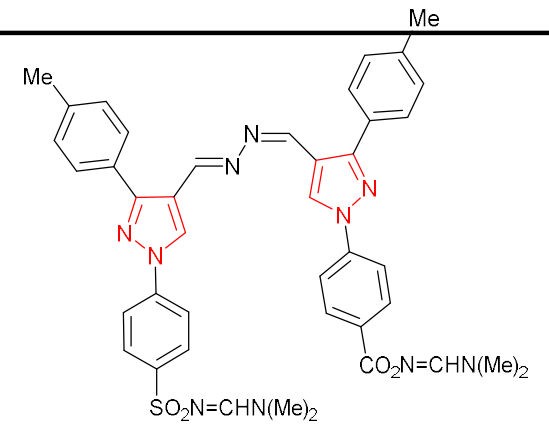	13.8 μM	Dihydrofolate reductase	3	*BMC Chemistry*, 2024, **18**, 9.
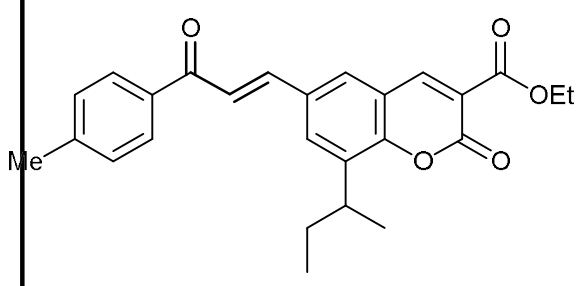	3.6 μM	Not determined	27	*Bioorg. Med. Chem.*, 2010, **20**, 7205
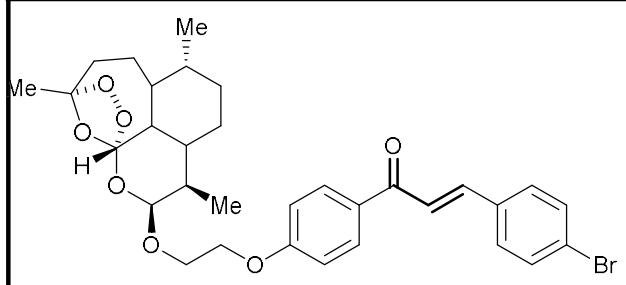	0.08 μM	Not determined	15	*Bioorg Med. Chem. Lett.* 2009, **19**, 4385
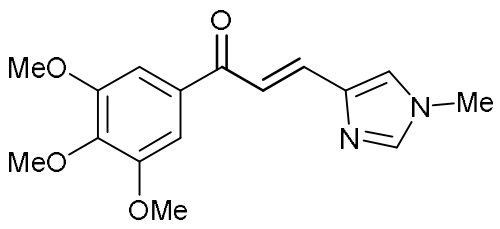	2.9 μM	Not determined	15	*Med. Chem. Commun.*, 2011, **2**, 1011.
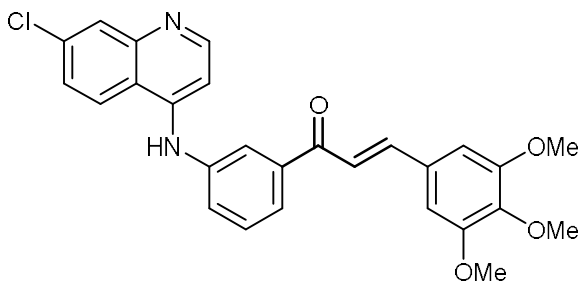	2.2 μM	Not determined	36	*Med. Chem. Res.*, 2019, **28**, 2050.

Lead compound	Analyte	Limit of Detection	No of compounds reported	Reference
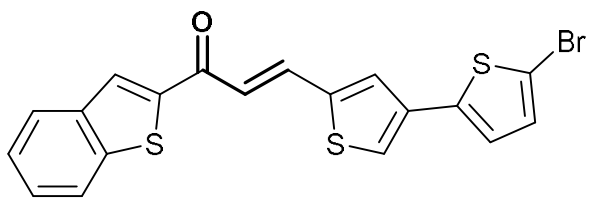	Fe^{3+}	Fe^{3+} 11.7 μM	1	*Luminescence*, 2024, **39**, e4823
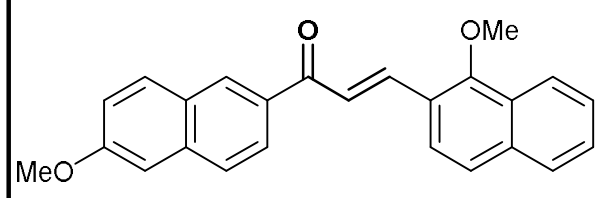	N/A	Not determined	1	*Sci. Rep.*, 2025, **15**, 31714.
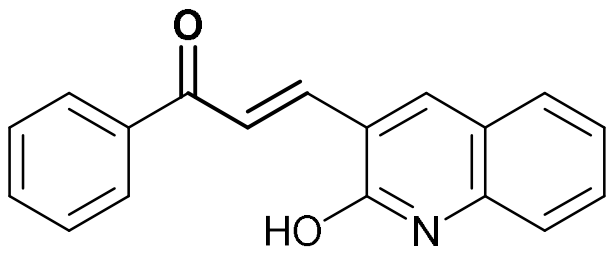	Fe^{3+}	Fe^{3+} 3.49 μM	1	*Anal. Methods*, 2014, **6**, 2883.
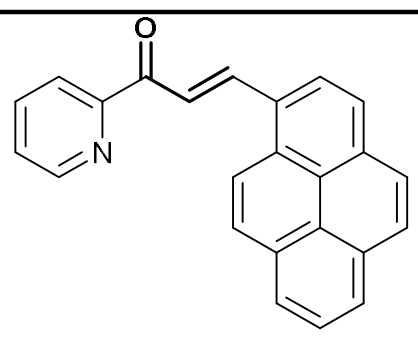	Ni^{2+}	Ni^{2+} 5.14 μM	1	*Sens. Actuators, B*, 2017, **238**, 306.
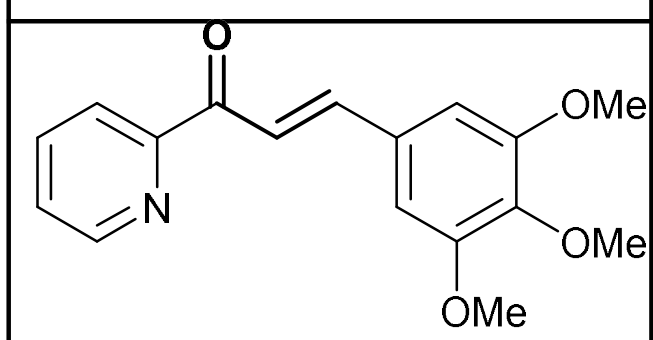	Fe^{3+}	Not determined	1	*J. Fluoresc*, 2020, **30**, 969.

Lead compound	Analyte	Limit of Detection	No of compounds reported	Reference
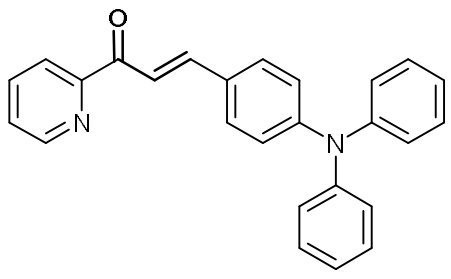	Pd^{2+}	Pd^{2+} 0.67 μM	1	*J. Fluoresc*, 2023, **33**, 1739
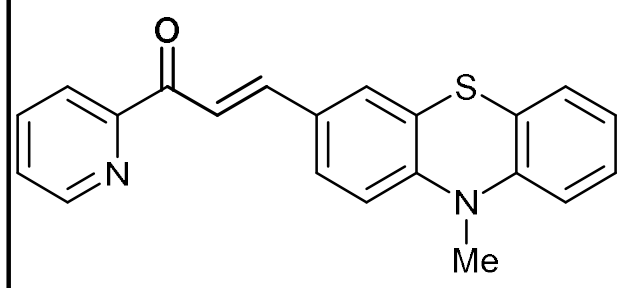	Cu^{2+}	Cu^{2+} 1.08 μM	1	*Tet. Lett.* 2008, **23**, 3739.
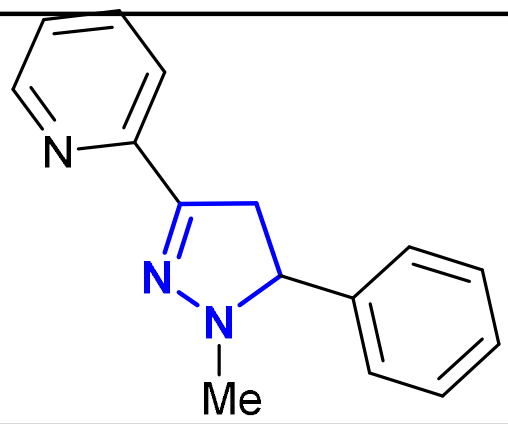	Zn^{2+}<br>Cd^{2+}	Zn^{2+} 0.20 M<br>Cd^{2+} 0.12 μM	2	*Org. Biomol. Chem.*, 2012, **10**, 8753.
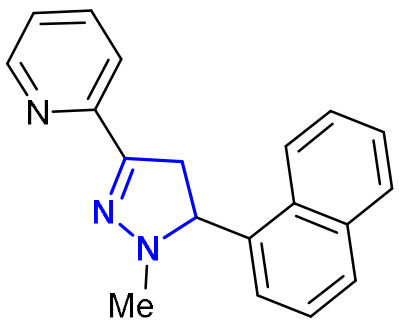	Fe3+	Not determined	4	*RSC Adv.*, 2024, **14**, 34918
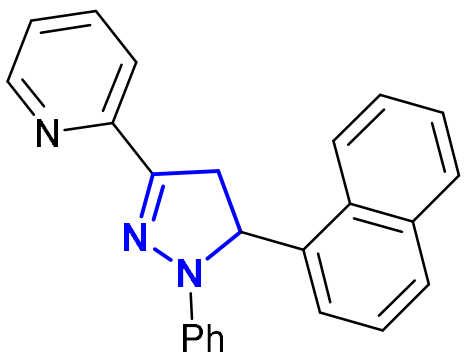	Fe^{3+}	Fe^{3+} 0.12 μM	4	*RSC Adv.*, 2024, **14**, 34918
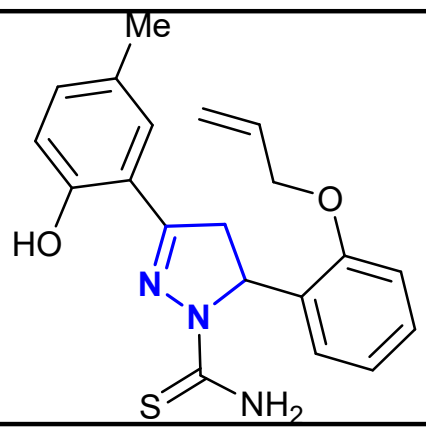	Zn^{2+}	Zn^{2+} 0.61 μM	1	*Org. Biomol. Chem.*, 2012, **10**, 8640.
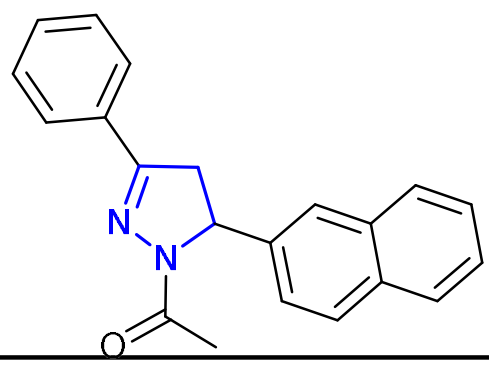	Zn^{2+}	Zn^{2+} 0.12 μM	1	*RSC Adv.*, 2014, **4**, 16973
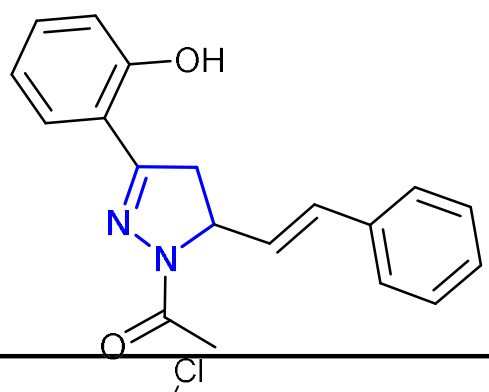	Zn^{2+}	Zn^{2+} 0.0295 μM	1	*Anal. Methods*, 2018, **10**, 1833.
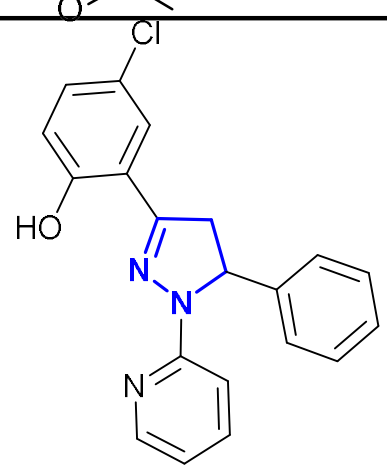	Zn^{2+}	Zn^{2+} 0.12 μM	1	*Sens. Actuators, B*, 2011, **159**, 148.

Lead compound	Analyte	Limit of Detection	No of compounds reported	Reference
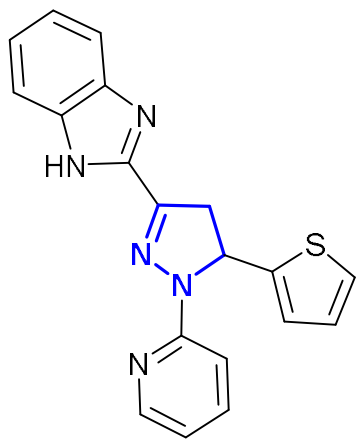	Ni^{2+}	Not determined	1	*Tet. Lett.* 2017, **58**, 1254.
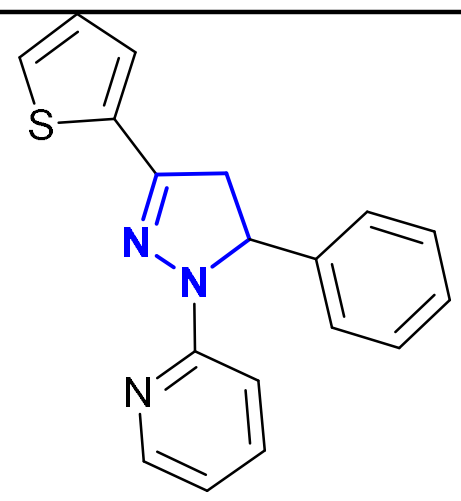	Cu^{2+}	Cu^{2+} 0.19 μM	1	*J. Fluoresc.*, 2013, **23**, 1263
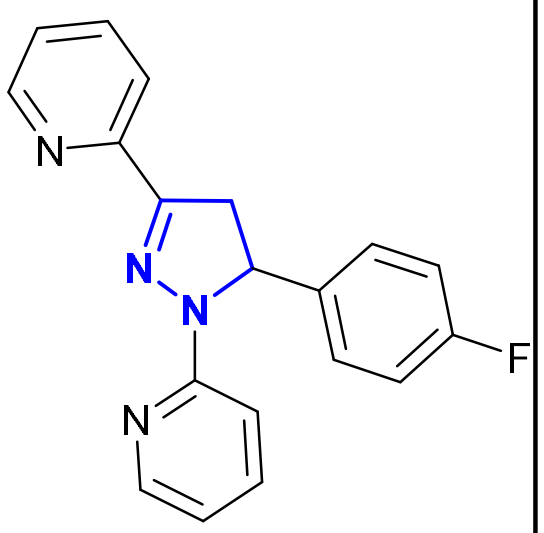	Zn^{2+}<br>Cd^{2+}	Not determined	11	*Org. Biomol. Chem.*, 2026, **24**, 873
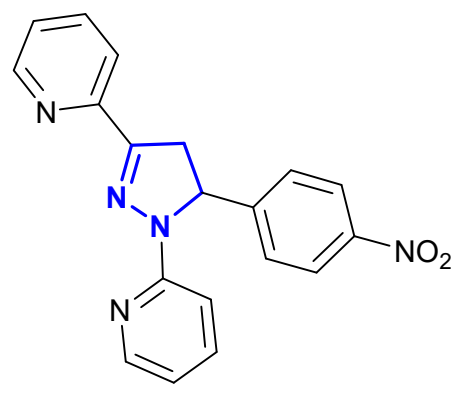	Zn^{2}	Not determined	11	*Org. Biomol. Chem.*, 2026, **24**, 873
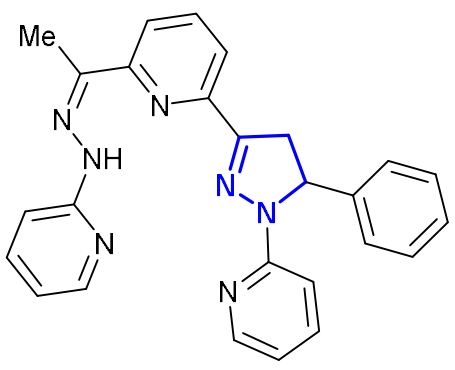	Zn^{2+}<br>Cd^{2+}	Zn^{2+}6.4 μM<br>Cd^{2+} 2.4 μM		*Anal. Methods*, 2025, **17**, 5782
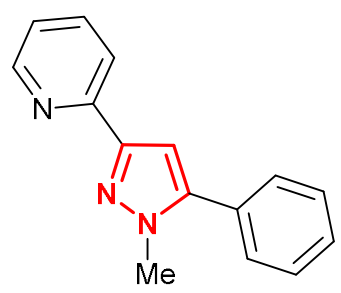	Zn^{2+}<br>Cd^{2+}	Zn^{2+} 0.24 μM<br>Cd^{2+} 0.34 μM	2	*Org. Biomol. Chem.*, 2012, **10**, 8753.
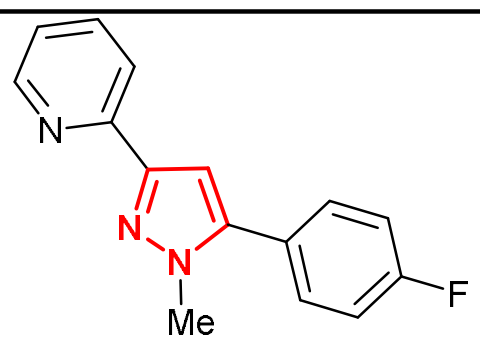	Zn^{2+}<br>Cd^{2+}	Zn^{2+} 1.97 μM<br>Cd^{2+} 0.077 μM	9	*New J. Chem.*, **48**, 13900
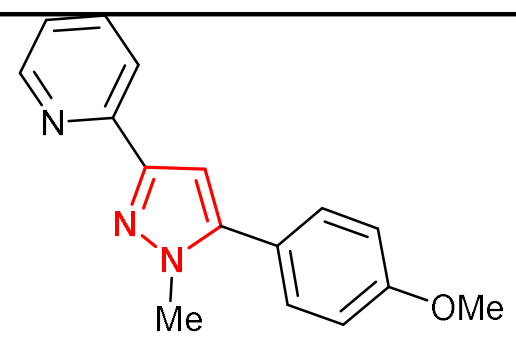	Zn^{2+}<br>Cd^{2+}	Not determined	9	*New J. Chem.*, **48**, 13900
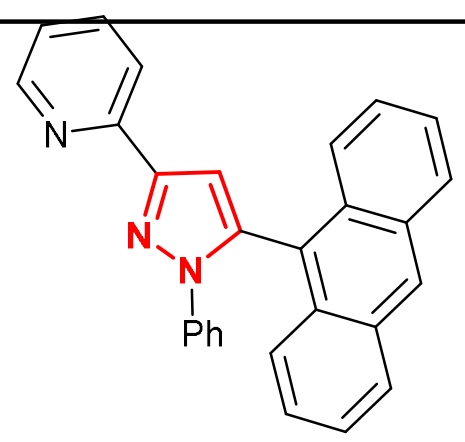	Fe^{3+}	Fe^{3+} 3.41 μM	3	*RSC Adv.*, 2024, **14**, 34918

Lead compound	Analyte	Limit of Detection	No of compounds reported	Reference
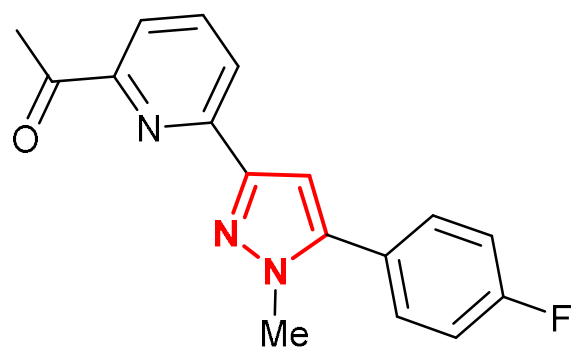	Zn^{2+}	Not determined	6	*RSC Adv.*, 2024, **14**, 3519
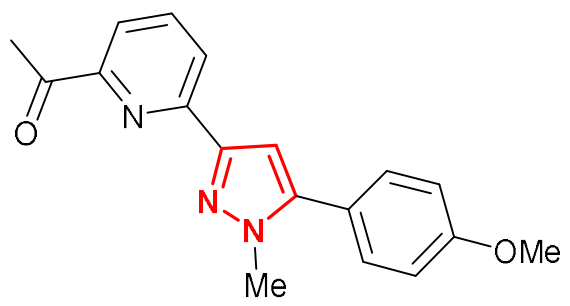	Fe^{3+}	Fe^{3+} 0.025 μM	6	*RSC Adv.*, 2024, **14**, 3519
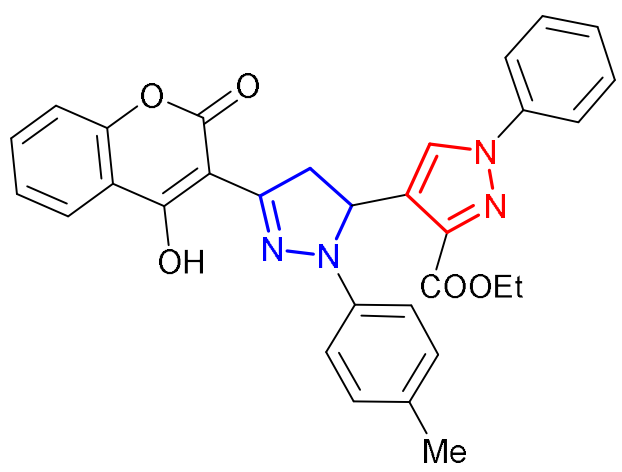	Cu^{2+}	Cu^{2+} 0.0053 μM	18	*RSC Adv.*, 2025, **15**, 48529
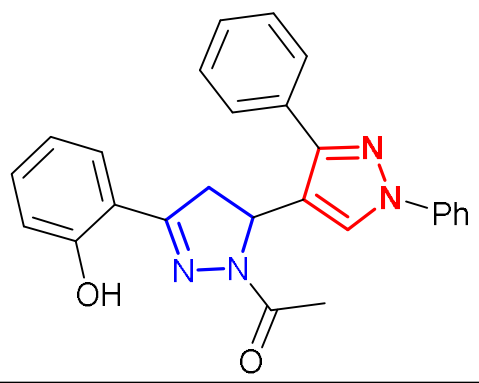	Fe^{3+}	Fe^{3+} 0.00039 μM	1	*J. Fluoresc.*, 2021, **31**, 29
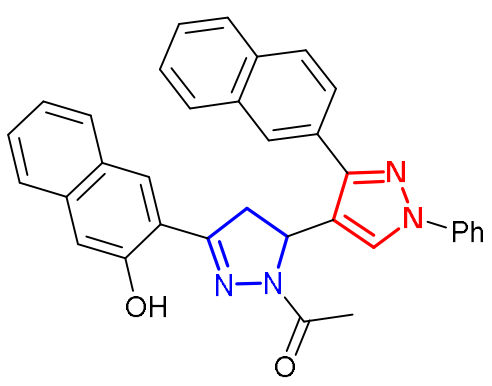	Fe^{3+}	Fe^{3+} 2.33 μM	1	*Spectrochim. Acta, Part A*, 2025, **329**, 125533
